# Dual Repression by IPA1 Fine‐Tunes OsbZIP79‐Mediated Salt Tolerance in Rice

**DOI:** 10.1002/advs.77806

**Published:** 2026-09-16

**Authors:** Hui Wang, Bo Liu, Can Hu, Chenfan Zheng, Yuting Wei, Xier Xu, Wei Yang, Yu Fang, Guangle Shen, Pedro García‐Caparros, Conghe Zhang, Qingyao Shu, Meng Jiang

**Affiliations:** ^1^ National Key Laboratory of Rice Biology The Advanced Seed Institute Zhejiang University Hangzhou Zhejiang Province People's Republic of China; ^2^ Win‐All Hi‐Tech Seed, Co. Hefei Anhui Province People's Republic of China; ^3^ Hainan Institute Zhejiang University Sanya Hainan Province People's Republic of China; ^4^ Zhejiang Provincial Key Laboratory of Crop Germplasm Innovation and Utilization The Advanced Seed Institute Zhejiang University Hangzhou Zhejiang Province People's Republic of China; ^5^ Higher Polytechnic School University of Almeria Almeria Spain

**Keywords:** IPA1, Na^+^/K^+^ ratio, OsbZIP79, redox homeostasis, rice, salt response

## Abstract

Soil salinity severely impairs rice yield and quality, yet the molecular mechanisms of its salt tolerance remain unclear. In this study, we first discovered OsbZIP79, a novel basic leucine zipper (bZIP)‐type transcription factor, as a positive regulator of salt tolerance in rice. We uncovered that OsbZIP79 positively regulates the expression of *OsHAK26* (encoding a potassium transporter) and *OsMPG1* (encoding a mannose‐1‐phosphate guanylyltransferase) to confer salt tolerance in rice. In vivo and in vitro evidence demonstrate that OsbZIP79 physically interacts with Ideal Plant Architecture1 (IPA1), a known negative regulator of salt stress responses. IPA1 represses the expression of *OsbZIP79* and competes with the OsbZIP79 C‐terminus for binding to the promoters of its target genes. Haplotype analysis of 4726 rice accessions identified Hap3 of *OsbZIP79* as a favorable allele that confers significantly enhanced salinity tolerance compared to Hap1 and Hap2. These findings reveal a novel salt tolerance regulatory network governed by the OsbZIP79‐IPA1 module. This work provides insights to facilitate the improvement of salt tolerance in rice through advanced breeding.

## Graphical abstract

Dual repression by IPA1 fine‐tunes OsbZIP79‐mediated salt tolerance in rice: direct transcriptional inhibition under normal conditions and salt‐induced degradation under stress. This dual mechanism activates OsbZIP79 to regulate Na^+^/K^+^ homeostasis and redox balance via downstream genes, enabling optimal salt stress response.

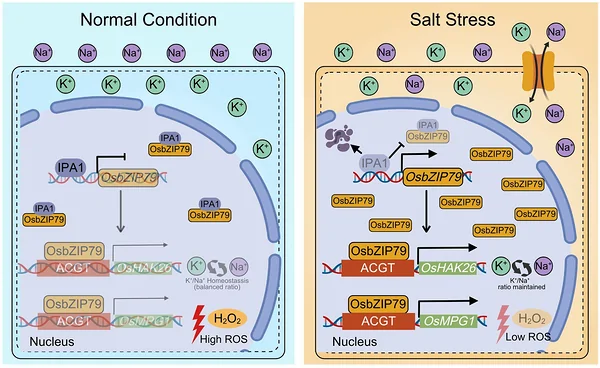

## 1 Introduction

Salt stress represents a significant abiotic challenge that diminishes global agricultural yields, leading to enormous financial losses each year [1]. Unlike transient stresses such as drought, cold, or heat, which can either occur suddenly or be addressed through timely agricultural interventions, salinity constitutes a chronic stress that persists within agricultural ecosystems [2]. Salt stress compromises both the quality and yield of agricultural products, such as fibers, fruits, and seeds [3]. Consequently, the cultivation of new rice varieties with heightened salt tolerance stands as a critical method to enhance agricultural resilience in salt‐affected regions [4]. Utilizing modern genetic breeding techniques to harness novel salt‐tolerant genes in rice and decipher their underlying molecular regulatory mechanisms represents a pivotal strategy for addressing the detrimental impacts of salinity [5].

In recent years, researchers have conducted in‐depth studies on the identification of salt‐tolerant related genes and their molecular regulatory networks in rice [6]. Salt‐tolerant related genes, such as *OsSKC1* [7], have been successfully mapped and cloned, contributing to a growing catalog of genetic components involved in salinity adaptation [8]. Under salt stress, sodium (Na^+^) enters mature epidermal cells through non‐selective cation channels (NSCCs), causing depolarization of the cell membrane and being polarized by the activity of P‐type ATPases [9, 10]. The Na^+^/potassium (K^+^) ratio serves as a pivotal marker for assessing plant's resilience to salt stress [9]. When plants are subjected to salt stress, an influx of Na^+^ into the cells occurs, often at the expense of K^+^ levels [11]. Thus, preserving cellular Na^+^/K^+^ homeostasis is essential for a plant's ability to endure and counteract the deleterious impacts of salt‐induced ionic stress [5]. Various protein transporters, functioning as pumps and/or channels, are known to play a role in maintaining Na^+^/K^+^ ion homeostasis in rice under saline conditions. The overexpression of genes involved in ion homeostasis, such as *OsNHX1* (a vacuolar Na^+^/hydrogen (H^+^) antiporter gene; [12]), *OsKAT1* (a K^+^ channel gene; [13]), *OsARP* (a vacuolar antiporter regulating protein gene; [14]), *OsVP1* (a vacuolar H^+^‐translocating inorganic pyrophosphatase gene; [15]), *OsHAK5* (a K^+^ transporter gene; [16]), *OsHKT2;1* (a Na^+^ transporter gene; [17]), and *OsSOS1* (a plasma membrane Na^+^/H^+^ exchanger gene; [18]), have been shown to enhance salt tolerance in transgenic rice by promoting seedling growth, photosynthetic efficiency, root development, and ionic homeostasis. Therefore, these transporters play an important role in maintaining the Na^+^/K^+^ homeostasis of rice during salt stress, underscoring the need for further research into the molecular mechanisms governing their regulation. Salt stress induces a surge in the concentration of cytoplasmic second messengers, including calcium (Ca^2+^), phosphatidic acid (PA), and reactive oxygen species (ROS) [3]. Through their role in scavenging ROS and maintaining redox homeostasis, antioxidative genes have been implicated in the salt tolerance mechanism of rice. Rice plants genetically modified to overexpress *OsMSRA4.1* (a plastidial methionine sulfoxide reductase gene; [19]), *OsVTE1* (a rice tocopherol cyclase ortholog gene; [20]), or *OsECS* (a γ‐glutamylcysteine synthetase gene; [21]) demonstrate enhanced resilience to salt stress. Furthermore, various studies have documented the upregulation of antioxidative genes such as *OsAPX7* (a chloroplast ascorbate peroxidase gene; [22]), *OsAPX8* (a chloroplast ascorbate peroxidase gene; [23]), *OsGR2,3* (a glutathione reductase gene; [24]), *OsMIOX* (a myo‐inositol oxygenase gene; [25]), and *OsSRO1c* (similar to radical‐induced cell death one (SRO) protein gene; [26]) in response to salt stress, underscoring their active involvement in maintaining cellular redox homeostasis. Antioxidative genes play an important role in rice salt stress response, and the underlying molecular mechanisms urgently need to be explored.

Rice activates a chain of response pathways and signal transduction processes to control the expression of downstream genes via transcription factors, which are instrumental in the plant's defense against environmental stressors [3]. Under salt stress, the concentration of jasmonic acid (JA) rises, with SCFCOI1 recruiting OsJAZ9 for ubiquitination and subsequent degradation through the 26S proteasome. This degradation releases OsbHLH062 (basic helix‐loop‐helix protein62) and OsNINJA, enabling the activation of target genes, thereby facilitating the plant's adaptation to salt stress [27]. Concurrently, OsMPK4 (Mitogen‐activated protein Kinase 4) is activated and phosphorylates IPA1 (IDEAL PLANT ARCHITECTURE1) at the Thr180 site, promoting its ubiquitination and degradation, which reduces the protein level of IPA1 and ultimately enhances salt tolerance in rice [28]. OsSAE1 (SALT AND ABA RESPONSE ERF1) directly binds to the promoter of *OsABI5* (*ABSCISIC ACID‐INSENSITIVE5*), thereby repressing its expression. As a central regulatory component in the ABA signaling pathway, *OsABI5* negatively influences rice seed germination and salt tolerance [29]. OsSAPK10 (Stress‐Activated Protein Kinase10) has been shown to interact with and phosphorylate OsbZIP20 (bZIP transcription factor 20), which in turn binds to the ABA‐responsive element (ABRE) element within the *OsNHX1* promoter, triggering its transcription and thereby bolstering rice's salt stress resistance [30]. Additionally, research has identified OsWRKY53 as a pivotal regulatory factor in rice's response to salt stress, capable of orchestrating the expression of the *OsMKK10.2* (*Mitogen‐activated protein Kinase Kinase 10.2*) and *OsHKT1;5* (*Na^+^ transporter1;5*) genes to coordinate rice's salt tolerance [31]. Overall, these findings highlight the complexity of the gene regulatory networks underlying rice responses to salt stress, which involve numerous signaling pathways and transcriptional regulators. The expression of certain transcription factors is also highly responsive to salt stress, though their specific regulatory modules and pathways remain to be fully explored.

In this study, we identified OsbZIP79 as a key positive regulator of salt tolerance in rice seedlings. Utilizing an integrated approach combining genome‐wide screening and transcriptome sequencing (RNA‐Seq), we demonstrated that OsbZIP79 directly activates the expression of *OsHAK26* (a K^+^ transporter gene) and *OsMPG1* (a mannose‐1‐phosphate guanyl transferase gene) in response to salt stress. Additionally, OsbZIP79 was found to physically interact with IPA1, which negatively regulated OsbZIP79 expression. IPA1 engaged in competitive interaction with the C‐terminus of the OsbZIP79 protein, thereby repressing the binding of OsbZIP79's C‐terminus to the promoters of its target genes. These findings underscore the OsbZIP79‐IPA1 module as a critical molecular switch in the signaling cascade governing rice salt tolerance, and they enrich our comprehension of the interplay among transcription factors, redox homeostasis, and Na^+^/K^+^ ratio signaling pathways, which fine‐tune the balance between plant growth and stress adaptation.

## 2 Results

### 2.1 OsbZIP79 Positively Modulates Salt Tolerance in Rice

**FIGURE 1:**
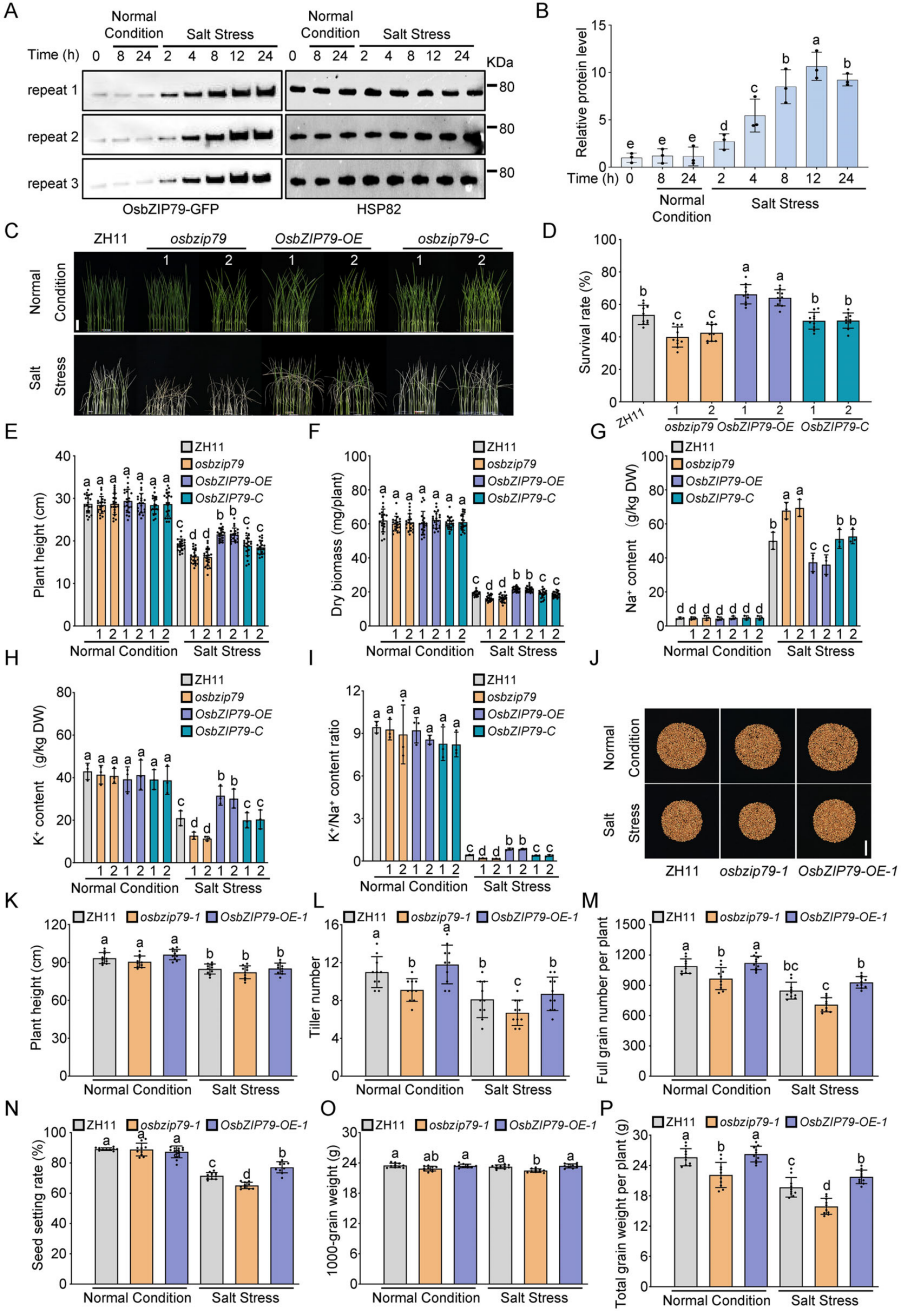
*OsbZIP79* positively regulates rice salt tolerance. (A) OsbZIP79 protein abundance in leaves of 14‐day‐old *osbzip79‐C1* seedlings exposed to salt stress (150 mm NaCl) for the indicated times. OsbZIP79‐GFP was detected using anti‐GFP antibody, with HSP82 serving as a loading control. (B) Quantification of immunoblot results shown in (A) using ImageJ software. OsbZIP79 abundances were normalized to Actin and shown as relative to the level in *osbzip79‐C1* seedlings grown at normal conditions, which was set to 1.0. (C‐I) Phenotypes (C), survival rates (D), plant height (E), dry biomass (F), shoot Na^+^ content (G), shoot K^+^ content (H), and shoot K^+^/Na^+^ content ratios (I) in ZH11, *osbzip79*, *OsbZIP79‐OE*, and *osbzip79‐C*. (J‐P) Agronomic traits of ZH11 (wild type), *osbzip79* (mutant), and *OsbZIP79‐OE* (overexpression line) plants under salt stress. (J) Whole‐plant grain yield phenotype. (K) Plant height. (L) Tiller number. (M) Full grain number per plant. (N) Seed‐setting rate. (O) 1,000‐grain weight. (P) Grain yield per plant. Values are means ± standard deviation (SD) (*n* = 3 in B, G, H and I; *n* = 10 in K, L, M, N, O and P; *n* = 20 in E and F). Scale bar = 5 cm. Different letters indicate significant differences (*p* < 0.05).

To delineate the response of *OsbZIP79* to saline stress, we quantified the gene expression levels of *OsbZIP79* in rice seedlings over a 24‐h period following exposure to 150 mM NaCl. Our data revealed a consistent upregulation of *OsbZIP79* expression across rice leaves, roots, and shoots throughout this timeframe (Figure S1). Additionally, immunoblot analyses were conducted to assess the impact of salt treatment on OsbZIP79 protein levels. The results indicated a substantial alignment between the protein and transcript levels of OsbZIP79, with both being significantly elevated in salt‐treated seedlings compared to their untreated counterparts (Figure 1A,B). These findings collectively suggest that *OsbZIP79* is a gene responsive to salt stress in rice.

To investigate the role of *OsbZIP79* in salt tolerance, we studied two loss‐of‐function mutants, *osbzip79‐1* and *osbzip79‐2*, derived from the ZH11 background [32]. Upon salt treatment, both ZH11 and its *osbzip79* mutants exhibited a decline in survival rate, plant height, and dry biomass, with the mutants showing a more severe reduction than ZH11 (Figure 1C–F). Specifically, the survival rate, plant height, and dry biomass of the *osbzip79* mutants were approximately 14.4% to 25.1% lower than those of ZH11 (Figure 1D‐F). Furthermore, we generated two *OsbZIP79* overexpressing (OE) lines, *OsbZIP79‐OE1* and *OsbZIP79‐OE2*, which encodes a fusion protein of OsbZIP79 and green fluorescent protein (GFP) [32]. After salt treatment, both OE lines displayed superior growth, along with higher survival rates, plant height, and dry biomass compared to ZH11 (Figure 1C–F). These findings unequivocally establish the significant contribution of *OsbZIP79* to salt tolerance in rice. To confirm that the diminished performance of *osbzip79‐1* and *osbzip79‐2* mutants was specifically attributable to the knockout of *OsbZIP79*, we generated two complementation lines, *osbzip79‐C1* and *osbzip79‐C2* [32]. These complementation lines exhibited phenotypes resembling those of ZH11 under both normal and salt‐stress conditions, with negligible differences in survival rate, plant height, and dry biomass (Figure 1C‐F). These findings collectively demonstrated that the reduced salt tolerance observed in *osbzip79‐1* and *osbzip79‐2* mutants was indeed a consequence of the *OsbZIP79* knockout.

To elucidate the underlying mechanism through which *OsbZIP79* responds to salt stress, we assessed two critical indicators: the K^+^/Na^+^ ratio and redox homeostasis. Initially, we measured the total Na^+^ and K^+^ contents to discern the role of OsbZIP79 in regulating the K^+^/Na^+^ ratio in rice shoots under salt stress (Figure 1G–I). Under normal conditions, there were no significant changes in the contents of Na^+^ and K^+^ or the K^+^/Na^+^ ratio among the loss‐of‐function mutants, OE lines, complementation lines, and WT (Figure 1G–I). However, under salt stress, the Na^+^ content was substantially induced by 35.5% and 38.6% in *osbzip79* mutants and significantly decreased by 25.3% and 27.9% in *OsbZIP79‐OE* lines compared to WT (Figure 1G). Conversely, the K^+^ content and the K^+^/Na^+^ ratio in rice shoots were notably lower in mutants than in WT, but significantly increased in OE lines under salt stress (Figure 1H,I). Subsequently, we evaluated the concentrations of H_2_O_2_ and the activities of antioxidant enzymes (SOD, CAT, and GST) in rice shoots to further understand how *OsbZIP79* alleviates salt stress through redox homeostasis regulation (Figure S2). Under normal conditions, there were no significant changes in H_2_O_2_ content or antioxidant enzyme activities among the different lines (Figure S2). Salt stress induced oxidative stress, as evidenced by increased H_2_O_2_ levels and decreased antioxidant enzyme activities (SOD, CAT, and GST), which are detrimental to plant cells and cause substantial damage (Figure S2). Specifically, H_2_O_2_ content in rice leaves increased by 22.0% and 22.1% in *osbzip79* mutants, but was reduced by 19.4% and 21.9% in *OsbZIP79‐OE* lines compared to WT under salt stress (Figure S2). Consistently, antioxidant enzyme activities were significantly reduced in mutants but enhanced in OE lines relative to WT under salt stress (Figure S2B–D). These findings demonstrate that *OsbZIP79* enhances salt stress tolerance by regulating Na^+^/K^+^ homeostasis and redox balance in rice.

To further elucidate the role of *OsbZIP79* in rice salt tolerance across the entire growth cycle, we evaluated key agronomic traits at the mature stage under both normal and salt stress conditions (Figure 1J–P). Under normal conditions, no significant differences were observed in plant height, seed setting rate, and 1000‐grain weight among the wild‐type (ZH11), *osbzip79‐1* mutant, and *OsbZIP79‐OE‐1* line. However, salt stress markedly impacted these traits, with distinct phenotypic differences among the genotypes. Specifically, the *osbzip79‐1* mutant exhibited reduced tiller number, filled grain number per plant, seed setting rate, 1000‐grain weight, and total grain weight per plant compared to ZH11 under salt stress. In contrast, the *OsbZIP79‐OE‐1* line maintained superior performance, with significantly higher values for these traits‐especially the grain yield per plant (Figure 1J,P)‐than ZH11 under salt stress. These results collectively demonstrate that *OsbZIP79* positively modulates salt tolerance not only during the seedling stage but also at the mature stage, highlighting its critical role in maintaining yield‐related traits under saline conditions.

### 2.2 OsbZIP79 Enhances Salt Stress Tolerance by Activating its Target Genes

To delve into the molecular mechanisms underlying OsbZIP79's contribution to salt tolerance, we conducted a range of molecular analyses. Initially, we performed comparative transcriptome sequencing (RNA‐Seq) on 14‐day‐old seedlings of wild‐type ZH11 and the *osbzip79‐1* mutant, both subjected to 150 mm NaCl for 24 h. This analysis revealed 9,637 differentially expressed genes (DEGs) in *osbzip79‐1* relative to ZH11 (Figure S3A), using a significance threshold of |log2FC| > 1.5 and padj < 0.05 (Figure S3B). Among these DEGs, 3,131 genes were down‐regulated whereas 6,506 were up‐regulated (Figure S3B).

Given the lack of identified target genes directly regulated by OsbZIP79, we proceeded with DNA affinity purification sequencing (DAP‐Seq) using OsbZIP79 to characterize its genome‐wide binding landscape. This experiment identified 55,755 peaks distributed across all 12 rice chromosomes (Figure S4A,B). Notably, 50.8% of these peaks were located within genic regions, including gene bodies and 2‐kb regions flanking the transcription start sites (TSS), with 24.2% specifically enriched in promoter regions (Figure S4C). Motif analysis of the 2‐kb sequences surrounding the peaks identified an over‐represented motif with the core sequence ‘ACGT’ (Figure S4D), consistent with canonical binding sites of bZIP transcription factors and indicative of potential OsbZIP79 target loci. Furthermore, Gene Ontology (GO) enrichment analysis of the putative target genes revealed significant overrepresentation of pathways associated with transmembrane transport and oxidative stress response (Figure S4E).

**FIGURE 2:**
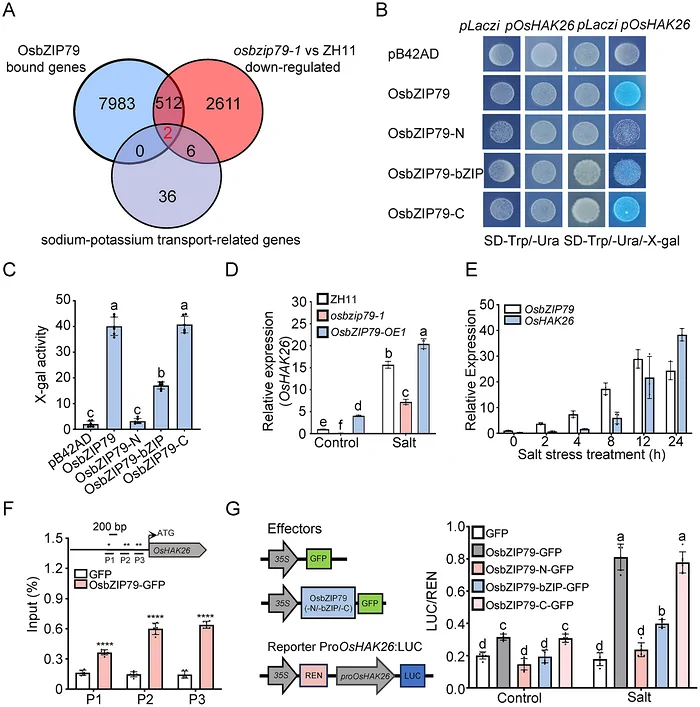
OsbZIP79 directly regulates the expression of *OsHAK26* under salt stress. (A) Venn diagram showing the comparison of down‐regulated DEGs in *osbzip79‐1* and ZH11 under salt stress with reported sodium‐potassium transport‐related genes. (B‐C) Yeast one‐hybrid (Y1H) with X‐gal assay reveals stronger regulation of *OsHAK26* by the OsbZIP79 C‐terminus compared to the bZIP and N‐terminal domains. (D) *OsHAK26* expression of ZH11, *osbzip79‐1*, and *OsbZIP79‐OE1*. (E) Relative expression levels of *OsbZIP79* and *OsHAK26* in ZH11 plants under salt stress. (F) ChIP‐qPCR assay to detect the binding of OsbZIP79 to the motifs in the *OsMPG1* promoter. (G) OsbZIP79 C‐, bZIP‐, and N‐terminal domains on the *OsHAK26* promoter were evaluated by a dual luciferase reporter (DLR) assay. The core constructs of the vectors are shown on the left. Values are means ± standard deviation (SD) (*n* = 3 in D and E; *n* = 6 in C, F, and G). Different letters indicate significant differences (*p* < 0.05). ^*^, ^***,^ and ^****^ indicate *p* < 0.05, 0.001, and 0.0001; ns indicates not significant.

Building on our initial physiological and biochemical assessments (Figure 1; Figure S2) and RNA‐Seq data analysis (Figure S3), we identified a potential link between OsbZIP79 and the regulation of Na^+^/K^+^ balance and redox homeostasis in rice under salt stress. To pinpoint genes directly regulated by OsbZIP79 that play a role in Na^+^/K^+^ homeostasis under salt stress, we conducted an integrated analysis combining three datasets: 1) the 3,131 down‐regulated genes in the *osbzip79‐1* mutant compared to ZH11, 2) 8,479 genes with the ‘ACGT’ motif within their promoter regions, and 3) the Na^+^/K^+^ transport‐related genes (Figure 2A). This comprehensive approach revealed two genes of interest, *LOC_Os08g39950* and *LOC_Os01g20160* (Figure 2A; Figure S5A). RNA‐Seq heatmap analysis showed that both genes were significantly downregulated in the *osbzip79‐1* mutant under salt stress (Figure S5A). Consistently, DAP‐Seq analysis visualized by Integrative Genomics Viewer (IGV) revealed clear binding peaks of OsbZIP79 in the promoter regions of these candidate genes (Figure S5B). However, yeast‐one‐hybrid (Y1H) analyses showed that OsbZIP79 only binds to the promoter of *LOC_Os08g39950* (annotated as *OsHAK26*, which potentially encodes a potassium (K^+^) transporter(Figure S5C). In contrast, Y1H and dual‐luciferase reporter (DLR) assays demonstrated that OsbZIP79 does not bind to the promoter of *LOC_Os01g20160* (Figure S5C,D). Further analyses showed that it is the C‐terminal region of OsbZIP79 that directly binds to the *OsHAK26* promoter (Figure 2B,C).

To assess the effect of OsbZIP79 on the transcription of *OsHAK26*, we examined its transcript abundance in the knockout (*osbzip79‐1*) and overexpressing (*OsbZIP79‐OE1*) lines of *OsbZIP79*, together with WT (ZH11) under both normal and salt stress conditions. *OsHAK26* transcription was significantly enhanced in the OE line, while greatly reduced in the knockout mutant compared to WT (Figure 2D). Time‐course expression analysis revealed that the salt‐induced *OsbZIP79* transcription precedes that of *OsHAK26* (Figure 2E), which fits the role of OsbZIP79 as a regulator of *OsHAK26*.

To further elucidate its regulatory mechanism, we further performed chromatin immunoprecipitation quantitative PCR (ChIP‐qPCR) and DLR assays. ChIP‐qPCR showed that the P1, P2, and P3 amplicons from the *OsHAK26* promoter, each containing an ‘ACGT’ motif (Figure S6), were significantly enriched in the DNA immunoprecipitated with OsbZIP79‐GFP (Figure 2F). Consistently, DLR assays also demonstrated that both the full‐length OsbZIP79 and its C‐terminal region significantly enhanced luciferase activity driven by the *OsHAK26* promoter under both control and salt stress conditions, with a more pronounced enhancement observed under salt stress (Figure 2G; Figure S5). These results indicate that OsbZIP79, via its C‐terminus, directly binds to the *OsHAK26* promoter to activate its transcription.

**FIGURE 3:**
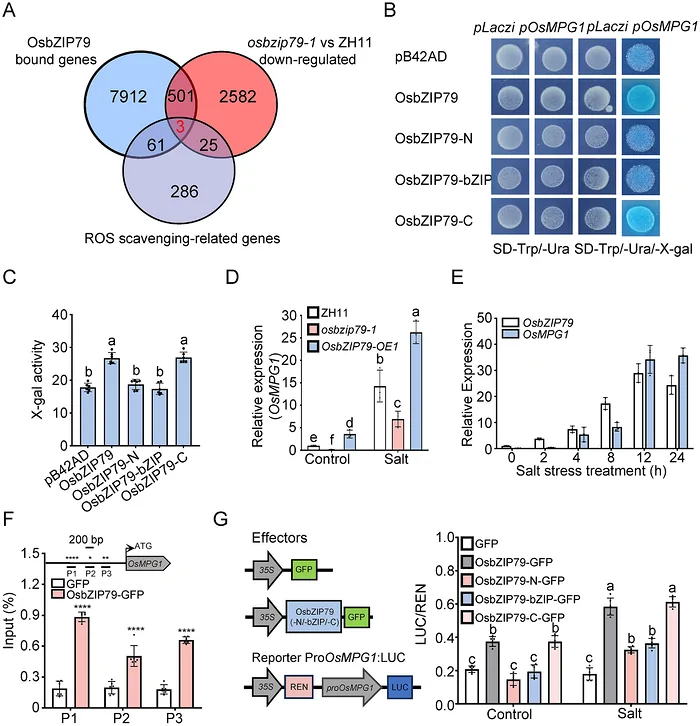
OsbZIP79 directly regulates the expression of *OsMPG1* under salt stress. (A) Venn diagram showing the comparison of down‐regulated DEGs in *osbzip79‐1* and ZH11 under salt stress with reported sodium‐potassium transport‐related genes. (B‐C) Yeast one‐hybrid (Y1H) with X‐gal assay reveals stronger regulation of *OsMPG1* by the OsbZIP79 C‐terminus compared to the bZIP and N‐terminal domains. (D) *OsMPG1* expression of ZH11, *osbzip79‐1*, and *OsbZIP79‐OE1*. (E) Relative expression levels of *OsbZIP79* and *OsMPG1* in ZH11 plants under salt stress. (F) ChIP‐qPCR assay to detect the binding of OsbZIP79 to the motifs in the *OsMPG1* promoter. (G) OsbZIP79 C‐, bZIP‐, and N‐terminal domains on the *OsMPG1* promoter were evaluated by a dual‐luciferase reporter (DLR) assay. The core constructs of the vectors are shown on the left. Values are means ± standard deviation (SD) (*n* = 3 in D and E; *n* = 6 in C, F, and G). Different letters indicate significant differences (*p* < 0.05). ^*^, ^***^, and ^****^ indicate *p* < 0.05, 0.001, and 0.0001; ns indicates not significant.

Through joint analysis incorporating known ROS scavenging‐related genes, we identified the following three genes, *LOC_Os01g62840*, *LOC_Os01g10110*, and *LOC_Os04g09920*, as candidate genes regulated by OsbZIP79(Figure 3A; Figure S7A). RNA‐Seq heatmap analysis revealed downregulation of these three genes in the *osbzip79‐1* mutant under salt stress (Figure S7A). DAP‐Seq IGV analysis showed clear OsbZIP79 binding peaks in the promoters of these genes (Figure S7B). Y1H analyses, however, identified only *LOC_Os01g62840*, annotated as an OsMPG1 (mannose‐1‐phosphate guanyl transferase) encoding gene, as a direct transcriptional target of OsbZIP79 (Figure S7C), because OsbZIP79 seemed not to bind to the promoters of *LOC_Os01g10110* and *LOC_Os04g09920* in Y1H and DLR assays (Figure S7C,D). In contrast, the direct binding of the OsbZIP79 C‐terminus to the *OsMPG1* promoter was verified via Y1H and X‐gal activity assays (Figure 3B,C).

**FIGURE 4:**
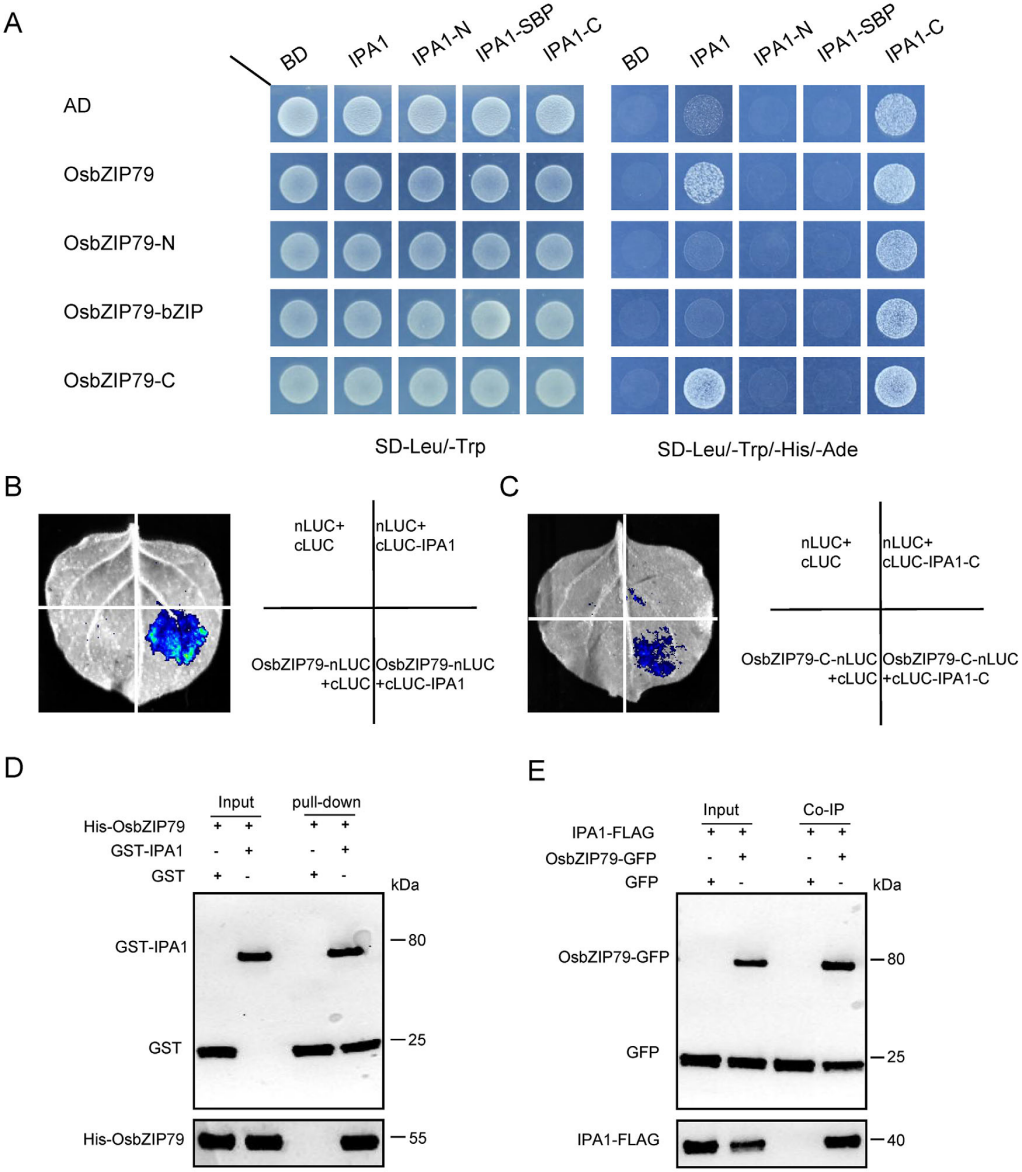
OsbZIP79 interacts with IPA1. (A, B) Yeast‐two‐hybrid (Y2H, A), luciferase complementation imaging (LCI, B) showing the interaction between IPA1 and OsbZIP79. (C) LCI assays showing the interaction between OsbZIP79‐C and IPA1‐C. (D, E) Pull‐down (D) and co‐immunoprecipitation (Co‐IP, E) assays showing the interaction between OsbZIP79 and IPA1. (D) Total proteins were immunoprecipitated with anti‐GFP agarose beads. Co‐immunoprecipitated IPA1‐FLAG was detected with an anti‐FLAG antibody.

The expression of *OsMPG1* in the *osbzip79‐1* mutant, OE line, and WT under salt stress conditions showed patterns similar to those observed for *OsHAK26* (Figure 4D). Also, the temporal patterns of salt‐induced *OsbZIP79* and *OsMPG1* transcription support the direct regulation of *OsMPG1* by OsbZIP79, similar to the observation of *OsHAK26* regulated by OsbZIP79 (Figure 4E). ChIP‐qPCR and DLR assays further confirmed the binding of OsbZIP79 to the *OsMPG1* promoter and its role in activating transcription (Figure 3E–G; Figures S7 and S8).

These findings collectively demonstrate that OsbZIP79 directly regulates key genes involved in both Na^+^/K^+^ balance and redox homeostasis, thereby contributing to enhanced salt tolerance in rice.

### 2.3 OsbZIP79 Physically Interacts with IPA1

To identify other factors that may be involved in the ‐mediated salt tolerance pathway, a yeast‐two‐hybrid (Y2H) screen was performed to identify OsbZIP79‐interacting proteins, which revealed IPA1 as a candidate (Figure 4A). To define the interaction interfaces, Y2H assays were conducted using full‐length and truncated IPA1 variants co‐expressed with OsbZIP79 fragments (OsbZIP79‐N, OsbZIP79‐bZIP, and OsbZIP79‐C; Figure S9). Results indicated that the C‐terminus of OsbZIP79 is the specific region required for its interaction with IPA1 (Figure 4A). We further validated the interaction between full‐length OsbZIP79 and IPA1 using LUC complementation (LCI) assays (Figure 4B). Given that the IPA1 C‐terminus exhibited autoactivation in the Y2H assay (Figure 4A), we utilized in vitro LCI assays to pinpoint the specific domains mediating this interaction. The results demonstrated a direct interaction between the C‐terminal fragments of both proteins (OsbZIP79‐C and IPA1‐C) (Figure 4C). To further corroborate this, we performed pull‐down and Co‐immunoprecipitation (Co‐IP) assays. The pull‐down assay revealed that His‐OsbZIP79 was successfully pulled down by GST‐IPA1, but not by GST alone (Figure 4C). Additionally, the Co‐IP assay demonstrated that IPA1‐FLAG could be co‐immunoprecipitated with OsbZIP79‐GFP, but not with GFP alone (Figure 4D). These findings strongly indicate a physical interaction between OsbZIP79 and IPA1.

### 2.4 IPA1 Attenuates OsbZIP79‐Mediated Transcriptional Activation of Downstream Target Genes

**FIGURE 5:**
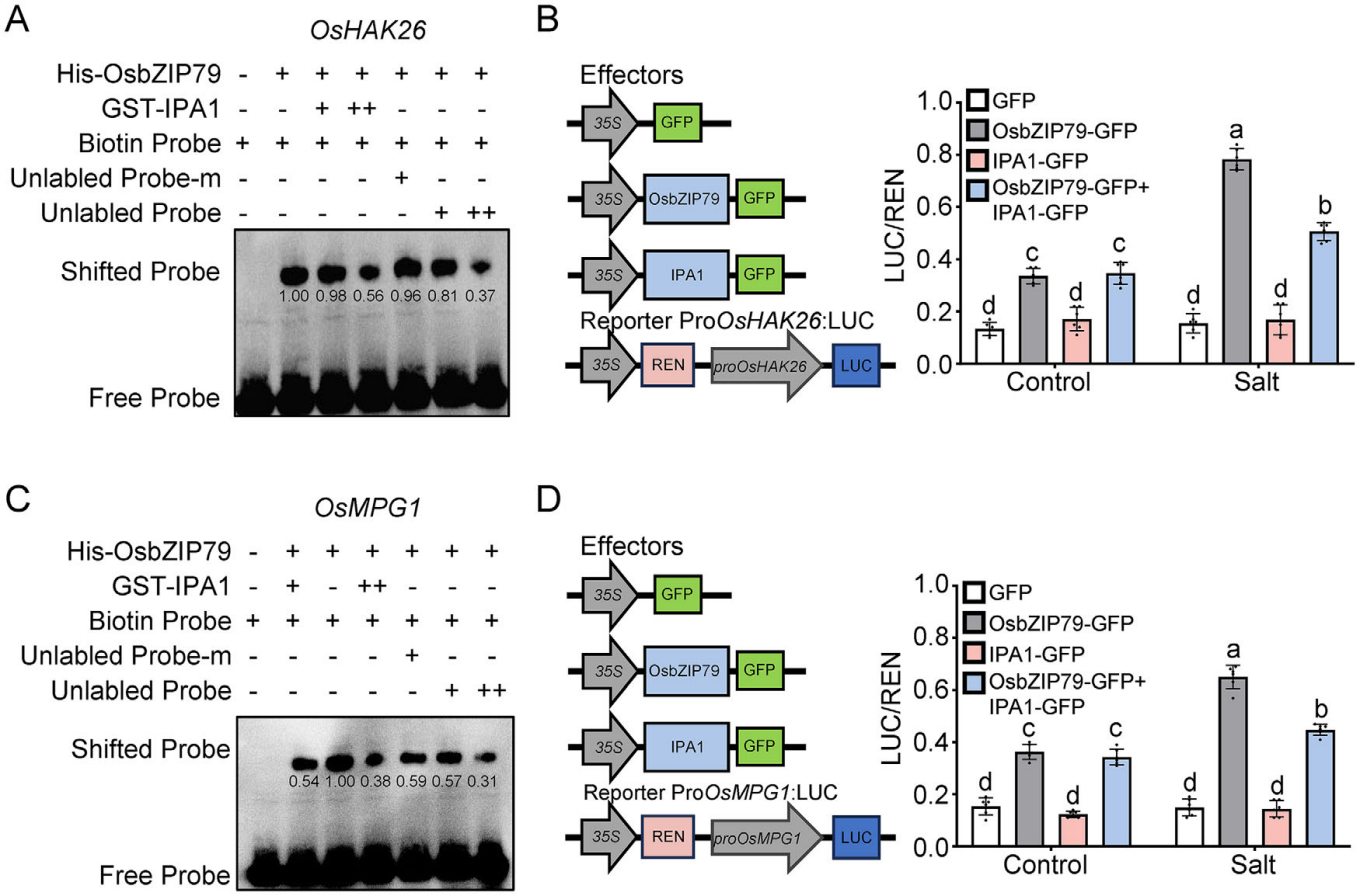
OsbZIP79 directly binds to and activates the *OsHAK26* and *OsMPG1* promoters. (A) EMSA assay showing OsbZIP79's binding affinity to the *OsHAK26* promoter. (B) OsbZIP79's activation effect on the *OsHAK26* promoter was evaluated by a DLR assay. The core constructs of the vectors are shown on the left. (C) EMSA assay showing OsbZIP79's binding affinity to the *OsMPG1* promoter. (D) OsbZIP79's activation effect on the *OsMPG1* promoter was evaluated by a DLR assay. The core constructs of the vectors are shown on the left. Values are means ± standard deviation (SD) (*n* = 6). Different letters indicate significant differences (*p* < 0.05).

To investigate the impact ofIPA1 on the activation of OsbZIP79‐regulated downstream genes, we performed electrophoretic mobility shift assay (EMSA) and DLR assays. In the EMSA assay, we observed that the C‐terminal region of OsbZIP79 directly binds to the *OsHAK26* promoter (Figure 5A), and the addition of GST‐IPA1 significantly reduced the intensity of the shifted probe bands, indicating that IPA1 significantly reduced the binding of OsbZIP79 to the *OsHAK26* promoter. DLR assays demonstrated that OsbZIP79 strongly enhanced luciferase activity driven by the *OsHAK26* promoter under both the control and salt stress conditions (Figure 5B), however, the co‐expression of IPA1 with OsbZIP79 markedly reduced this transcriptional activation under salt stress. Similarly, IPA1 significantly reduced the binding of OsbZIP79 to the *OsMPG1* promoter as revealed by the results of EMSA and DLR assays (Figure 5C,D). These results collectively indicate that the interaction of IPA1 and OsbZIP79 weakens OsbZIP79's capacity to bind to and activate its downstream target genes.

### 2.5 IPA1 Negatively Regulates OsbZIP79 Expression

**FIGURE 6:**
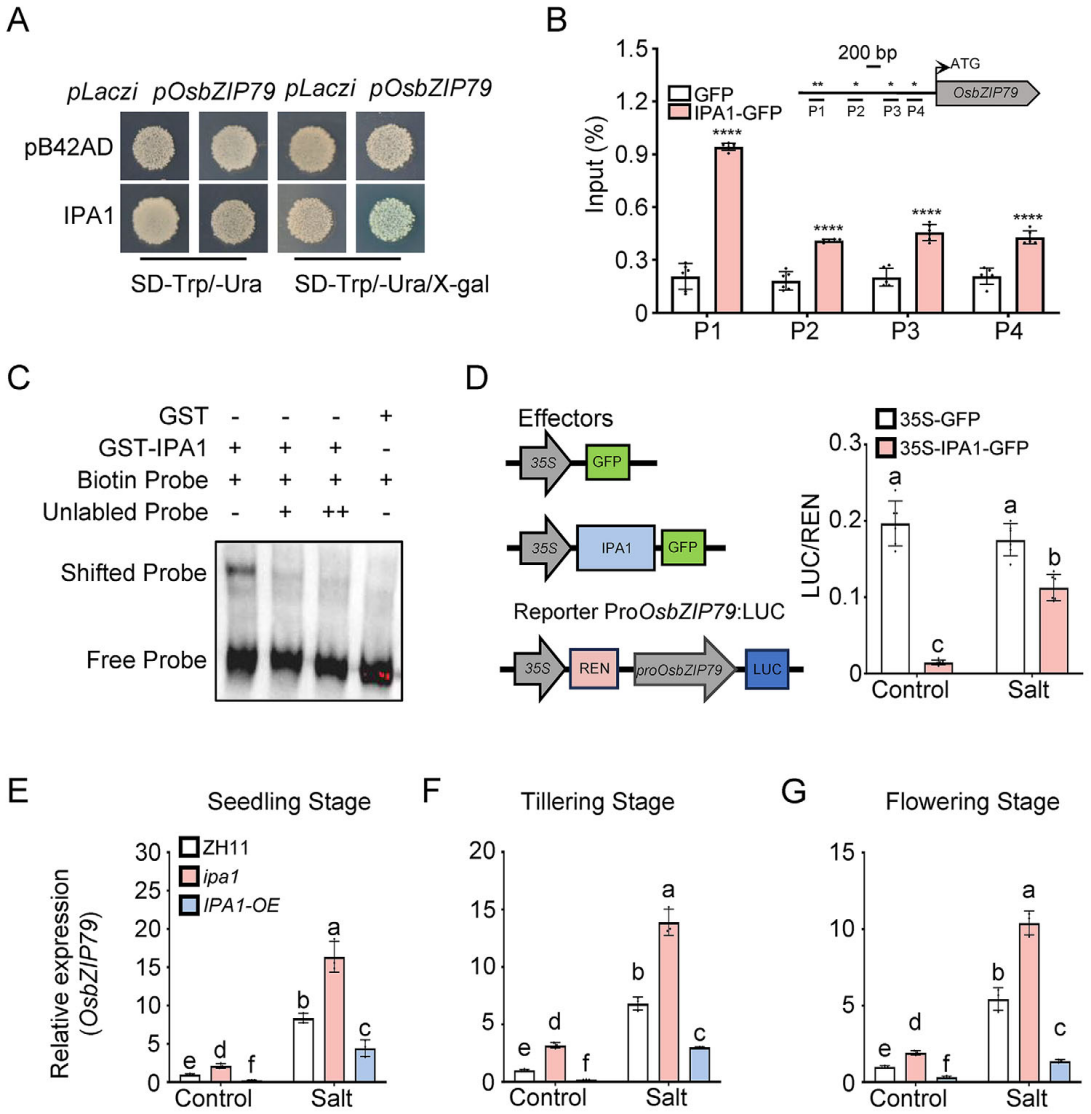
IPA1 negatively regulates *OsbZIP79* expression. (A) Y1H assay showing IPA1's binding affinity to the *OsbZIP79* promoter. (B) ChIP‐qPCR assay to detect the binding of IPA1 to the motifs in the *OsbZIP79* promoter. (C) EMSA assay showing IPA1's binding affinity to the *OsbZIP79* promoter. (D) IPA1's activation effect on the *OsbZIP79* promoter was evaluated by a DLR assay. The core constructs of the vectors are shown on the left. (E) *OsbZIP79* expression in ZH11, *ipa1*, and *IPA1‐OE* at the seeding, tillering, and flowering stages. Values are means ± SD (*n* = 3 in E, F, and G; *n* = 6 in B and D). Different letters represent significant differences (*P* < 0.05).

As a transcriptional factor, IPA1 is able to repress transcription of its target gene, e.g., *NAL11* [32]. To test whether IPA could also regulate the transcription of *OsbZIP79*, we examined the transcriptional control of *OsbZIP79* by IPA1 (Figure 6). Using a Y1H assay, we found that IPA1 directly binds to the promoter of *OsbZIP79* (Figure 6A). ChIP‐qPCR analysis showed significant enrichment of the P1, P2, P3, and P4 amplicons of the *OsbZIP79* promoter in the DNA immunoprecipitated with IPA1‐GFP compared with the GFP control (Figure 6B; Figure S10), and EMSA results also indicated that the IPA1 protein physically and directly binds to the *OsbZIP79* promoter (Figure 6C). DLR assays demonstrated that the presence of IPA1 substantially reduced *OsbZIP79* promoter‐driven luciferase activity under normal conditions, whereas this transcriptional repression was significantly attenuated under salt stress (Figure 6D), likely due to OsMPK4‐mediated IPA1 degradation in rice under salt stress [28], which reduces its repressive effect on the *OsbZIP79* promoter.

To confirm the repressive effect of IPA1 on *OsbZIP79* transcription, we further examined the expression levels of *OsbZIP79* in *IPA1* knockout (*ipa1*) and overexpression line (*IPA1‐OE*). In plants grown under both normal and salt‐stressed conditions, the *ipa1* mutant had *OsbZIP79* transcript levels significantly greater, and the *IPA1‐OE* had significantly lower levels then the WT, with similar results at both the tillering and flowering stages (Figure 6E‐G). This is likely because the overexpression of IPA1 results in a high protein level, so even though part of it is degraded under salt stress, the remaining residual IPA1 can still repress *OsbZIP79* expression.

The above results collectively demonstrate that IPA1 directly binds to the *OsbZIP79* promoter and represses its transcription under normal conditions, while this inhibitory effect is alleviated upon salt stress.

### 2.6 The OsbZIP79‐IPA1 Module Synergistically Regulates Na^+^/K^+^ and Redox Homeostasis to Confer Salt Tolerance in Rice

**FIGURE 7:**
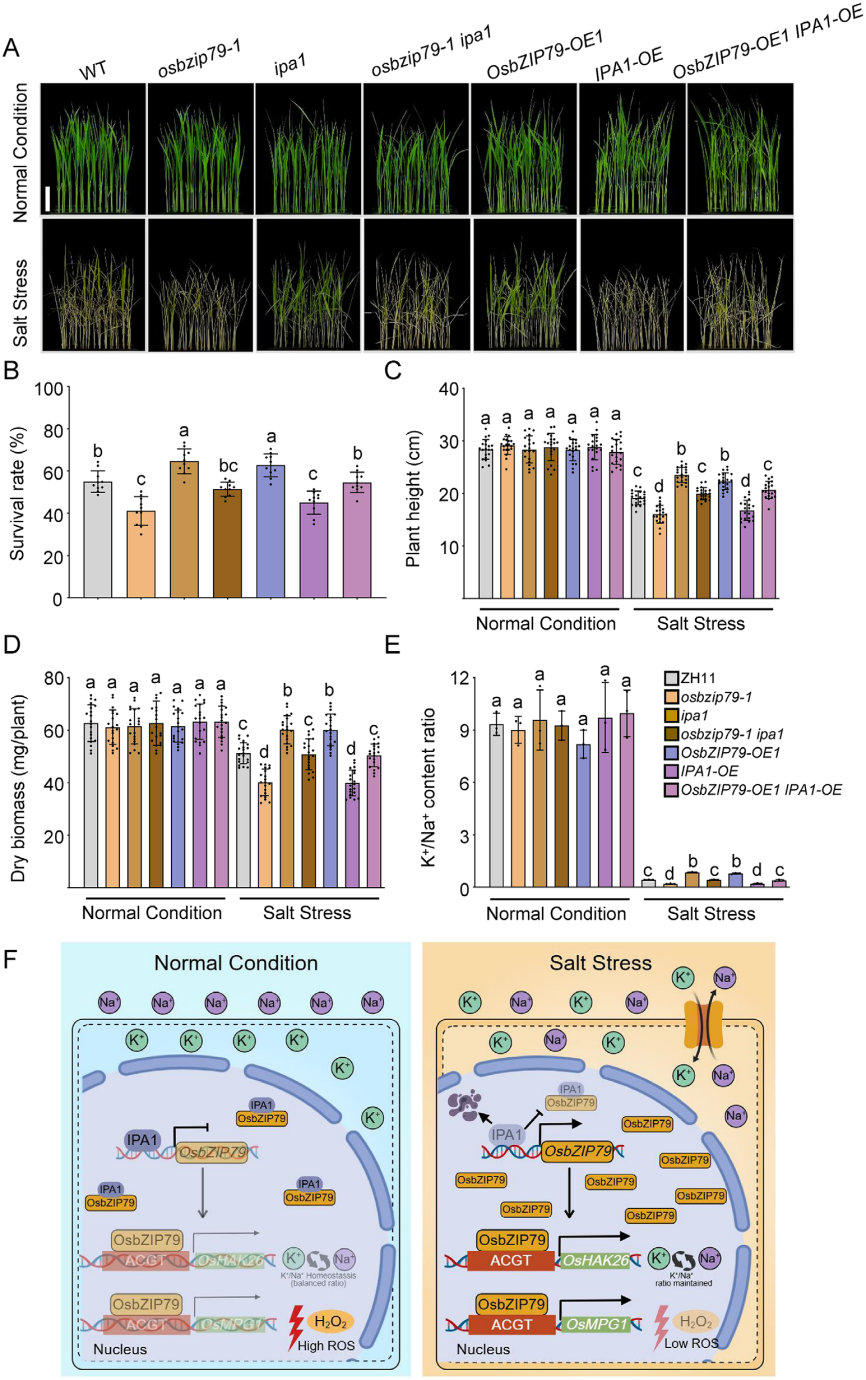
OsbZIP79‐IPA1 module regulates rice salt tolerance. (A–E) Phenotypes (A), survival rates (B), plant height (C), dry biomass (D), and shoot K^+^/Na^+^ content ratios (E) in ZH11, *osbzip79‐1*, *ipa1*, *osbzip79‐1 ipa1*, *OsbZIP79‐OE1*, *IPA1‐OE*, and *OsbZIP79‐OE1 IPA1‐OE*. Scale bar = 5 cm (A). Values are means ± standard deviation (SD) (*n* = 3 in B; *n* = 10 in E; *n* = 20 in C, D). Different letters indicate significant differences (*p* < 0.05). (F) A proposed model of OsbZIP79‐mediated rice salt tolerance.

To elucidate the genetic interplay between IPA1 and OsbZIP79 in regulating salt tolerance, we generated a double mutant (*osbzip79‐1 ipa1*) and a double overexpression (OE) line (*OsbZIP79‐OE1 IPA1‐OE*), alongside single mutants (*osbzip79‐1*, *ipa1* (Figure S11)) and single OE lines (*OsbZIP79‐OE1*, *IPA1‐OE*) (Figure 7A). Phenotypic analysis revealed that the double mutant *osbzip79‐1 ipa1* exhibited an intermediate salt tolerance phenotype relative to the single mutants: under salt stress, its survival rate (Figure 7B), plant height (Figure 7C), and dry biomass (Figure 7D) were significantly higher than the *ipa1* mutant but lower than the *osbzip79‐1* mutant. Similarly, the double OE line showed an intermediate phenotype between the single OE lines: its survival rate, plant height, and dry biomass under salt stress were significantly higher than the *OsbZIP79‐OE1* line but lower than the IPA1‐OE line.

Consistent with these phenotypes, the Na^+^/K^+^ ratio, redox homeostasis (e.g., ROS levels, antioxidant enzyme activities), and expression of *OsHAK26* and *OsMPG1* in the double mutant and double OE line were also intermediate between the single mutants/OE lines (Figures S12–S14). Overall, these data demonstrate that OsbZIP79 and IPA1 are key components of a shared regulatory network that integrates Na^+^/K^+^ homeostasis and redox balance to confer salt tolerance in rice, with their genetic interaction (additive effects in mutants/OE lines) supporting a cooperative rather than independent role in this process.

### 2.7 Identifying an Elite Haplotype of OsbZIP79 Contributing to Salt Tolerance in Rice

**FIGURE 8:**
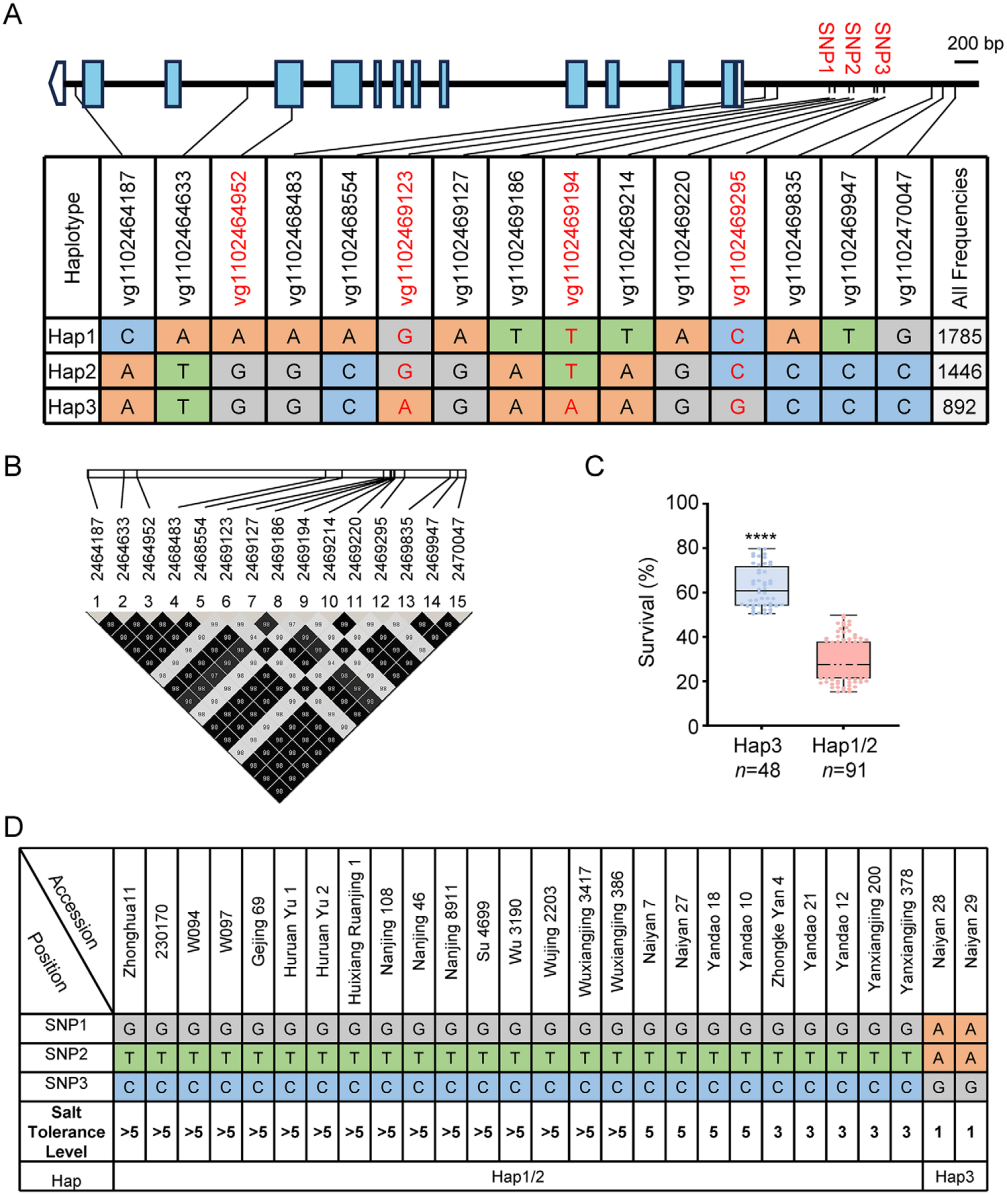
Analysis of elite OsbZIP79 haplotypes associated with salt tolerance in rice. (A) Gene structure and haplotype analysis of *OsbZIP79* identified in the coding sequence (CDS) and 2‐kb upstream region of the gene. (B) Pairwise values among all polymorphic sites in the *OsbZIP79* promoter and coding regions. (C) Survival rates of cultivars of various haplotypes (Hap1/2 and Hap3) under salt. Student's *t*‐test, *p* < 0.0001. (D) Comparisons of elite Hap1/2 and Hap3 distribution and survival rates under salt stress in the currently cultivated japonica and indica in China.

To investigate natural variations in *OsbZIP79* and their association with salt tolerance, we performed haplotype analysis of the *OsbZIP79* locus, including the 2‐kb upstream regulatory region and the full genomic sequence, using data from 4726 rice accessions available in RiceVarMap (Figure 8A,B; Figure S15A–C). Three major haplotypes were identified, with Hap1 predominantly present in *japonica* varieties, while Hap2 and Hap3 were mainly found in *indica* cultivars (Figure S15D,E). Phenotypic assessment of 262 accessions, which were selected from the initial 4726 accessions, revealed that Hap3 is strongly associated with enhanced salinity tolerance (Figure 8C; Table S2). Notably, expression analysis revealed that *OsbZIP79* transcript levels in Hap3 accessions are significantly upregulated under salt stress, whereas expression in Hap1 and Hap2 accessions remains largely unchanged (Figure S16A).

To further validate this association, we phenotypically evaluated a distinct set of 28 landrace germplasms (mostly conventional *japonica* varieties) that were independent of the 262‐accession subset. This panel included 26 accessions representing Hap1/Hap2 and two landrace accessions carrying Hap3. Our analysis demonstrated that materials harboring Hap3 exhibit significantly enhanced tolerance to salt stress compared with those carrying Hap1 or Hap2 (Figure 8D). Consistent with the population data, the two salt‐tolerant landraces harboring Hap3 exhibited a marked induction of *OsbZIP79* expression under salt stress, whereas the Hap1/Hap2 accessions showed no significant changes (Figure S19B). These findings support the conclusion that Hap3 likely represents a favorable haplotype that has been selected and retained through natural evolution and/or breeding programs for adaptation to saline environments.

## 3 Discussion

Soil salinity is a pervasive challenge that limits agricultural output worldwide, with rice, a key staple, being particularly susceptible to its detrimental effects on yield and quality. The intricate molecular pathways that enable rice to withstand salt stress are not yet fully elucidated. Our investigation has uncovered OsbZIP79, a previously uncharacterized bZIP transcription factor, which plays a pivotal role in enhancing salt tolerance in rice seedlings (Figure 1). Under normal conditions, IPA1 inhibits the gene expression of *OsbZIP79* (Figure 6) and competes for binding to the C‐terminus of the OsbZIP79 protein (Figure 4), thereby blocking its interaction with the promoters of downstream target genes (Figures 2 and 3). Under salt stress, IPA1 was phosphorylated at Thr180 by activated OsMPK4 and subsequently degraded [28]. Along with the degradation of IPA1, the gene expression of *OsbZIP79* is significantly increased under salt stress. Meanwhile, a large amount of OsbZIP79 protein can bind to the promoters of the target genes responsible for the homeostasis of Na^+^/K^+^ and redox regulation (Figures 2 and 3). This research elucidates a novel regulatory axis in the salt tolerance network involving the OsbZIP79‐IPA1 module (Figure 7F), suggesting potential avenues for enhancing rice salt tolerance through modern breeding strategies.

Basic leucine zipper (bZIP) transcription factors are a conserved family of proteins that play a crucial role in the regulation of gene expression in response to salt stress in plants [33]. These TFs are characterized by a highly conserved basic region and a leucine zipper dimerization domain, typically from 60 to 80 amino acids in length. In rice, several bZIP TFs are implicated in the transcriptional activation of stress‐responsive genes, particularly those involved in the abscisic acid (ABA)‐dependent signaling pathway. The overexpression of *OsbZIP71*, for instance, leads to the upregulation of genes encoding ion antiporters such as *OsCLC‐1*, *OsNHX1*, *OsHKT6*, and *OsVHA‐B*, as well as ROS scavenging enzymes like *OsCAT* [34]. Notably, OsbZIP71 directly binds to the promoter of *OsNHX1*, a gene responsible for the vacuolar compartmentalization of Na^+^ ions. Another bZIP TF, OsbZIP23, is essential for salt tolerance by upregulating genes responsive to osmotic stress, including dehydrins and LEA proteins [35]. OsHBP1b, a member of the bZIP TF family, enhances salt tolerance by activating genes involved in the antioxidant defense system [36]. Notably, *OsHBP1b* is located within the Saltol quantitative trait locus (QTL) region, underscoring its critical role in rice salt tolerance. Additionally, comparative transcript profiling indicates that *OsHBP1b* is highly expressed in the popular salt‐tolerant rice cultivar *Pokkali* [37]. In contrast, OsABI5 (OsbZIP10) functions as a negative regulator, altering the expression of numerous salt stress‐responsive genes. For example, OsABI5 significantly downregulates the expression of *OsHKT1;5*/*SKC1* while upregulating the *SalT* gene. Transcriptomic analysis reveals that many other bZIP TFs are also involved in rice's response to salt stress, although their specific regulatory functions require further functional studies. Overall, bZIP TFs primarily regulate salt tolerance through the pathways of active oxygen detoxification and ion homeostasis. In this context, identification of OsbZIP79 as a positive regulator of salt tolerance in rice reveals the complexity of molecular mechanisms governing plant salinity responses. Our data show the rapid induction of OsbZIP79 at both transcriptional and protein levels under salt stress, indicating its role as an early responder in the salt stress signaling pathway (Figure 1). This leads to upregulation of target genes (e.g., *OsHAK26* and *OsMPG1*) (Figures 2 and 3), suggesting direct transcriptional activation by OsbZIP79, crucial for cellular homeostasis under saline conditions. Consistent with this role, *osbzip79* mutants exhibit elevated Na^+^ levels, reduced K^+^ levels, and decreased antioxidant enzyme activities under salt stress (Figure 1; Figure S2), reflecting impaired oxidative stress management, leading to cellular damage. Conversely, *OsbZIP79* overexpression lines exhibit reduced H_2_O_2_ levels and enhanced antioxidant enzyme activities, suggesting improved ROS scavenging and cellular protection. Our findings also highlight OsbZIP79's dual role in coordinating salt stress tolerance by regulating both Na^+^/K^+^ balance and redox homeostasis, thereby effectively mitigating ion imbalance and oxidative stress.

Jia et al. [28] studied the gain‐of‐function mutant *ipa1‐3D* and the loss‐of‐function mutant *ipa1‐10*. The *ipa1‐10* mutant showed enhanced salt tolerance with a higher survival rate compared to WT, while *ipa1‐3D* exhibited opposite effects. Salt treatment increased Na^+^ accumulation in *ipa1‐3D* and decreased it in *ipa1‐10*, with minimal impact on K^+^ levels. They confirmed that IPA1 acts as a negative regulator of salt tolerance in rice. These findings suggest that IPA1 modulates Na^+^ influx under saline conditions, consistent with our results (Figure 7; Figure S12). Furthermore, the assays of Y2H, pull‐down, and Co‐IP confirmed a direct interaction between OsbZIP79 and IPA1, suggesting a complex regulatory mechanism (Figure 4). Interestingly, previous studies have demonstrated that OsMPK4 interacts with the SBP domain of IPA1 to phosphorylate Thr180, thereby promoting IPA1 degradation under salt stress [28]. In contrast, our LCI assays revealed that OsbZIP79 interacts with the C‐terminus of IPA1, rather than the N‐terminal region or the SBP domain (Figure 4C). These distinct binding regions suggest that OsbZIP79 and OsMPK4 do not compete for the same physical interface on the IPA1 protein. Consequently, the OsbZIP79‐IPA1 and OsMPK4‐IPA1 modules likely operate through independent, non‐competing mechanisms. Given their distinct binding domains, the upregulation of *OsbZIP79* under salt stress does not interfere with the OsMPK4‐mediated phosphorylation and subsequent degradation of IPA1. Mechanistically, as OsMPK4 promotes IPA1 degradation, the pool of IPA1 available to interact with OsbZIP79 diminishes. This allows the upregulated OsbZIP79 to be released from the OsbZIP79‐IPA1 complex, thereby enabling it to more abundantly regulate downstream salt‐responsive genes and ultimately enhance salt tolerance.

Transcriptome and DAP‐Seq analyses reveal numerous DEGs in the *osbzip79‐1* mutant, highlighting OsbZIP79's broad impact on the rice transcriptome under salt stress (Figures S3 and S4). The enrichment of stress response and Na^+^/K^+^ and redox homeostasis underscores its multifaceted role (Figures 2 and 3). Direct regulation of key target genes like *OsHAK26* and *OsMPG1* by OsbZIP79 was demonstrated by their downregulation in mutants and upregulation in overexpression lines under salt stress. Y1H, ChIP‐qPCR, and DLR assays provided evidence for direct binding of OsbZIP79 to these gene promoters, contributing to salt tolerance (Figures 2 and 3). Phenotypic analysis of double mutants and overexpression lines showed a synergistic relationship between OsbZIP79 and IPA1, with balanced regulation of Na^+^/K^+^ and redox homeostasis (Figure 7). The competitive interaction between the C‐terminus of OsbZIP79 and IPA1 for binding sites on target gene promoters adds complexity to the regulatory mechanism, serving as a molecular switch for precise and adaptive regulation of downstream genes, thereby enhancing plant's overall salt tolerance (Figures 2, 3, 4). These results showed that the OsbZIP79‐IPA1 module plays a crucial role in orchestrating Na^+^/K^+^ balance and redox homeostasis in salt‐stressed rice. Under normal conditions, IPA1 inhibits *OsbZIP79* expression and competes for binding to its C‐terminus, blocking interaction with target gene promoters. However, under salt stress, phosphorylated IPA1 by OsMPK4 was degraded, leading to a significant increase in *OsbZIP79* expression. This allows OsbZIP79 to bind to promoters of genes responsible for Na^+^/K^+^ and redox homeostasis, thereby enhancing cellular homeostasis and salt tolerance. This working model highlights *OsHAK26* and *OsMPG1* as primary direct executors of the OsbZIP79‐mediated salt response.

Beyond the molecular mechanism, our population genetic analysis provides insights into the natural variation and evolutionary history of *OsbZIP79*. Haplotype analysis identified Hap3 as an elite haplotype associated with enhanced salt tolerance. To elucidate the underlying basis of this advantage, we examined the sequence variations between Hap3 and other haplotypes. Interestingly, although the Hap3‐specific SNPs in the *OsbZIP79* promoter do not reside directly within the IPA1‐binding motif (Figure S10), DLR assays demonstrated that IPA1‐mediated transcriptional induction of the Hap3 promoter is significantly enhanced compared to other haplotypes (Figure S17). We postulate that these Hap3‐specific variations may alter the local chromatin architecture or create binding sites for unknown transcriptional co‐activators, thereby facilitating IPA1 recruitment or stabilizing the transcriptional complex under salt stress. Within the coding region, while most variations are synonymous, one missense mutation was identified. However, structural modeling indicates that this amino acid substitution does not perturb the overall protein conformation (Figure S18), suggesting that the functional superiority of Hap3 is primarily driven by transcriptional regulatory divergence rather than alterations in OsbZIP79 protein structure. To further substantiate the evolutionary significance of Hap3, we performed π ratio, Tajima D, and Fst analyses across 4726 rice accessions. The results reveal significant selection signals in the genomic region harboring Hap3 (Figure S19). This strong evidence indicates that Hap3 has been retained or subjected to artificial and natural selection during rice adaptation to saline environments, underscoring its pivotal role in environmental adaptation and breeding history.

In conclusion, our study uncovers a novel regulatory axis involving the OsbZIP79‐IPA1 complex that plays a pivotal role in enhancing salt tolerance in rice. The multifaceted role of OsbZIP79 in regulating Na^+^/K^+^ balance and redox homeostasis, coupled with its interaction with IPA1, provides a comprehensive framework for understanding the molecular mechanisms underlying salt stress response in rice. The identification of key target genes directly regulated by OsbZIP79 further elucidates the specific pathways through which this transcription factor mediates salt tolerance. These findings not only advance our knowledge of the genetic and molecular basis of salt tolerance in rice but also offer potential targets for genetic engineering and breeding strategies aimed at developing salt‐tolerant rice varieties. By leveraging the regulatory mechanisms identified in this study, future research can explore novel approaches to enhance the salt tolerance of rice, thereby contributing to sustainable agriculture in saline‐prone regions worldwide.

## 4 Materials and methods

### 4.1 Plant Materials

In our previous study [32], we developed knockout mutants for the rice gene *OsbZIP79*, namely *osbzip79‐1* and *osbzip79‐2*, as well as the overexpression (OE) lines, *OsbZIP79*‐*OE1* and *OsbZIP79*‐*OE2*, and the complementation lines, *osbzip79*‐*C1* and *osbzip79*‐*C2*. The *ipa1* mutant was generated using CRISPR/Cas9 technology, targeting the initial exon of *IPA1*. An overexpression line, *IPA1*‐*OE*, was constructed by inserting the IPA1 coding sequence into the *BsaI* site of the binary vector *pCAMBIA1301‐pUBI:GFP*. All transgenic rice plants were produced by transforming these plasmids into Zhonghua11 (ZH11) rice calli using *Agrobacterium*‐mediated transformation. The ZH11 cultivar served as the wild‐type (WT) control for these experiments.

### 4.2 Growth Conditions

Rice seeds were surface‐sterilized with 15% (v/v) NaClO and subsequently soaked in distilled water for 48 h at 28°C. The sterilized seeds were then transferred to 96‐well hydroponic trays containing half‐strength Murashige and Skoog (½ MS) medium. Seedlings were then cultivated in a growth chamber under a 16 h light/8 h dark photoperiod, with day/night temperatures of 30°C and 25°C, and approximately 70% relative humidity. In the hydroponic setup, two‐week‐old seedlings were introduced to a ½ MS nutrient solution supplemented with 150 mm NaCl for a seven‐day period. Three‐week‐old plants (two weeks of growth followed by one week of salt treatment) were subsequently harvested for physiological, molecular, and morphological analyses. For the soil salinity stress experiment, 4‐week‐old seedlings of the *osbzip79* mutant, wild‐type (WT; ZH11), and *OsbZIP79*‐overexpression (*OsbZIP79‐OE*) line were transplanted into 10 L pots filled with paddy soil with four seedlings per pot. Salt treatment was performed as described by Takagi et al. [38]: at the tillering stage, each pot received 2 L of 0.75% (w/w) NaCl solution once every 7 days for 4 consecutive weeks. After the salt treatment period, normal irrigation was resumed until maturity. Control plants were irrigated with an equal volume of fresh water. Agronomic traits were subsequently measured. Each treatment included 10 biological replicates.

### 4.3 Detection of Gene Expression Level

We employed previously described methods to assess relative gene expression levels [39, 40]. For this purpose, total RNA was isolated from rice tissues using the RNAprep Pure Plant Kit (Tiangen, Beijing, China) and subsequently reverse transcribed into first‐strand cDNA with the HiScript III 1st Strand cDNA Synthesis Kit (Vazyme, Nanjing, China). Quantitative real‐time PCR (qRT‐PCR) was conducted using the AceQ qPCR SYBR Green Master Mix (Vazyme, Nanjing, China). The specific primers used for qRT‐PCR are detailed in Table S1. The rice *OsACTIN* gene was employed as an internal control, as indicated in Table S1, and the expression data were calculated using the 2^−ΔΔCt^ method.

### 4.4 Assay of Na^+^ and K^+^ Concentrations

The Na^+^ and K^+^ concentrations in rice plants were quantified using a standard procedure as previously described [41]. Briefly, the samples were dried at 105°C for 2 h and further dried at 60°C for three days until a constant weight was achieved, after which dry biomass was recorded. Approximately 100 mg of the dried material was mixed with 5 mL of HNO_3_ and digested using a Multiwave system at 140°C for approximately 90 min. Finally, the concentrations of Na^+^ and K^+^ were quantified using an atomic absorption spectrometer, model AA‐7000 (Shimadzu, Tokyo, Japan).

### 4.5 Measurement of Hydrogen Peroxide (H_2_O_2_) Levels and Antioxidant Enzyme Activities

The concentration of H_2_O_2_ was quantified using the H_2_O_2_ assay kit (Solarbio, Beijing, China), based on the formation of a titanium‐peroxide complex with absorbance measured at 415 nm [42, 43]. Following previously established protocols [44, 45], the activities of the antioxidant enzymes superoxide dismutase (SOD), catalase (CAT), and glutathione S‐transferase (GST) were measured using commercial kits (Solarbio, Beijing, China). The assay for enzyme activity was standardized as follows: SOD activity was defined as the amount required to inhibit the photoreduction of 4‐nitro blue tetrazolium chloride (NBT) by 50% under light exposure; CAT activity was measured by the rate of H_2_O_2_ decomposition at 1 nm per minute; and GST activity was quantified by the conjugation of 1 µm of 1‐chloro‐2,4‐dinitrobenzene with reduced glutathione per minute.

### 4.6 Western Blot

For Western blot analysis, 200 mg of plant tissue was ground to a fine powder under liquid nitrogen. The powdered tissue was resuspended in 1 mL of extraction buffer [2% (w/v) SDS, 12% (w/v) sucrose, 50 mM dithiothreitol, 50 mm Na_2_CO_3_, 2 mm EDTA (pH 8.0), 0.1 mm phenylmethylsulfonyl fluoride, and a protease inhibitor cocktail (Sigma‑Aldrich, USA)] and incubated at 65°C for 1 h with gentle agitation. The protein concentration of the lysate was determined using the BCA Protein Assay kit (Thermo Scientific, USA). Following SDS‑PAGE separation, proteins were transferred to a PVDF membrane and subjected to immunoblotting. The membrane was probed with an anti‑GFP primary antibody at a dilution of 1:5,000 overnight at 4°C, followed by incubation with an appropriate HRP‑conjugated secondary antibody.

### 4.7 Transcriptome Sequencing (RNA‐Seq)

RNA‐Seq analysis was conducted on total RNA isolated from two‐week‐old seedlings after a 24‐h exposure to 150 mm NaCl salt stress, utilizing the TRIZOL reagent extraction method (Invitrogen, Waltham, MA, USA). The extracted RNA was used to prepare sequencing libraries, which were subsequently sequenced on the Illumina NovaSeq 6000 platform. To ensure robustness, the experiment was carried out with three independent biological replicates. The raw sequence data underwent quality filtering with Cutadapt (version 1.15). Processed reads were then mapped to the rice reference genome (MSU_v7.0) using HISAT2 (version 2.0.5), following the default alignment settings. Differentially expressed genes (DEGs) were identified using DEseq (version 1.30.0), applying a significance level of *p* < 0.05 and a fold change cutoff of |log_2_ FoldChange| > 1.0. Venn diagrams were created using the VENNY tool (version 2.1, available at https://bioinfogp.cnb.csic.es/tools/venny/).

### 4.8 DNA Affinity Purification Sequencing (DAP‐Seq)

The DAPseq data analysis was performed as described previously [46]. DAPseq reads were mapped to the *Oryza sativa* genome using BOWTIE2 [47]. To ensure the consistency of peak calls between two biological replicates (*p* < 0.05), we utilized MACS2 for peak calling and the irreproducible discovery rate (IDR) method for peak reproducibility assessment. For the identification of enriched motifs within the peak regions, we used MEME‐CHIP, and HOMER was used to annotate the bound peaks. Putative direct target genes were defined as those with binding peaks located within 2‐kilobases (2‐kb) upstream of the transcription start site. Gene Ontology (GO) enrichment analysis. GO enrichment analysis of potential OsbZIP79 target genes was performed using the DAVID Bioinformatics Resources (version 6.8; https://davidbioinformatics.nih.gov/). The gene list was uploaded and analyzed with the *Oryza sativa* genome as the background. Over‐representation of GO terms in the Biological Process, Molecular Function, and Cellular Component categories was assessed using a modified Fisher's exact test (EASE score). The resulting *P* values were corrected for multiple testing using the Benjamini–Hochberg method, and GO terms with an adjusted *P* value (false discovery rate) < 0.05 were considered significantly enriched.

### 4.9 Yeast‐One‐Hybrid (Y1H) Assay

The sequence of bait, situated roughly 2‐kb upstream of the transcription start site of the target genes, was amplified and cloned into the pLacZ vector. This construct was then used to transform the EGY48 yeast strain. The full‐length coding sequences of OsbZIP79 or IPA1 were inserted into the pB42AD vector to facilitate fusion with the bait in the yeast strain. The transformed yeast strains containing the positive bait were screened based on their ability to grow on SD‐Trp/‐Ura medium and incubated at 30°C for 3 days. Positive colonies were then transferred to plates with 5‐bromo‐4‐chloro‐3‐indolyl‐β‐D‐galactopyranoside to assess β‐galactosidase activity.

### 4.10 Chromatin Immunoprecipitation Quantitative PCR (ChIP‐qPCR) Analysis

Chromatin immunoprecipitation (ChIP) assays were performed as previously described [48, 49] with minor modifications. The CDS of *OsbZIP79* or *IPA1* was cloned into a pGreen‐35S vector using *E. coli* DH5α and then transformed into *A. tumefaciens* strain GV3101. For transient expression, four‐week‐old *N. benthamiana* leaves were infiltrated with agrobacterial suspensions (OD_600_ = 0.8) prepared in infiltration buffer (10 mm MES, 10 mm MgCl_2_, 150 µm acetosyringone, pH 5.6). After 48–72 h of incubation under light, total protein was extracted from infiltrated leaf tissues using RIPA buffer supplemented with protease inhibitors, followed by quantification via BCA assay. For ChIP, crosslinking was performed by vacuum infiltration of leaf tissues with 1% formaldehyde for 15 min, followed by quenching with 0.125 m glycine. Chromatin was sheared by sonication (30% amplitude) for 10 min (10 s ON and 30 s OFF) to yield 200–500 bp fragments. Immunoprecipitation was carried out overnight at 4°C with anti‐GFP antibody (5 µg per 100 µg chromatin) and Protein A/G magnetic beads. After stringent washes, precipitated DNA was reverse‐crosslinked (65°C, 4 h), purified by phenol‐chloroform extraction, and analyzed by qPCR using specific primers (Table S1) and SYBR Green Master Mix.

### 4.11 Dual‐Luciferase Reporter (DLR) Assay

The creation of effector plasmids involved cloning the CDS of *OsbZIP79* and *IPA1* into the pGreen‐35S‐GFP vector. In contrast, reporter plasmids were constructed by inserting promoter sequences into the pGreen II 0800‐LUC vector. Rice protoplasts were co‐transformed with these recombinant plasmids via PEG‐mediated transfection, followed by a 16‐h incubation. After the incubation, the expressed proteins were assessed for the LUC/REN activity ratio using a luminometer (TANON Chemiluminescent Imaging system), with measurements conducted using a commercial LUC assay kit (DL101‐01; Vazyme, Nanjing, China).

### 4.12 Yeast‐Two‐Hybrid (Y2H) Analysis

The Yeastmaker Yeast Transformation System 2 from Clontech was employed for Y2H assays, with all procedures meticulously followed according to the manufacturer´s instructions. The target coding sequences were amplified and cloned into the pGBKT7 (bait) and pGADT7 (prey) vectors. The constructed bait and prey plasmids were then co‐transformed into Y2HGold yeast cells. The transformed yeast cells were cultured and screened on selective media SD‐Trp/‐Leu (DDO) or SD‐Trp/‐Leu/‐His/‐Ade (QDO).

### 4.13 Luciferase Complementation Imaging (LCI) Analysis

The coding sequences were amplified and fused to either the N‐ or C‐terminus of luciferase, as previously described [50]. The resulting recombinant vectors were individually transformed into *Agrobacterium* strain. An equal volume of the bacterial suspension was then infiltrated into the leaves of 3‐week‐old *N. benthamiana* plants. Following a two‐day incubation period, LUC activity was visualized by adding 0.5 mm luciferin, and the luminescence signals were recorded using a TANON Chemiluminescent Imaging system.

### 4.14 In Vitro Pull‐Down Analysis

The full‐length coding sequence of *IPA1* was successfully cloned into the pDEST15 vector (Invitrogen, Waltham, MA, USA) using the Gateway cloning system, resulting in prokaryotic expression plasmids encoding GST‐tagged IPA1 (GST‐IPA1). This construct, as well as the empty pDEST15 vector, was individually transformed into *E*. *coli* Rosetta (DE3) cells (Novagen, Shanghai, China) for the production of recombinant GST‐fusion proteins. In parallel, the full‐length coding sequence of *OsbZIP79* was inserted into the pET30a vector (Novagen, Shanghai, China) to generate His‐tagged proteins (His‐OsbZIP79). For the pull‐down experiments, the GST fusion proteins were purified and coupled to MagneGST Glutathione Beads (Promega). Following this, the His‐tagged proteins were incubated with beads coated with either GST or GST‐IPA1. After washing with PBS buffer, the proteins retained on the beads were separated by SDS‐PAGE and detected by immunoblot analysis using an anti‐His antibody.

### 4.15 Co‐Immunoprecipitation (Co‐IP) Analysis

The Co‐IP assay was conducted as previously reported by Guo et al. [48] with some minor adjustments. The full‐length coding sequences (CDS) of *IPA1* were amplified and cloned into a vector tagged with FLAG, while the full‐length CDS of *OsbZIP79* was amplified and cloned into a vector tagged with GFP. The resulting constructs were then transformed into *A. tumefaciens* strain GV3101. For transient expression, different combinations of *A. tumefaciens* carrying the various constructs at an OD600 of 0.8 were co‐infiltrated into four‐week‐old *N. benthamiana* leaves. Following 36 h of infiltration, the co‐expressed proteins in the infiltrated leaves were extracted using Co‐IP buffer and analyzed by immunoblotting with anti‐FLAG and anti‐GFP antibodies. Extracts containing equal total protein were incubated with anti‐GFP Magnetic Beads (Chromotek) for 3 h with gentle rotation at 4°C. The beads were washed five times with Co‐IP buffer. The immunoprecipitated proteins were subsequently analyzed by immunoblotting with an anti‐FLAG antibody. Primers used for plasmid construction are listed in Table S1.

### 4.16 Electrophoretic Mobility Shift Assay (EMSA)

The EMSA was performed as previously described by Jiang et al. [32]. The purification of recombinant proteins was conducted as previously described [51]. The purified protein was incubated with various biotin‐labeled probes at 20°C for 20 min using a commercial Light Shift Chemiluminescent EMSA Kit (Promega). The probe details are provided in Table S1.

### 4.17 Haplotype Analysis

The single‐nucleotide polymorphism (SNP) data for 4726 cultivated rice (Oryza sativa) accessions were retrieved from the publicly available Rice Variation Map v2.0 database (RiceVarMap; http://ricevarmap.ncpgr.cn/). Haploview software was employed for pairwise linkage disequilibrium analysis.

### 4.18 Population Genetic Analysis

Nucleotide diversity (π), Tajima's D, and the fixation index (FST) were calculated to assess genetic differentiation and selection signatures around the *OsbZIP79* locus. All analyses were based on high‐quality biallelic SNPs from whole‐genome resequencing data. Nucleotide diversity (π) and Tajima's D were computed in sliding windows using VCFtools (v0.1.16) [52] with a window size of 100 kb and a step size of 10 kb. For visualization of the *OsbZIP79* genomic region, −log_10_(π) was plotted. The π ratio between two populations (e.g., population A vs. population B) was calculated as π_a_/π_6_. FST between populations was estimated according to the method of Weir and Cockerham [53] as implemented in VCFtools.

### 4.19 Evaluation of Salt Tolerance Grades Across the Whole Growth Period

Salt tolerance grades were assigned based on the method described in the Jiangsu Provincial Standard DB32/T 1845–2011 (2019 revision) at both the seedling and mature stages [54]. The seedling salt tolerance index (SSI) was calculated as the percentage reduction in total dry biomass under salt stress relative to the control: SSI (%) = [(control dry weight − salt‐stressed dry weight) / control dry weight] × 100. The mature‐stage salt tolerance index (MSI) was calculated similarly using grain yield: MSI (%) = [(control yield − salt‐stressed yield) / control yield] × 100. Each index was categorized into five grades: Grade 1 (extremely strong, 0–20%), Grade 3 (strong, 20–40%), Grade 5 (moderate, 40–60%), Grade 7 (weak, 60–80%), and Grade 9 (extremely weak, 80–100%). The final whole‐growth‐period salt tolerance grade of each rice accession was assigned as the inferior (higher numerical) grade between its SSI and MSI, reflecting the more sensitive stage under salinity stress.

### 4.20 Statistical Analysis

Data presented in the figures are expressed as mean values accompanied by standard deviation (SD), based on 20 replications for plant height and dry biomass, 10 replications for survival rate, 6 replications for ChIP‐qPCR and DLR assays, and 3 replications for all other measurements. To determine significant differences among multiple groups, one‐way or two‐way ANOVA was performed, followed by Tukey's post‐hoc tests. Groups with statistically significant differences at *p* < 0.05 are denoted by different letters. All statistical evaluations were conducted using GraphPad Prism 9.0 software.

## Author Contributions

M.J. and Q.S. conceived the study. H.W., B.L., C.H., CF.Z., Y.W., X.X., W.Y., Y.F., and G.S. carried out the experiments and performed data analysis. H.W., B.L., and M.J. finished the draft; P.G., CH.Z., Q.S., and M.J. edited and formed the final draft. All authors reviewed and approved the final manuscript.

## Funding

This study was supported by the Yangtze River Delta Science and Technology Innovation Community Joint Research (Basic Research) Project (2024CSJZN01100), the National Natural Science Foundation of China (32570362 and 32460077), the Hainan Provincial Natural Science Foundation of China (325RC799), the Hainan Province Science and Technology Special Fund (ZDYF2025XDNY093), the ‘Nanhai New Star’ Technology Innovation Talent Platform Project of Hainan Province (NHXXRCXM202362), and the Research Startup Funding from Hainan Institute of Zhejiang University (0201‐6602‐A12203).

## Conflicts of Interest

The authors declare no conflicts of interest.

## Supporting information




**Supporting File 1**: advs77806‐sup‐0001‐SuppMat.pdf.

**Supporting File 2**: advs77806‐sup‐0002‐SuppMat.xlsx.

## Data Availability

The data that support the findings of this study are available in the Supporting Information of this article.

## References

[1] L. Wang , Y. Wang , P. Yin , C. Jiang , and M. Zhang , “ZmHAK17 Encodes a Na^+^‐Selective Transporter That Promotes Maize Seed Germination Under Salt Conditions,” New Crops 1 (2024): 100024, 10.1016/j.ncrops.2024.100024.

[2] X. Liang , J. Li , Y. Yang , C. Jiang , and Y. Guo , “Designing Salt Stress‐Resilient Crops: Current Progress and Future Challenges,” Journal of Integrative Plant Biology 66, no. 3 (2024): 303–329, 10.1111/jipb.13599. 38108117

[3] S. A. Ganie , K. A. Molla , R. J. Henry , K. V. Bhat , and T. K. Mondal , “Advances in Understanding Salt Tolerance in Rice,” Theoretical and Applied Genetics 132, no. 4 (2019): 851–870, 10.1007/s00122-019-03301-8. 30759266

[4] S. A. Ganie , S. H. Wani , R. Henry , and G. Hensel , “Improving Rice Salt Tolerance by Precision Breeding in a New Era,” Current Opinion in Plant Biology 60 (2021): 101996, 10.1016/j.pbi.2020.101996. 33444976

[5] J. Yuan , H. Cao , W. Qin , et al., “Genomic and Modern Biotechnological Strategies for Enhancing Salt Tolerance in Crops,” New Crops 2 (2024): 100057, 10.1016/j.ncrops.2024.100057.

[6] H. Qin , Y. Li , and R. Huang , “Advances and Challenges in the Breeding of Salt‐tolerant Rice,” International Journal of Molecular Sciences 21, no. 21 (2020): 8385, 10.3390/ijms21218385. 33182265 PMC7664944

[7] Z. Ren , J. Gao , L. Li , et al., “A Rice Quantitative Trait Locus for Salt Tolerance Encodes a Sodium Transporter,” Nature Genetics 37, no. 10 (2005): 1141–1146, 10.1038/ng1643. 16155566

[8] X. Fan , H. Jiang , L. Meng , and J. Chen , “Gene Mapping, Cloning and Association Analysis for Salt Tolerance in Rice,” International Journal of Molecular Sciences 22, no. 21 (2021): 11674, 10.3390/ijms222111674. 34769104 PMC8583862

[9] J. Liu , G. Li , L. Chen , J. Gu , H. Wu , and Z. Li , “Cerium Oxide Nanoparticles Improve Cotton Salt Tolerance by Enabling Better Ability to Maintain Cytosolic K^+^/Na^+^ Ratio,” Journal of Nanobiotechnology 19, no. 1 (2021): 153, 10.1186/s12951-021-00892-7. 34034767 PMC8146236

[10] E. van Zelm , Y. Zhang , and C. Testerink , “Salt Tolerance Mechanisms of Plants,” Annual Review of Plant Biology 71 (2020): 403–433. 10.1146/annurev-arplant-050718-100005 32167791

[11] A. Li , Y. Yang , Y. Guo , et al., “ZmASR6 positively Regulates Salt Stress Tolerance in Maize,” New Crops 2 (2025): 100067, 10.1016/j.ncrops.2025.100067.

[12] A. Fukuda , A. Nakamura , A. Tagiri , et al., “Function, Intracellular Localization and the Importance in Salt Tolerance of a Vacuolar Na^+^/H^+^ Antiporter From Rice,” Plant and Cell Physiology 45, no. 2 (2004): 146–159, 10.1093/pcp/pch014. 14988485

[13] T. Obata , H. K. Kitamoto , A. Nakamura , A. Fukuda , and Y. Tanaka , “Rice Shaker Potassium Channel OsKAT1 Confers Tolerance to Salinity Stress on Yeast and Rice Cells,” Plant Physiology 144, no. 4 (2007): 1978–1985, 10.1104/pp.107.101154. 17586689 PMC1949902

[14] M. I. Uddin , Y. Qi , S. Yamada , et al., “Overexpression of a New Rice Vacuolar Antiporter Regulating Protein OsARP Improves Salt Tolerance in Tobacco,” Plant and Cell Physiology 49, no. 6 (2008): 880–890, 10.1093/pcp/pcn062. 18420595

[15] S. Liu , L. Zheng , Y. Xue , Q. Zhang , L. Wang , and H. Shou , “Overexpression of OsVP1 and OsNHX1 Increases Tolerance to Drought and Salinity in Rice,” Journal of Plant Biology 53, no. 6 (2010): 444–452, 10.1007/s12374-010-9135-6.

[16] T. Yang , S. Zhang , Y. Hu , et al., “The Role of a Potassium Transporter OsHAK5 in Potassium Acquisition and Transport From Roots to Shoots in Rice at Low Potassium Supply Levels,” Plant Physiology 166, no. 2 (2014): 945–959, 10.1104/pp.114.246520. 25157029 PMC4213120

[17] H. Wei , X. Wang , Y. He , H. Xu , and L. Wang , “Clock Component OsPRR73 Positively Regulates Rice Salt Tolerance by Modulating OsHKT2;1‐Mediated Sodium Homeostasis,” The EMBO Journal 40, no. 3 (2021): 105086, 10.15252/embj.2020105086. PMC7849171 33347628

[18] J. Zhang , J. Li , X. Wang , and J. Chen , “OVP1, a Vacuolar H^+^‐translocating Inorganic Pyrophosphatase (V‐PPase), Overexpression Improved Rice Cold Tolerance,” Plant Physiology and Biochemistry 49, no. 1 (2011): 33–38, 10.1016/j.plaphy.2010.09.014. 20974539

[19] X. Guo , Y. Wu , Y. Wang , Y. Chen , and C. Chu , “OsMSRA4.1 and OsMSRB1.1, Two Rice Plastidial Methionine Sulfoxide Reductases, Are Involved in Abiotic Stress Responses,” Planta 230, no. 1 (2009): 227–238, 10.1007/s00425-009-0934-2. 19415325

[20] S. Ouyang , S. He , P. Liu , W. Zhang , J. Zhang , and S. Chen , “The Role of Tocopherol Cyclase in Salt Stress Tolerance of Rice (Oryza sativa),” Science China Life Sciences 54, no. 2 (2011): 181–188, 10.1007/s11427-011-4138-1. 21318489

[21] Y.‐H. Choe , Y.‐S. Kim , I.‐S. Kim , et al., “Homologous Expression of γ‐glutamylcysteine Synthetase Increases Grain Yield and Tolerance of Transgenic Rice Plants to Environmental Stresses,” Journal of Plant Physiology 170, no. 6 (2013): 610–618, 10.1016/j.jplph.2012.12.002. 23294545

[22] F. K. Teixeira , L. Menezes‐Benavente , V. C. Galvao , R. Margis , and M. Margis‐Pinheiro , “Rice Ascorbate Peroxidase Gene family Encodes Functionally Diverse Isoforms Localized in Different Subcellular Compartments,” Planta 224, no. 2 (2006): 300–314, 10.1007/s00425-005-0214-8. 16397796

[23] C. Y. Hong , Y. T. Hsu , Y. C. Tsai , and C. H. Kao , “Expression of ASCORBATE PEROXIDASE 8 in Roots of Rice (Oryza sativa L.) Seedlings in Response to NaCl,” Journal of Experimental Botany 58, no. 12 (2007): 3273–3283, 10.1093/jxb/erm174. 17916638

[24] C. Y. Hong , Y. Y. Chao , M. Y. Yang , S. Y. Cheng , S. C. Cho , and C. H. Kao , “NaCl‐Induced Expression of Glutathione Reductase in Roots of Rice (Oryza sativa L.) Seedlings Is Mediated Through Hydrogen Peroxide but Not Abscisic Acid,” Plant and Soil 320, no. 1‐2 (2009): 103–115, 10.1007/s11104-008-9874-z.

[25] J. Duan and W. Cai , “OsLEA3‐2, an Abiotic Stress Induced Gene of Rice Plays a Key Role in Salt and Drought Tolerance,” PLoS ONE 7, no. 9 (2012): 45117, 10.1371/journal.pone.0045117. PMC3443202 23024799

[26] J. You , W. Zong , X. Li , et al., “The SNAC1‐Targeted Gene OsSRO1c Modulates Stomatal Closure and Oxidative Stress Tolerance by Regulating Hydrogen Peroxide in Rice,” Journal of Experimental Botany 64, no. 2 (2013): 569–583, 10.1093/jxb/ers349. 23202132 PMC3542048

[27] H. Wu , H. Ye , R. Yao , T. Zhang , and L. Xiong , “OsJAZ9 acts as a Transcriptional Regulator in Jasmonate Signaling and Modulates Salt Stress Tolerance in Rice,” Plant Science 232 (2015): 1–12, 10.1016/j.plantsci.2014.12.010. 25617318

[28] M. Jia , N. Luo , X. Meng , et al., “OsMPK4 Promotes Phosphorylation and Degradation of IPA1 in Response to Salt Stress to Confer Salt Tolerance in Rice,” Journal of Genetics and Genomics 49, no. 8 (2022): 766–775, 10.1016/j.jgg.2022.06.009. 35803541

[29] Y. Li , J. Zhou , Z. Li , et al., “SALT and ABA RESPONSE ERF1 Improves Seed Germination and Salt Tolerance by Repressing ABA Signaling in Rice,” Plant Physiology 189, no. 2 (2022): 1110–1127, 10.1093/plphys/kiac125. 35294556 PMC9157093

[30] B. Wang , B. Xu , Y. Liu , et al., “A Novel Mechanism of the Signaling Cascade Associated with the SAPK10‐bZIP20‐NHX1 Synergistic Interaction to Enhance Tolerance of Plant to Abiotic Stress in Rice,” Plant Science 323 (2022): 111393. 35878697 10.1016/j.plantsci.2022.111393

[31] J. Yu , C. Zhu , W. Xuan , et al., “Genome‐Wide Association Studies Identify OsWRKY53 as a Key Regulator of Salt Tolerance in Rice,” Nature Communications 14, no. 1 (2023): 3550, 10.1038/s41467-023-39167-0. PMC10272163 37321989

[32] M. Jiang , Y. Song , R. Yang , et al., “Melatonin Activates the OsbZIP79–OsABI5 Module That Orchestrates Nitrogen and ROS Homeostasis to Alleviate Nitrogen‐Limitation Stress in Rice,” Plant Communications 4, no. 6 (2023): 100674, 10.1016/j.xplc.2023.100674. 37598294 PMC10721462

[33] K. S. Ponce , L. Meng , L. Guo , Y. Leng , and G. Ye , “Advances in Sensing, Response and Regulation Mechanism of Salt Tolerance in Rice,” International Journal of Molecular Sciences 22, no. 5 (2021): 2254, 10.3390/ijms22052254. 33668247 PMC7956267

[34] C. Liu , B. Mao , S. Ou , et al., “OsbZIP71, a bZIP Transcription Factor, Confers Salinity and Drought Tolerance in Rice,” Plant Molecular Biology 84, no. 1‐2 (2014): 19–36, 10.1007/s11103-013-0115-3. 23918260

[35] Y. Xiang , N. Tang , H. Du , H. Ye , and L. Xiong , “Characterization of OsbZIP23 as a Key Player of the Basic Leucine Zipper Transcription Factor family for Conferring Abscisic Acid Sensitivity and Salinity and Drought Tolerance in Rice,” Plant Physiology 148, no. 4 (2008): 1938–1952, 10.1104/pp.108.128199. 18931143 PMC2593664

[36] P. Das , N. Lakra , K. Nutan , S. L. Singla‐Pareek , and A. Pareek , “A Unique bZIP Transcription Factor Imparting Multiple Stress Tolerance in Rice,” Rice 12, no. 1 (2019): 58, 10.1186/s12284-019-0316-8. 31375941 PMC6890918

[37] N. Lakra , K. Nutan , P. Das , K. Anwar , S. L. Singla‐Pareek , and A. Pareek , “A Nuclear‐localized Histone‐gene Binding Protein From Rice (OsHBP1b) Functions in Salinity and Drought Stress Tolerance by Maintaining Chlorophyll Content and Improving the Antioxidant Machinery,” Journal of Plant Physiology 176 (2015): 36–46, 10.1016/j.jplph.2014.11.005. 25543954

[38] H. Takagi , M. Tamiru , A. Abe , et al., “MutMap Accelerates Breeding of a Salt‐tolerant Rice Cultivar,” Nature Biotechnology 33, no. 5 (2015): 445–449, 10.1038/nbt.3188. 25798936

[39] M. Jiang , X. Wu , Y. Song , H. Shen , and H. Cui , “Effects of OsMSH6 Mutations on Microsatellite Stability and Homeologous Recombination in Rice,” Frontiers in Plant Science 11 (2020): 220, 10.3389/fpls.2020.00220. 32194600 PMC7062918

[40] M. Jiang , B. Wang , R. Ye , et al., “Evidence and Impacts of Nanoplastic Accumulation on Crop Grains,” Advanced Science 9, no. 33: 2202336, 10.1002/advs.202202336. PMC9685458 36251925

[41] J. Qian , R. Shan , Y. Shi , et al., “Zinc Oxide Nanoparticles Alleviate Salt Stress in Cotton (Gossypium hirsutum L.) by Adjusting Na^+^/K^+^ Ratio and Antioxidative Ability,” Life 14, no. 5 (2024): 595, 10.3390/life14050595. 38792616 PMC11121869

[42] R. Li , M. Jiang , J. Huang , I. M. Møller , and Q. Shu , “Mutations of the Genomes Uncoupled 4 Gene Cause ROS Accumulation and Repress Expression of Peroxidase Genes in Rice,” Frontiers in Plant Science 12 (2021): 682453, 10.3389/fpls.2021.682453. 34178000 PMC8232891

[43] Y. Song , C. Zheng , S. Li , J. Chen , and M. Jiang , “Chitosan‐magnesium Oxide Nanoparticles Improve Salinity Tolerance in Rice,” ACS Applied Materials & Interfaces 15 (2023): 20649–20660. 37078774 10.1021/acsami.3c00043

[44] Y. Song , C. Zheng , B. Rasbin , S. Li , J. Chen , and M. Jiang , “Astaxanthin Synthesized Gold Nanoparticles Enhance Salt Stress Tolerance in Rice by Enhancing Tetrapyrrole Biosynthesis and Scavenging Reactive Oxygen Species in Vitro,” Plant Stress 6 (2022): 100122, 10.1016/j.stress.2022.100122.

[45] N. Xu , Y. Song , C. Zheng , S. Li , Z. Yang , and M. Jiang , “Indole‐3‐acetic Acid and Zinc Synergistically Mitigate Positively Charged Nanoplastic‐induced Damage in Rice,” Journal of Hazardous Materials 455 (2023): 131637, 10.1016/j.jhazmat.2023.131637. 37210880

[46] R. Li , Y. Song , X. Wang , et al., “OsNAC5 Orchestrates OsABI5 to Fine‐Tune Cold Tolerance in Rice,” Journal of Integrative Plant Biology 66, no. 4 (2024): 660–682, 10.1111/jipb.13585. 37968901

[47] B. Langmead and S. L. Salzberg , “Fast Gapped‐Read Alignment with Bowtie 2,” Nature Methods 9, no. 4 (2012): 357–359, 10.1038/nmeth.1923. 22388286 PMC3322381

[48] M. Guo , F. Yang , L. Zhu , et al., “Loss of Cold Tolerance Is Conferred by Absence of the WRKY34 Promoter Fragment During Tomato Evolution,” Nature Communications 15, no. 1 (2024): 6667, 10.1038/s41467-024-51036-y. PMC11303406 39107290

[49] M. Jiang , H. Zhang , Y. Song , et al., “Transcription Factor OsbZIP10 Modulates Rice Grain Quality by Regulating OsGIF1,” The Plant Journal 119, no. 5 (2024): 2181–2198, 10.1111/tpj.16911. 38981001

[50] M. Jiang , S. Dai , Y. Zheng , et al., “An Alanine to Valine Mutation of Glutamyl‐tRNA Reductase Enhances 5‐aminolevulinic Acid Synthesis in Rice,” Theoretical and Applied Genetics 135, no. 8: 2817–2831, 10.1007/s00122-022-04151-7. 35779128

[51] R. Li , C. Zheng , B. Wang , et al., “Monoculm1 Confers Cold Tolerance at the Seedling Stage in Rice,” Molecular Plant 19 (2026): 81–99, 10.1016/j.molp.2025.10.016. 41139960

[52] P. Danecek , A. Auton , G. Abecasis , et al., “The Variant Call Format and VCFtools,” Bioinformatics 27, no. 15 (2011): 2156–2158, 10.1093/bioinformatics/btr330. 21653522 PMC3137218

[53] B. S. Weir and C. C. Cockerham , “Estimating F‐statistics for the Analysis of Population Structure,” Evolution; International Journal of Organic Evolution 38 (1984): 1358–1370. 28563791 10.1111/j.1558-5646.1984.tb05657.x

[54] DB32/T 1845–2011. Technical Regulation for Identification on Salinity Tolerance of Rice in Whole Growth Period. *Jiangsu Provincial Bureau of Market Regulation*, China. (2019 revision).

