# Supplementary material for: Dual Repression by IPA1 Fine‐Tunes OsbZIP79‐Mediated Salt Tolerance in Rice
Source: Adv Sci (Weinh). 2026 Sep 16:e77806. Online ahead of print. doi: 10.1002/advs.77806 (PMC13583093; doi:10.1002/advs.77806)
Supplement: Supplementary file 1 [file ADVS-9999-e77806-s002.pdf]

Supporting Information

Figure S1. Salt stress induces the gene expression of *OsbZIP79*. (A) Expression level of *OsbZIP79* under salt stress in rice leaves. 14-day-old ZH11 seedlings were transferred to salt stress or normal conditions for indicated times. ** indicates $P < 0.01$. (B) Expression levels of *OsbZIP79* in various tissues exposed to salt stress. Values were reported relative to 0 h, which were assigned as 1.0. Values were shown as means ± standard deviation (SD) ($n = 3$).

Figure S2. The ROS scavenging index in ZH11, *osbzip79*, *OsbZIP79-OE*, and *osbzip79-C* seedlings under salt stress. (A-D) The content of hydrogen peroxide (H_2O_2, A) and activities of superoxide dismutase (SOD, B), catalase (CAT, C), and glutathione S-transferase (GST, D) were measured. Values are means ± standard deviation (SD) ($n = 3$). Different letters represent significant differences ($P < 0.05$).

Figure S3. Transcriptome analysis of ZH11 and *osbzip79-1* under salt stress. (A) Heatmaps of differentially expressed genes (DEGs) in ZH11 and *osbzip79-1*. (B) Volcano plots of total genes expression profiles in *osbzip79-1* as compared to ZH11. DEGs were chosen according to ∣log2 Fold Change ∣>1.5 and padj < 0.05.

Figure S4. Identification and analysis of OsbZIP79 binding sites by DNA affinity purification sequencing analysis. (A) Distribution of OsbZIP79 binding sites corresponding to the -2,000 to +2,000 nucleotides around the transcription start site (TSS). (B) Distribution of OsbZIP79 binding sites in different rice chromosomes. (C) Distribution of OsbZIP79 binding sites in different regions of the rice genome. (D) The OsbZIP79-binding motif identified in the 2-kbp flanking sequences around the genic peaks. (E) Gene Ontology (GO) enrichment of potential OsbZIP79 target genes.

Figure S5. Identification of OsbZIP79-regulated downstream genes involved in sodium-potassium transport. (A) Heatmap of *LOC_Os08g39950* and *LOC_Os01g20160*. (B) IGV tracks of DAP-seq peaks at the *LOC_Os08g39950* and *LOC_Os01g20160* loci. Rep 1 and Rep 2 denote two independent replicates. Numbers in brackets indicate the peak height range ([0–100] and [0–50]). (C) Y1H assay testing the binding of OsbZIP79 to the promoter of *LOC_Os08g39950* and *LOC_Os01g20160*. (D) OsbZIP79's activation effect on *LOC_Os01g20160* promoter was evaluated by dual luciferase reporter (DLR) assay. The core constructs of the vectors are shown on the left. Values are means ± SD ($n = 6$). Different letters represent significant differences ($P < 0.05$).

Figure S6. Schematic diagram and nucleotide sequence of the *OsHAK26* promoter. (A) Schematic diagram of the *OsHAK26* promoter. (B) Nucleotide sequence of the *OsHAK26* promoter. The predicted OsbZIP79 binding motif ACGT is highlighted in yellow background.

Figure S7. Identification of OsbZIP79-regulated downstream genes involved in ROS scavenging-related. (A) Heatmap of *LOC_Os01g62840, LOC_Os01g10110*, and *LOC_Os04g09920*. (B) IGV tracks of DAP-seq peaks at the *LOC_Os01g62840, LOC_Os01g10110*, and *LOC_Os04g09920* loci. Rep 1 and Rep 2 denote two independent replicates. Numbers in brackets indicate the peak height range ([0–100], [0–200] and [0–50]). (C) Y1H assay testing the binding of OsbZIP79 to the promoter of *LOC_Os01g62840, LOC_Os01g10110*, and *LOC_Os04g09920*. (D) OsbZIP79's activation effect on *LOC_Os01g10110* and *LOC_Os04g09920* promoter was evaluated by dual luciferase reporter (DLR) assay. The core constructs of the vectors are shown on the left. Values are means ± SD ($n = 6$). Different letters represent significant differences ($P < 0.05$).

Figure S8. Schematic diagram and nucleotide sequence of the *OsMPG1* promoter. (A) Schematic diagram of the *OsMPG1* promoter. (B) Nucleotide sequence of the *OsMPG1* promoter. The predicted OsbZIP79 binding motif ACGT is highlighted in yellow background.

Figure S9. A schematic diagram of OsbZIP79 and IPA1.

Figure S10. Schematic diagram and nucleotide sequence of the *OsbZIP79* promoter. (A) Schematic diagram of the *OsbZIP79* promoter. (B) Nucleotide sequence of the *OsbZIP79* promoter. The predicted IPA1 binding motif GTAC is highlighted in yellow background.

Figure S11. Schematic diagram of IPA1 for CRISPR/Cas9-mediated mutagenesis. (A) Schematic diagram of *IPA1* indicating the target sites of CRIPSR-Cas9-mediated mutagenesis in ZH11 background. (B) The two-dimensional structures of IPA1 and its mutant were analyzed on SWISSMODEL (https://www.swissmodel.expasy.org/). (C) The amino acid sequences of IPA1 mutant proteins were aligned to that of the wild-type protein using the Clustal Omega Multiple Sequence Alignment (https://www.ebi.ac.uk/Tools/msa/clustalo/).

Figure S12. Na^+ and K^+ contents in ZH11, *osbzip79-1*, *ipa1*, *osbzip79-1 ipa1*, *OsbZIP79-OE1*, *IPA1-OE*, and *OsbZIP79-OE1 IPA1-OE*. (A-B) Shoot Na^+ (A) and K^+ (B) contents were measured. Values are means ± SD ($n = 3$). Different letters indicate significant differences ($P < 0.05$).

Figure S13. The ROS scavenging index in ZH11, *osbzip79-1*, *ipa1*, *osbzip79-1 ipa1*, *OsbZIP79-OE1*, *IPA1-OE*, and *OsbZIP79-OE1 IPA1-OE* seedlings. (A-D) The content of hydrogen peroxide (H_2O_2, A) and activities of superoxide dismutase (SOD, B), catalase (CAT, C), and glutathione S-transferase (GST, D) were measured. Valuesare means ± standard deviation (SD) ($n = 3$). Different letters indicate significant differences ($P < 0.05$).

Figure S14. The gene expression in ZH11, *osbzip79-1*, *ipa1*, *osbzip79-1 ipa1*, *OsbZIP79-OE1*, *IPA1-OE*, and *OsbZIP79-OE1 IPA1-OE* seedlings. (A) *OsHAK26* and (B) *OsMPG1* gene expression were measured. Values are means ± standard deviation (SD) ($n = 3$). Different letters indicate significant differences ($P < 0.05$).

Figure S15. Nucleotide polymorphism for coding and promoter regions of OsbZIP79. (A) All polymorphic sites (Minor Allele Frequency [MAF]) > 5% on the *OsbZIP79* promoter and coding regoins. (B) r^2 values between tag SNPs and linked alleles. (C) Haplotype analysis of the *OsbZIP79* promoter and coding regoins. (D) Phylogenetic relationship rice germplasm based on the sequence of *OsbZIP79* promoter and coding regoins. (E) Haplotype analysis of *OsbZIP79* identified in the coding sequence (CDS) and 2-kb region upstream region of the gene.

Figure S16. Expression of *OsbZIP79* in different haplotypes and major rice cultivars. (A) Expression of *OsbZIP79* in different haplotypes. (B) Expression of *OsbZIP79* in major rice cultivars. Values are means ± standard deviation (SD) ($n = 6$). Different letters indicate significant differences ($P < 0.05$).

Figure S17. The function of IPA1 on the transcriptional activity of *OsbZIP79*'s different haplotypes measured by dual-luciferase (LUC/REN) reporter assay.

Figure S18. Functional characterization of the vg1102464952 coding variant. (A) Amino acid sequence alignment flanking the vg1102464952 coding variant before and after mutation. (B) Protein structure analysis of wild-type and mutant alleles at the vg1102464952 coding variant. Values are means ± standard deviation (SD) ($n = 6$). Different letters indicate significant differences ($P < 0.05$).

Figure S19. Population genetic statistics for the OsbZIP79 genomic region. (A) $-\log_{10}(\pi)$, where π represents nucleotide diversity. (B) Nucleotide diversity ratio (π ratio). (C) Tajima's D. (D) Fixation index (FST).

Figure S1

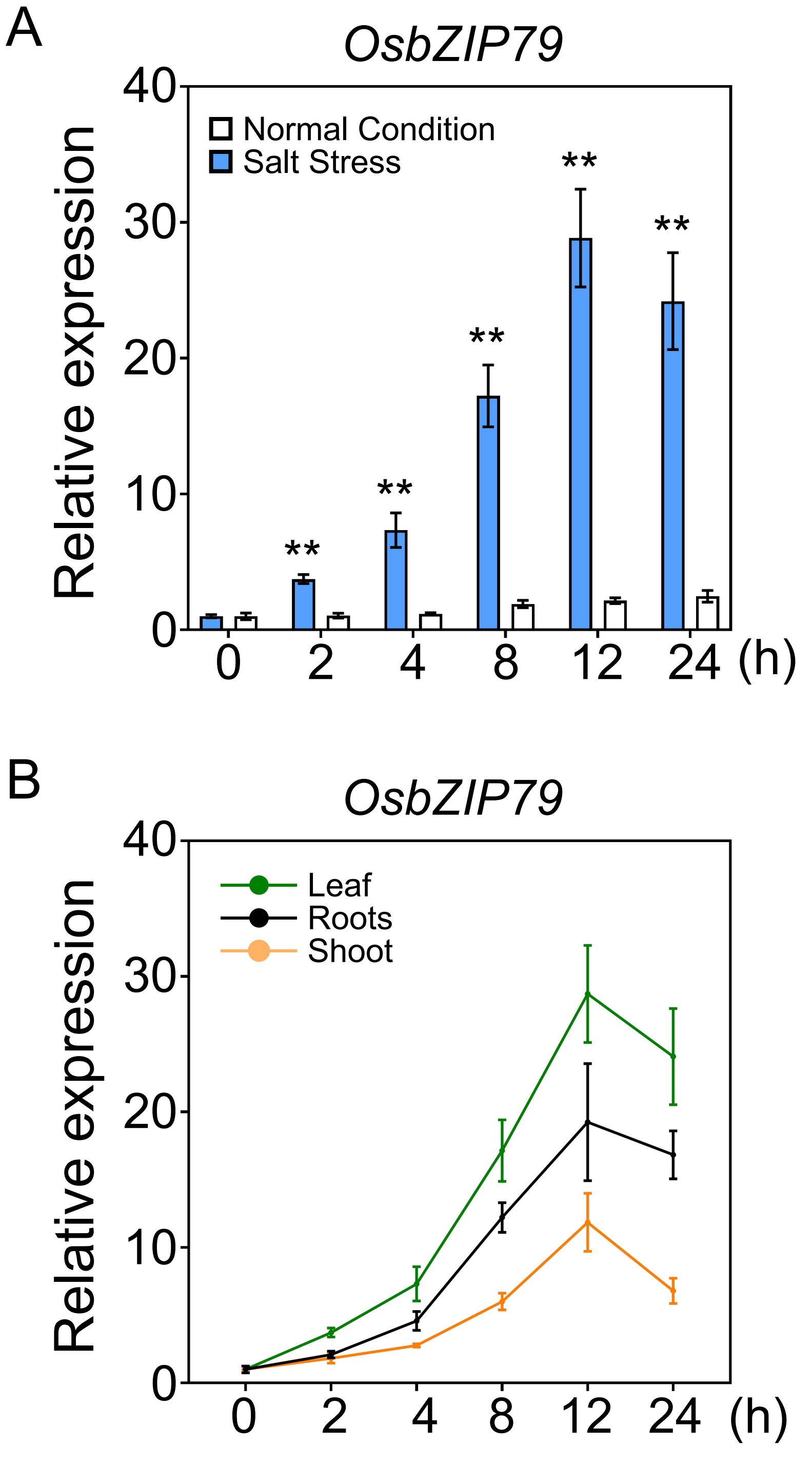
A
OsbZIP79
Relative expression
40
30
20
10
0
Normal Condition
Salt Stress
**
**
**
**
**
0
2
4
8
12
24
(h)
B
OsbZIP79
Relative expression
40
30
20
10
0
Leaf
Roots
Shoot
0
2
4
8
12
24
(h)

Figure S2

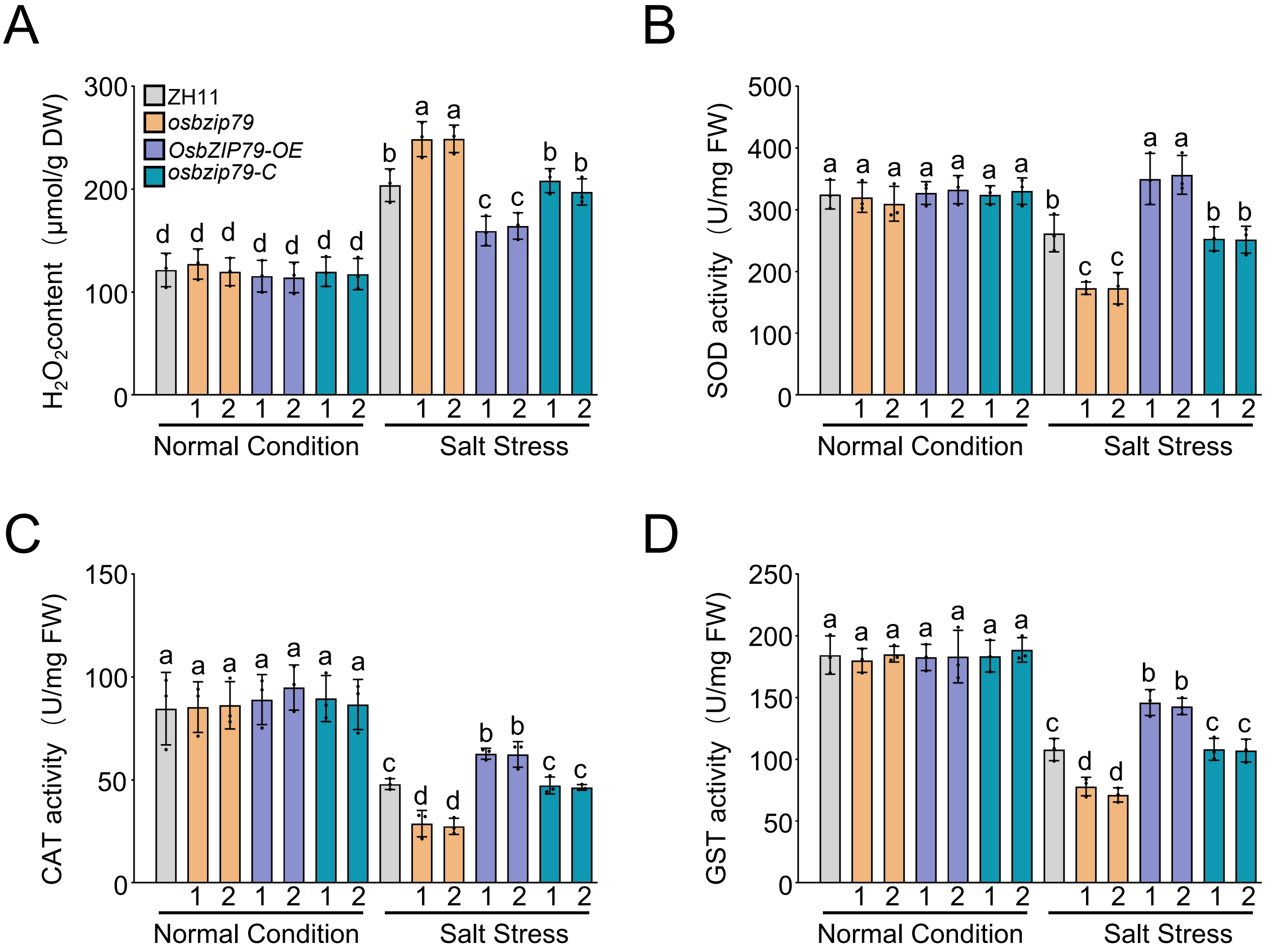

A
B
C
D
ZH11
osbzip79
OsbZIP79-OE
osbzip79-C
H2O2content（μmol/g DW)
SOD activity（U/mg FW)
CAT activity（U/mg FW)
GST activity（U/mg FW)
Normal Condition
Salt Stress

Figure S3

A

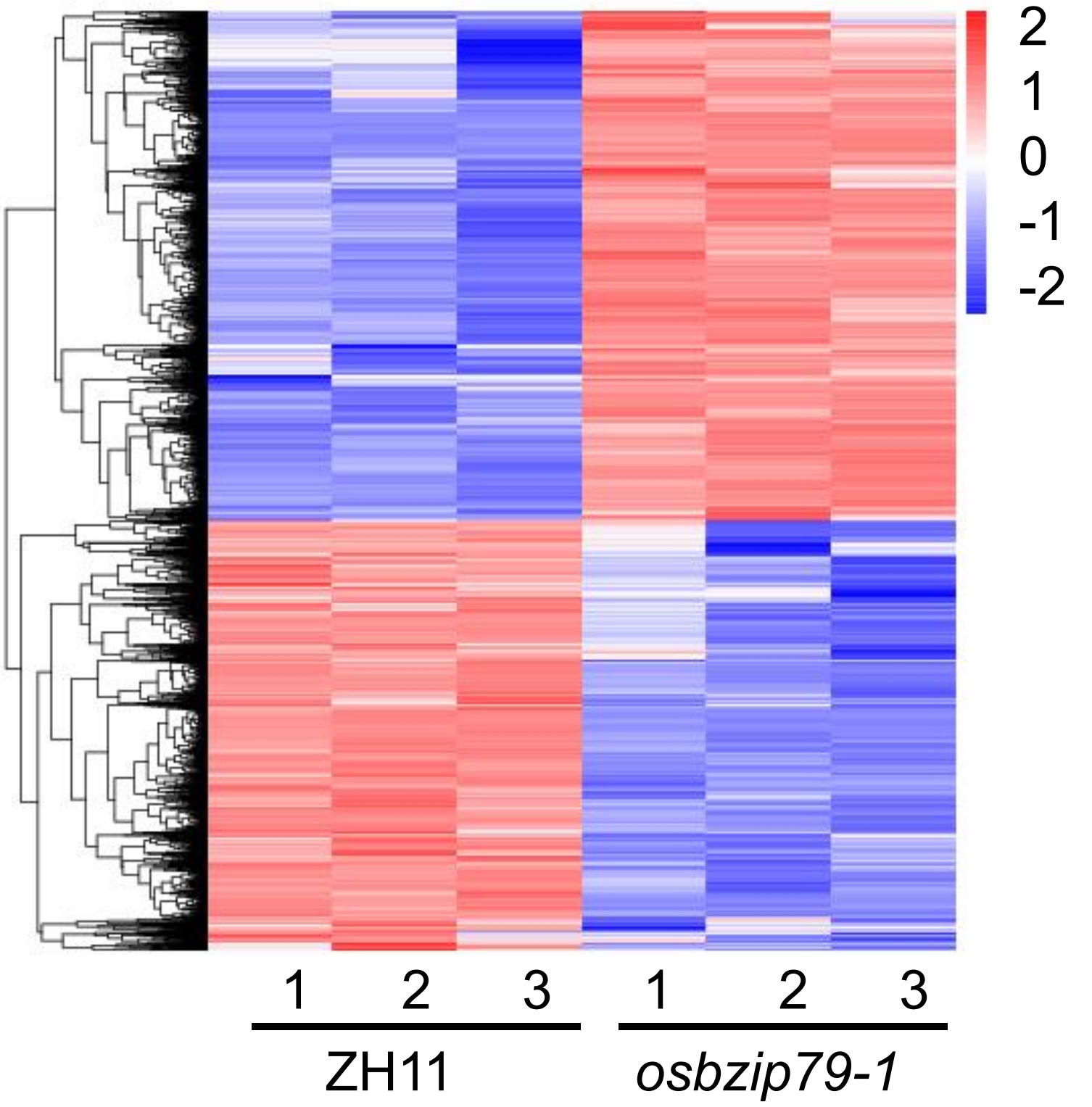
2
1
0
-1
-2
1
2
3
1
2
3
ZH11
osbzip79-1

B

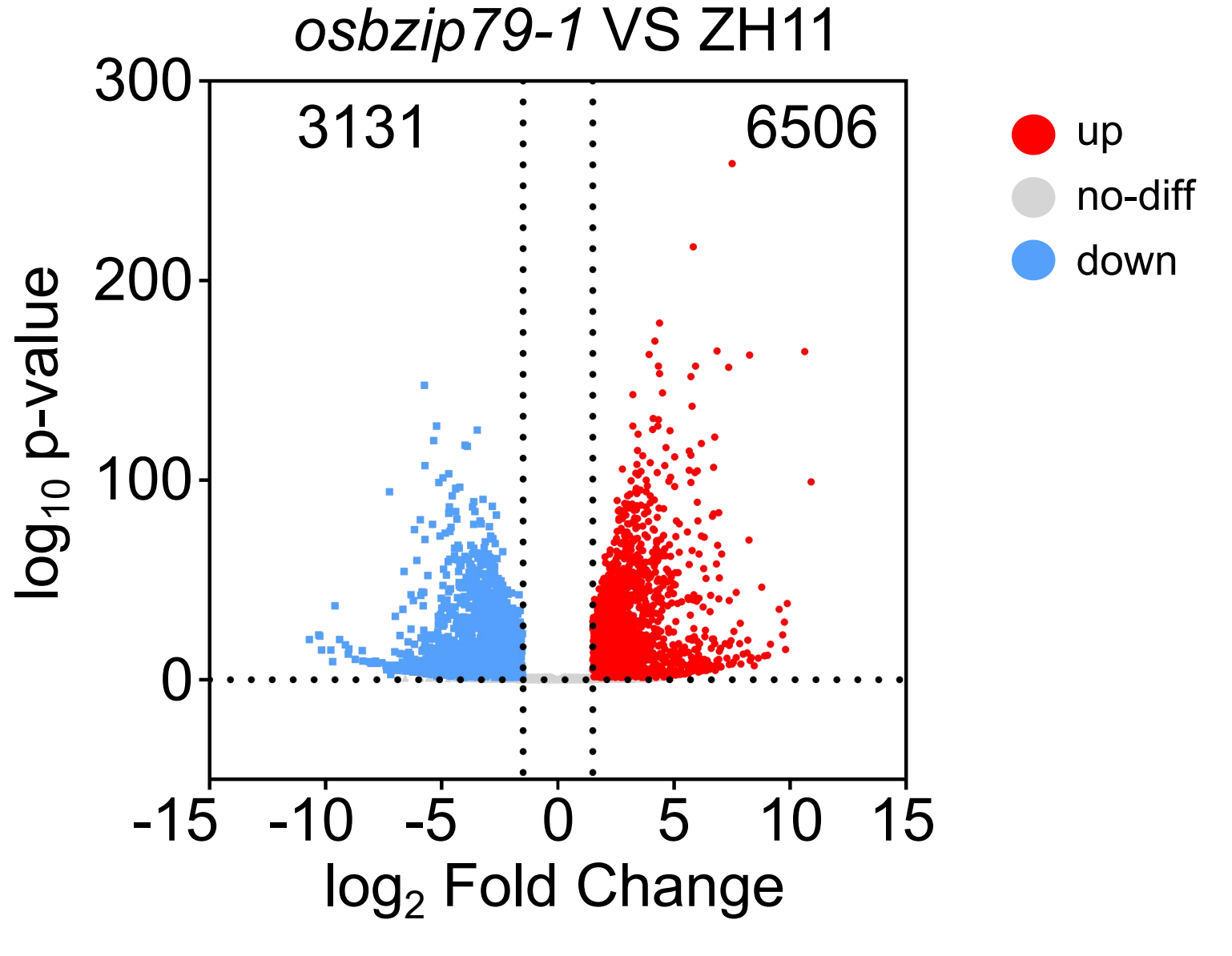
osbzip79-1 VS ZH11
3131
6506
300
200
100
0
-15
-10
-5
0
5
10
15
log10 p-value
log2 Fold Change
up
no-diff
down

Figure S4

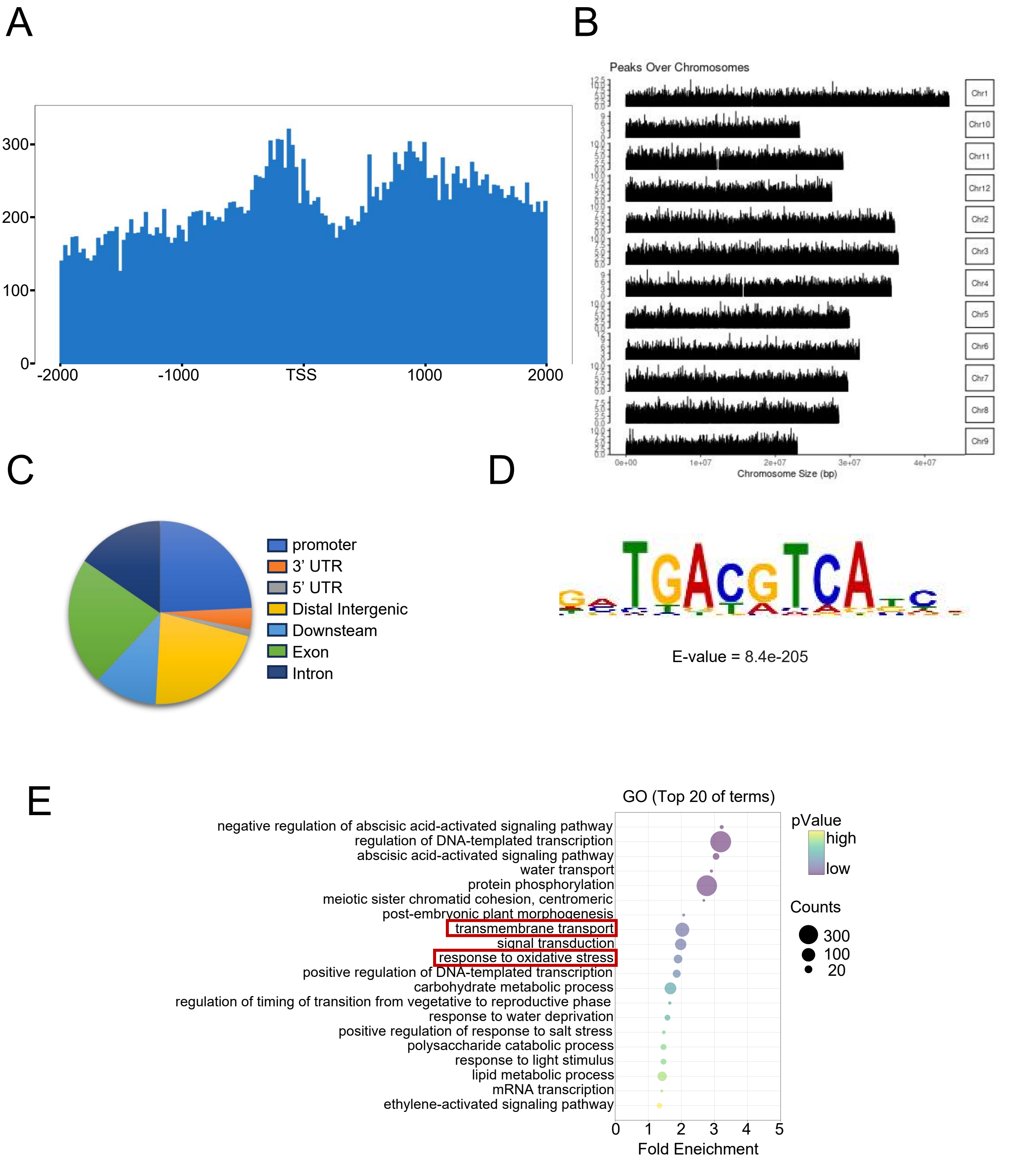
A
300
200
100
0
-2000
-1000
TSS
1000
2000
B
Peaks Over Chromosomes
Chr1
Chr10
Chr11
Chr12
Chr2
Chr3
Chr4
Chr5
Chr6
Chr7
Chr8
Chr9
0e+00
1e+07
2e+07
3e+07
4e+07
Chromosome Size (bp)
C
promoter
3' UTR
5' UTR
Distal Intergenic
Downsteam
Exon
Intron
D
E-value = 8.4e-205
E
GO (Top 20 of terms)
negative regulation of abscisic acid-activated signaling pathway
regulation of DNA-templated transcription
abscisic acid-activated signaling pathway
water transport
protein phosphorylation
meiotic sister chromatid cohesion, centromeric
post-embryonic plant morphogenesis
transmembrane transport
signal transduction
response to oxidative stress
positive regulation of DNA-templated transcription
carbohydrate metabolic process
regulation of timing of transition from vegetative to reproductive phase
response to water deprivation
positive regulation of response to salt stress
polysaccharide catabolic process
response to light stimulus
lipid metabolic process
mRNA transcription
ethylene-activated signaling pathway
0
1
2
3
4
5
Fold Eneichment
pValue
high
low
Counts
300
100
20

Figure S5

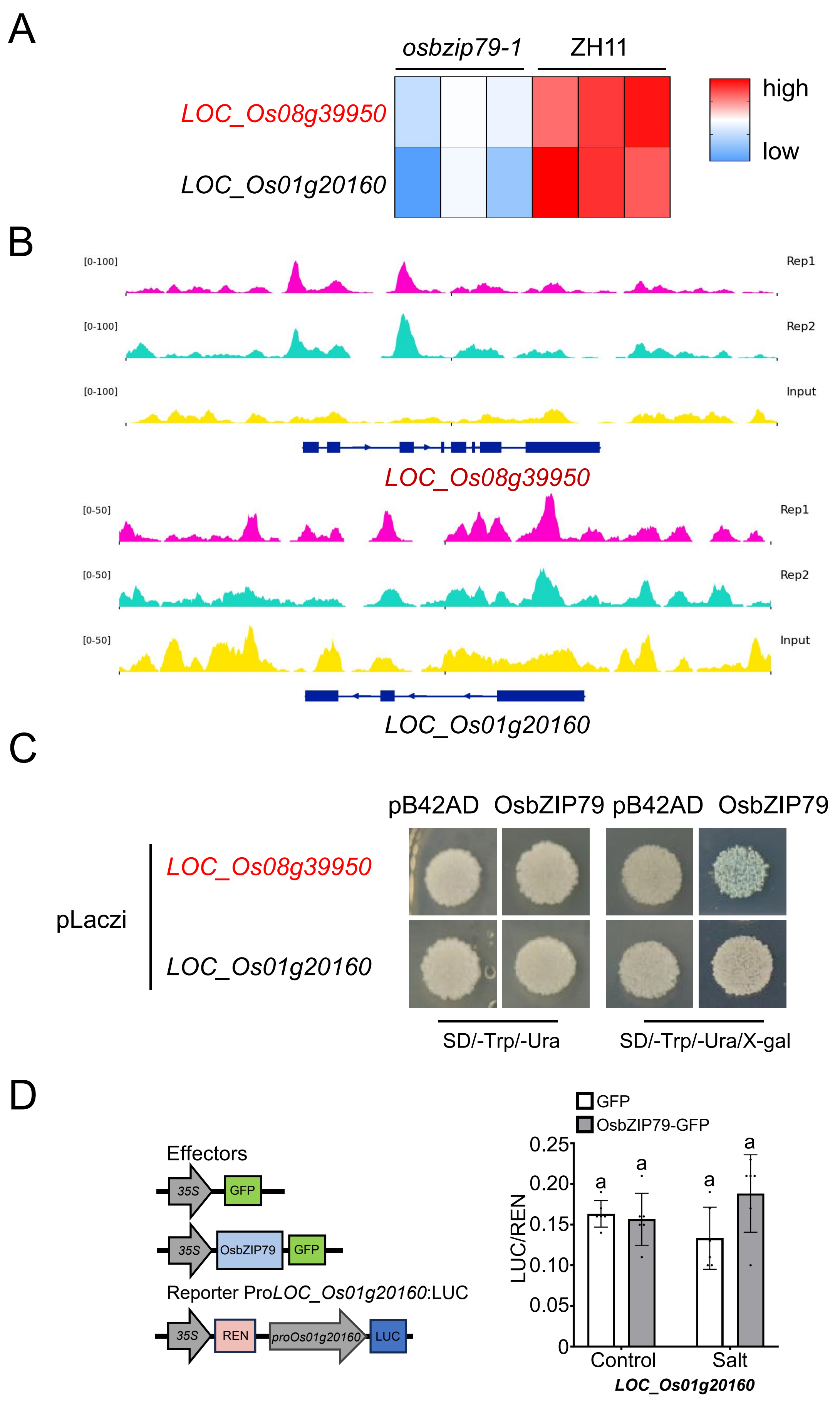
A
osbzip79-1
ZH11
LOC_Os08g39950
LOC_Os01g20160
high
low
B
[0-100]
Rep1
[0-100]
Rep2
[0-100]
Input
LOC_Os08g39950
[0-50]
Rep1
[0-50]
Rep2
[0-50]
Input
LOC_Os01g20160
C
pB42AD
OsbZIP79
pB42AD
OsbZIP79
pLaczi
LOC_Os08g39950
LOC_Os01g20160
SD/-Trp/-Ura
SD/-Trp/-Ura/X-gal
D
Effectors
35S
GFP
35S
OsbZIP79
GFP
Reporter ProLOC_Os01g20160:LUC
35S
REN
proOs01g20160
LUC
GFP
OsbZIP79-GFP
LUC/REN
0.25
0.20
0.15
0.10
0.05
0
a
a
a
a
Control
Salt
LOC_Os01g20160

Figure S6

A

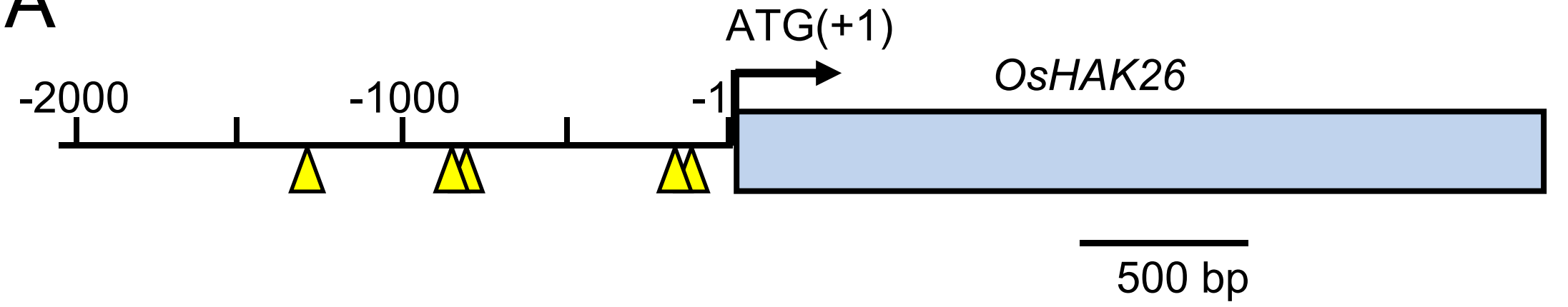

B

(-2000)
CAGTGTCGGAGCATATGATGCCAGAGTCCCAAGAACAGCAGTTGGTTGCTGGGAGCCATGAGGAGA
GCTCATCAGGGTTGCCGAGTTGCTCCTTGATCTTCAGGAGAGAGGCACGGTCACTGCTGTCGCAGT
CCATGGCGTCCGACAATAAGATGCCATTGGAGAAGAACACTAGCAAAAGGAGAGGAAACACAGGT
GCAATTGAGCTAGAGCTTGCCATAGATTTCTTGTTTGGTTATGTGACAAGAACTCTAGTGGAGAGTG
AGTGCCTACTGTTATTATAGGGGATGCCCTGATGTGAAGAAAGTAGGTGCTCAAGCAACAAACTGAT
GCGATGAAGTGGAGCTCAACCTTATCCTAAAGGGCTGCACATGGTTTTTGTTTAATTGTTTAATAATG
CACATCTAGACAAGAAAAACATGCATGATAGGTTGGTTGAAGCAGGAGTTCTCGTCTAGGTTGCTAT
TTTCAGTTCACATTTTGTAGCACAATGTGAATCATATTGTTCGTTTTCAATTGAAATGAGCAAAGCCTG
TCTGTCTGACTAATGAGACTTACATACTCTGAACTGAATATCTAATCCTTTGATGTCATGGAGGTGAG
TTTAGTGGTTTTGAAAGATGCTATTGGGTTGGGTGCAAGCCCACTAGTGGAGTTTTATAATAATCTTT
TCTATTGCAAACCAATGCCCATAGACTTCAGATACATCAGTTGTACAAGTAAGGCACGTCAAAAAGA
CCTTAGCACAGTCAGAAAGTTTATCAGTTACCAAATTGTACACATGTCAATTTTAGCTTTTGATCAAAT
TGTTGACAAATATAAATGTCATCTACAATTTGTAGGTAGTGTGTACCTTTTTTTTTCTCTCCCTTCCCT
TTTGCATGACTAGAGCAGCACATATACCATCCAAGAGAAGGGTTGTGGAAAAATAAAAATCCAAGAG
GAGGAGGCAATGATGAGATACTACCTACTCTATTGAAATGTAGAAAAAAACAATAATTTATATAAAAT
GGTGGAGCCCGGTAGACCCTTTTTATCGAAGGGCCTGCTTACTTTCTGCCACCTTTATCTTGCCTAA
ACAGGATCTTCTATTCACGGCTTGCCATCTGTGCACTGTAATTTTTTCCCTGCCATTTACGCACTGTA
TTATTTTTCCTGACCAATAAGCACCAATAAGCACGTATATACGTTGGCATGGCCTGCTAGTATATACA
TGCTTGAATTGCTTGGTTGCCACATCTTTCTATGCATTCACAATTCCAGGGTTGAATCCTGCTCAGAT
TTCACGAAATCTGGACTTTTCGGACTGGGTCGAAGACACAAACCACTCAAATTTTTTTATCAAGCTAA
AAATAATAAAATGAATTTTAATTTCTTTTAACAAAATTTATTTGGTGTTCATCAATGGATTCCACAAAAT
CCGATGTTATGGCTTGTGAGCAGATTTCAATCCGATATTAGGATCGTGACCCTTGATTGTCAGTATCA
CCCTTTTGGAGATATATTTTTAGCGCGTGTAAAATGAAAAAGCTAATTAGCACATGATTAATTAAGTAT
TGATAATTATAAATTTGAAAAATAGATTTATTTGAAATTTTAAACATCTTCCATATATGTTTTTTTAAAAA
AACGCATTTAATTTAACAGTTTGAAAAGCGTTCTAACAAAGAAAATAAAAAAAAAAGTTAAAGTTCAGA
GTTGAAGAATAGAACTGGGTCTAATAAGAAGATGACCAGTCAAGCTTGTGAAAGGGAAACCGTTGAA
TATAAACGGACGGTGGAGATACCCGGGACGTGACACGTCACCATTATTCTGTTGTCAACTGCATGCC
GCAACAAACTGAGTAAACCCTACCACCCCCCAACCTCTCCCCCCTATAACCTGGGAGCACCAACTA
CAAAAACCCATGGTGAGATCGATCGATATCGATCGATTGATTGCTATAGCTAGGTCAAATCGATCGA
TCTCGATCGATCGGGTGGTCGATCCGATCAGCAGCC
(-1)

Figure S7

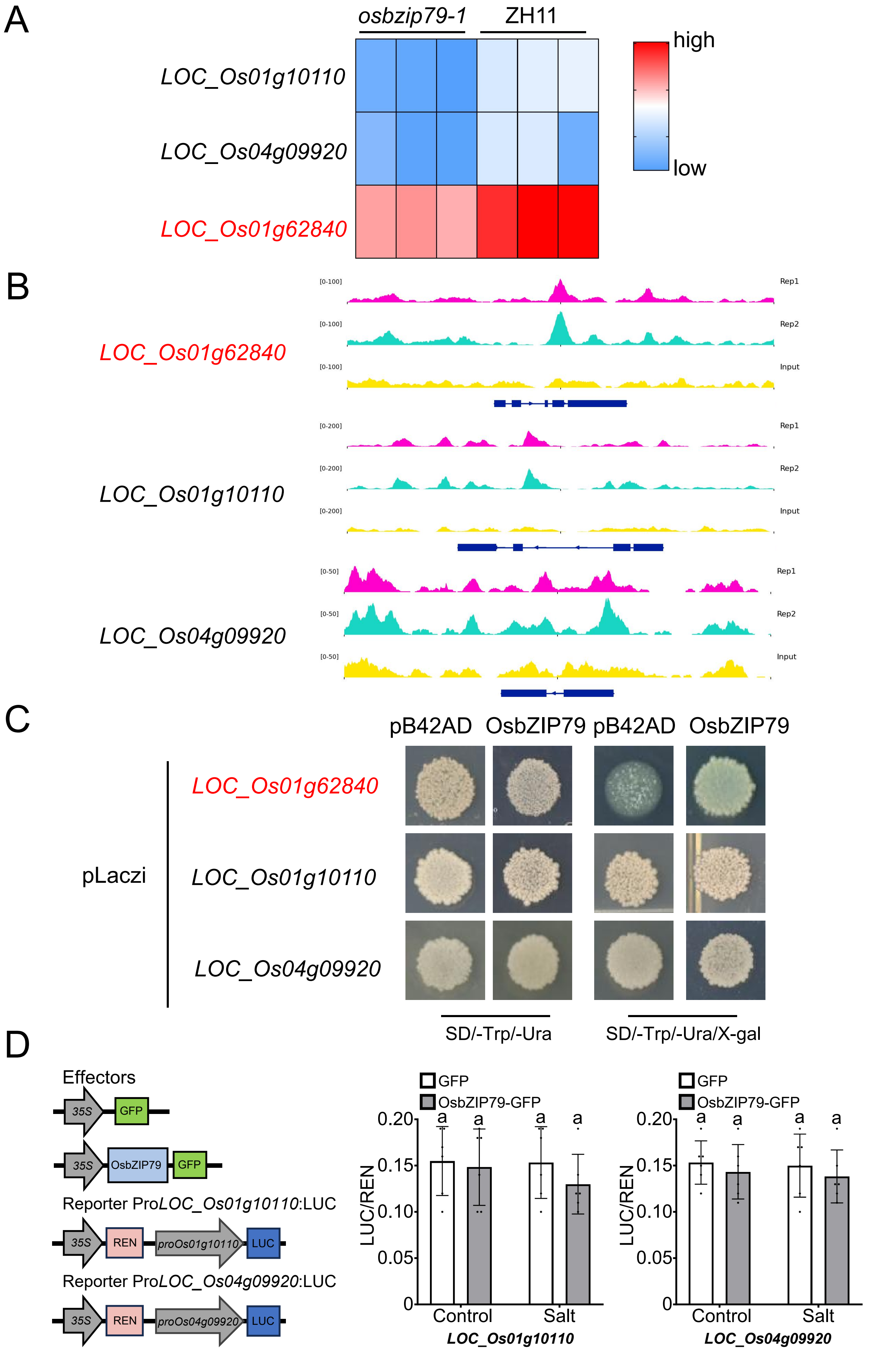

A
osbzip79-1
ZH11
LOC_Os01g10110
LOC_Os04g09920
LOC_Os01g62840
high
low
B
LOC_Os01g62840
LOC_Os01g10110
LOC_Os04g09920
[0-100]
[0-200]
[0-50]
Rep1
Rep2
Input
C
pB42AD
OsbZIP79
pB42AD
OsbZIP79
pLaczi
LOC_Os01g62840
LOC_Os01g10110
LOC_Os04g09920
SD/-Trp/-Ura
SD/-Trp/-Ura/X-gal
D
Effectors
35S
GFP
35S
OsbZIP79
GFP
Reporter ProLOC_Os01g10110:LUC
35S
REN
proOs01g10110
LUC
Reporter ProLOC_Os04g09920:LUC
35S
REN
proOs04g09920
LUC
GFP
OsbZIP79-GFP
LUC/REN
0.20
0.15
0.10
0.05
0
a
Control
Salt
LOC_Os01g10110
LOC_Os04g09920

Figure S8

A

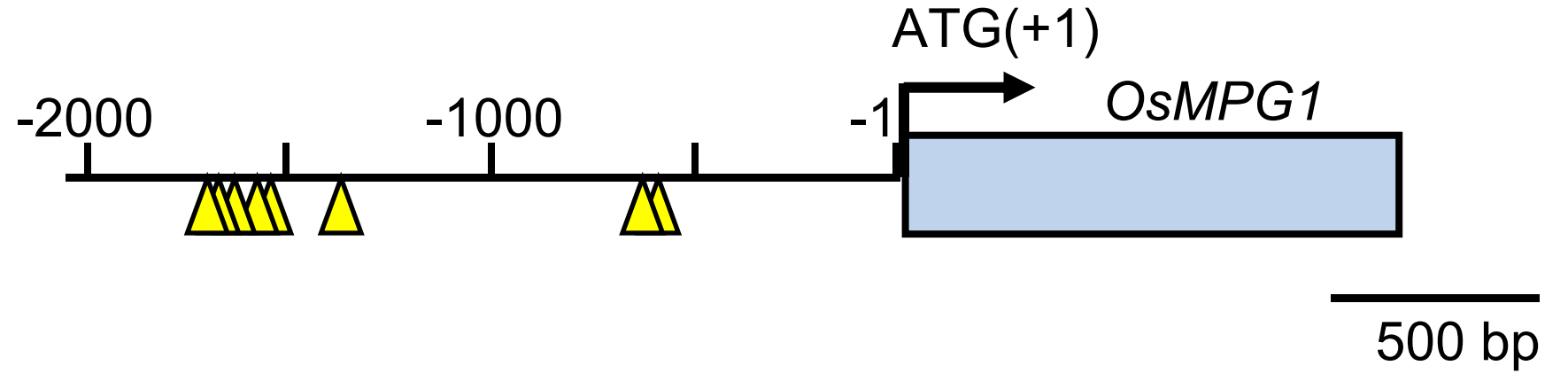

B

(-2000)
CCTGATTCACGGGCTCAGCGACGCCGCCGCCGCAGAGCTCATCTACGCCGGCAGCCACTCTTCC
CCAATGCCGCGCTGTCGCCGCACAGCCTTCGAGCTTCCTCCACTCTCTCTCTCTCTCTCCACTGT
CACCGCCGCCAGCATTCTTGGGAGCCACCCGCCGCCGACCGCACTCGAATCTCCAGTGCCACCG
CATCTTGGGCCACCTCCGTCGCCGTCGAGGTCGCCCATAGCCATTGGAGTCGTGCGCCCTCCTC
TGGCCTTCCTCCCTGTGCGCAGGAAAGAGAGGAAGAAGAGAGAAAGAGGGATCTAACATGCGGG
TCCTACCTTTTATTATTTTATTATTTCCCCAGAAACTGCCACGTAAGCCCCACGTCAATCTCACGTG
GGATGAAGAACTAGTAAAAGAAGCTACGTAGGTGTCACGTAATCAAAACTGGGGACAATACCACC
GGAGGACCTCATTTGCACTGTTTTTGTAAGTTGATGGATGAGTTGTAACTGGGTTTTTTTTTAGGGA
CGATTTATAATTCGGTTACAAGACGAGGGATCTCCGGTGTACTTTTTCCTTAGACAGCCAGTAGCT
TTTCCAAGATTCTGTTTTTTCAGGCCCAGTTCATCTGGCCACAGAGAGGCCCATCATCACGTAACG
AAATGTAACCCGACTAGGCCTTATGGCCCAAAAAGTCGTAAGAACGGCTAAGCCTTATCCAAAATT
CCGAAGCTTATCACGAAACGGACAATAAGGTATACACCCTAATCCCAAAAACCATTGAACTCAAAA
GGGGAATAAAATATCGACTGGGAGGTTTTTTCTGACGGTTGTATCACAGGATCACACAGTATTACT
AGTGTATTACTGCAGCGTTTTCAGATCATAAACAGATTCTGGCGGCACCATACACGAGAATACGAG
ATTAAGTAATCACAAGATCAGCTTGGAAGCAAGGAAGAAAAAGAAAAGAGCACCGACACGGCATTT
CTCTCGCATGCGCGTTTGCGTATCAGTGAGTGCGCATTTGGGTCCCGTCACTCGCAACCAAAGAA
AGATTAGTCGAAGCAAGGTGCAGATATCTTGGATACCGGAGAGCCGGTTGACCCTAGAAAGAGGA
GCCAAGATCATCAGATCTGCCGGGTTTCCGGACGCATCAGGATTAGCACATGGAATATCCTATGTT
ATGCGTGATTACAATGGGCAGCGCAATGCCTCGCCTTGGTTGGTTGCCTGCATCTACAGTCTGGA
CAGATGACAGTCCGAAGCGGCTTCATTGTGACAAAAAAGAAGAAGCAATCTTTGGTTTCTTGGATC
AGGGAAAGTGGAAAGCATAAACTGGAACAGAAGGCCAGGCGGAAATAAACTGGACAAGAAGAAAA
GGCGTATGTAACGTACTAACGTGCATGTTTTTTTCCTCATTCAAAGATTTCCCCTCGACTTCTCATT
CAAACAAAGCAAACATAGCGTTTTCTAAAATTTATTTATTTTAATAATATTCTCCCATGATTAGAAGG
GGATTAAAAGAAAATCTCTTCCGTTGACAGAAACCACATCTCTACTGTAGTTATCTGAATTCTGAAA
GCCTGAACCTCTCGTCGTGTCTCGTACTCTCGTTCCCCTCCCCTCGCGTCGCCTCTCCACTTCGT
CTCTATAAAGTCCCACCCAATTCTCGATTCCCAATCCAGCGCAGCACAAAGCCCTCCTCCTTCCAC
CGCAGCTGCGCTACCTACCTCCAAGAGGGCTGGTCCGCTGGTCGTGGAGAAGGAAGAGAGATCT
TCCAGGCTGATCCCTCCTATTGGTACGGAAGCCCTAAACTCATCGGAGCTTTGCCGCGGCCGCGA
GCGATCAAATCGAAATCTTTCTTTCTTTGTTCTTTGATGCTCCAACCTAGTGTCGGATTTGATTTTGA
CTGTTGCAATGGCAGCTTTCTCCTTCCCGAGCCCGCTTGAGGATCCCCGCCGGAGTAGGGATTAG
GGAGGGGAGAACAGGAGGCGGCGATTCACC
(-1)

Figure S9

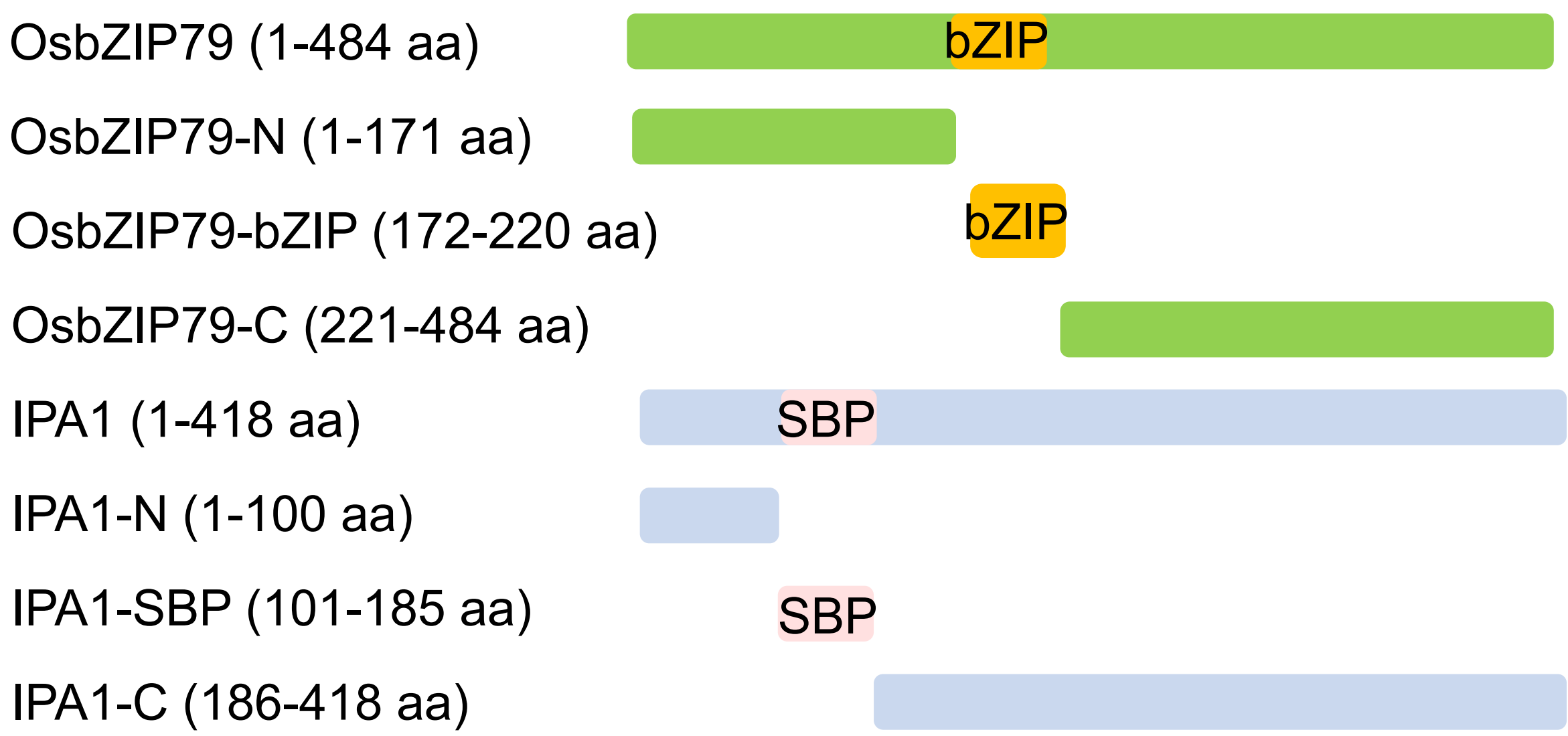

OsbZIP79 (1-484 aa)
bZIP
OsbZIP79-N (1-171 aa)
OsbZIP79-bZIP (172-220 aa)
bZIP
OsbZIP79-C (221-484 aa)
IPA1 (1-418 aa)
SBP
IPA1-N (1-100 aa)
IPA1-SBP (101-185 aa)
SBP
IPA1-C (186-418 aa)

Figure S10

A

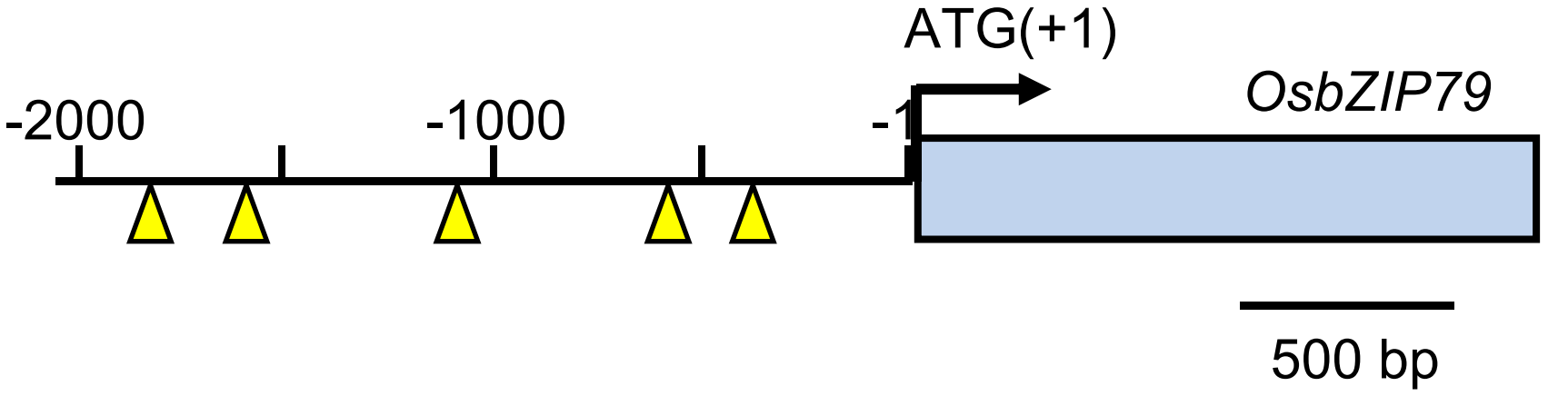

B

(-2000)
TCACCCTTTAAAAAATGTTCTATAGTTTGTTGGTAAAAAATGGTGATAACTGGCAAGAGGGCGATCAA
GCATAGTAGGATAACACTTACAGACCATTGATAGATGCATCTGATGTGGAATCACACAATAGCGGAG
TTCTCTTTCAGTTTATTAATCATGGTGGTAAACCATATATAGTTTTCACGAGTGTACAATATAGTGTGC
AATACATCAGTCCAATTGTATGGCCAGTAGCTCAGGTGTAGCTTTAGGGGTAAATGGTAAAGTCAGA
ATATATAAATAAATAGGTATGCAGTGTTGCTTTCAGTTGCTGTCTAATTCCTGTACATGGAGATGTGA
AAACATATACAAATTGAGGAGTCCCAATCTAACACATCAATAACAAGTGAACGTATGATAGATAGCAA
AACAAGCACATTGATTGATTAGACTTAAGAATCGACTCAGGAATTTAATATGTTACCTATCCATCGTG
GAAATGAATATTATTGGCGAGGTGTGGGTTTAGGTGTGGTTTGACAAAGGCATGGATGTTGACACAT
TAACAGGGGACAGTGTCAAGCAAATTAGAATGATGAATAATTAAATACTACAATATAAACCTCTCCAT
GTGATTTATTTTTGCTTTCTTACTGCATAATTGGGCATGGCATACTGAAAAACATTTCAAATTAATGGC
ACTTACTAGTAGATATAAGATTTTTTTATCAAGATTTTGGTTGATTGCTGTGGCTTTATCTAAAGATATT
TCTTTTAGACTATTCTATCTTTAATTAGCAAAAGATTATACACCATCTTTTCACAAGCTTGCATTTCTAG
CCTGAGGTGACATATTTGCAGTGGATGTCGTGGGGCCAGGGGGTGTGTATAGATCAAGGTACTCCC
TCCGTCCCATAATATAAGGGATTTTAAGTTTTTAATTGCAGCGTTTGACCACTCGTTTTATTCAATTTT
TTTTGCAAATATAAAAAACGAAAAGTTGTGCTTAAAATAATGTAGATAATAAAGTAAATCATATATAAA
ATAAATAATAAAAAAATTTAAATAAGACGAGTGGTCAAACGTTACAAACAAAAACTCAAAATCCCTTAT
ATTATGAGACGGAGGGAGTAGCATACAATGGTTTTGTGCAGTAGTTTCTAACTTTTCCAGTTATCTGA
TAGTGTAGATATTATAATGAACTTCATATAAATCAACCAGCAATTAGTAAGGTCAGCGGTTTGAATCA
AAGAAATACCAATCATGAATTAGGTCATTTTCATGCTCAAAATCTAGATGGTAGAAATGGCCTGAATT
ATTGAAAATACGGAACAATTGTACCAAGTCTATGATCTTACCAGTGTGAAATTGTATATTTTTTCTACT
ACTCTTAAAACTGATAATACTATAACTCATGGGTGAACATGAAATAAAGAAATAAGATTGCTGGGGCC
AATGAAAGAGAACAGTCAGTTCCAATGTGATAAGAGTGCAGTCAGGGATTCCTGTGGCTTCATGACA
AAAACCTTCGAGTGAAGACAAGACCCTAGACCGTCTCTCTCTCTCTCTGACCAAGGCTAGTAGGGAA
GGAAGAACAAAAACAGCATAGGAAGGACAACATCTTTCTCTGAACTGTGGGATTAGTACGCCCAAGA
ACGTGAAGCTATTACACCGTCAGATATATCAAGAAGATGCAGGCGGAGCAAGAGCAACTGCGCACA
GAAACGGTCAGGCATGCCTGCTCTCCCTCCTGCTTGTCATAAAAGGGAAGCCACAAGCTGGTATGT
TTTCTTGACTCAAATCTTCTTCGTTCAGATGTATGCTTGCTGAGAGCCTGAGATAGGTGTATTTGTGT
AGTATCGATCTTTGATGTTTTTCCTTCTTTTTTTGTGCGTTTTGAGCGCAGAAGCCAGATTGCTAGAA
GGTAGTTCTTGAGGACTGAAGCAAGGTAGAAGAACTTGAGCTTCTGGTTCGTGTGGAGCTACTGGG
ATAAGTTGGCTGCTGATTGCTGAAG
(-1)

Figure S11

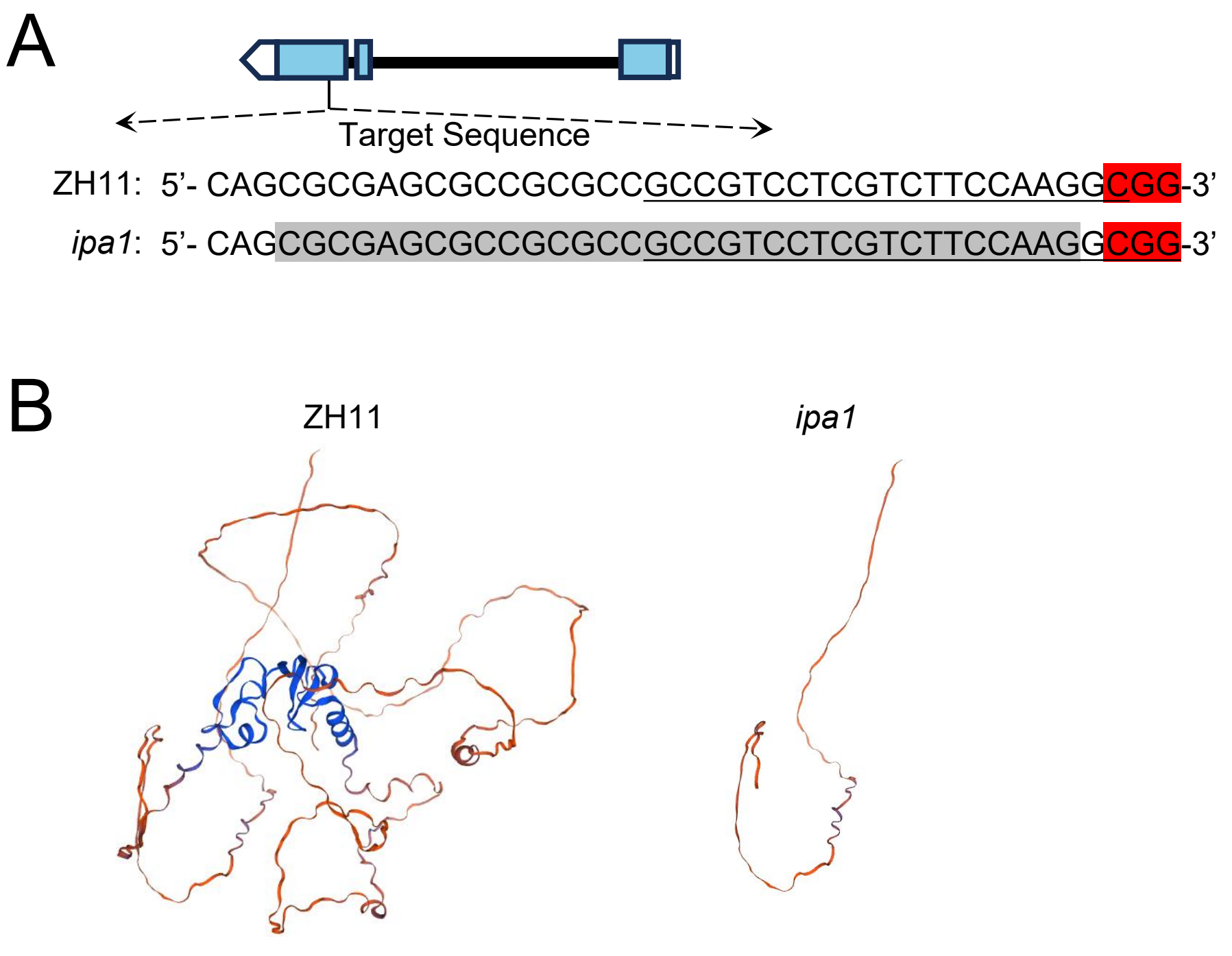

C

```
ZH11: MEMASGGGAAAAAGGGVGGSGGGGGGGDEHRQLHGLKFGKKIYFEDAAAAAGGGGTGSGSGSASAAPPSS  70
ipa1: MEMASGGGAAAAAGGGVGGSGGGGGGGDEHRQLHGLKFGKKIYFEDAAAAAGGGGTGSGSGRRRRVVDAAE  70

ZH11: SSKAAGGGRGGGGKNKGKGVAAAAPPPPPPPPRCQVEGCGADLSGIKNYYCRHKVCFMHSKAPRVVVAGL  140
ipa1: GARTRGRAWPRRRHRRRRRRRGARWRGAARI———————————————————————————————————————  101

ZH11: EQRFCQQCSRFHLLPEFDQGKRSCRRRLAGHNERRRRPQTPLASRYGRLAASVGEHRRFRSFTLDFSYPR  210
ipa1: ——————————————————————————————————————————————————————————————————————  101

ZH11: VPSSVRNAWPAIQPGDRISGGIQWHRNVAPHGHSSAVAGYGANTYSGQGSSSSGPPVFAGPNLPPGGCLA  280
ipa1: ——————————————————————————————————————————————————————————————————————  101

ZH11: GVGAATDSSCALSLLSTQPWDTTTHSAAASHNQAAAMSTTTSFDGNPVAPSAMAGSYMAPSPWTGSRGHE  350
ipa1: ——————————————————————————————————————————————————————————————————————  101

ZH11: GGGRSVAHQLPHEVSLDEVHPGPSHHAHFSGELELALQGNGPAPAPRIDPGSGSTFDQTSNTMDWSL  417
ipa1: ———————————————————————————————————————————————————————————————————  101
```

Figure S12

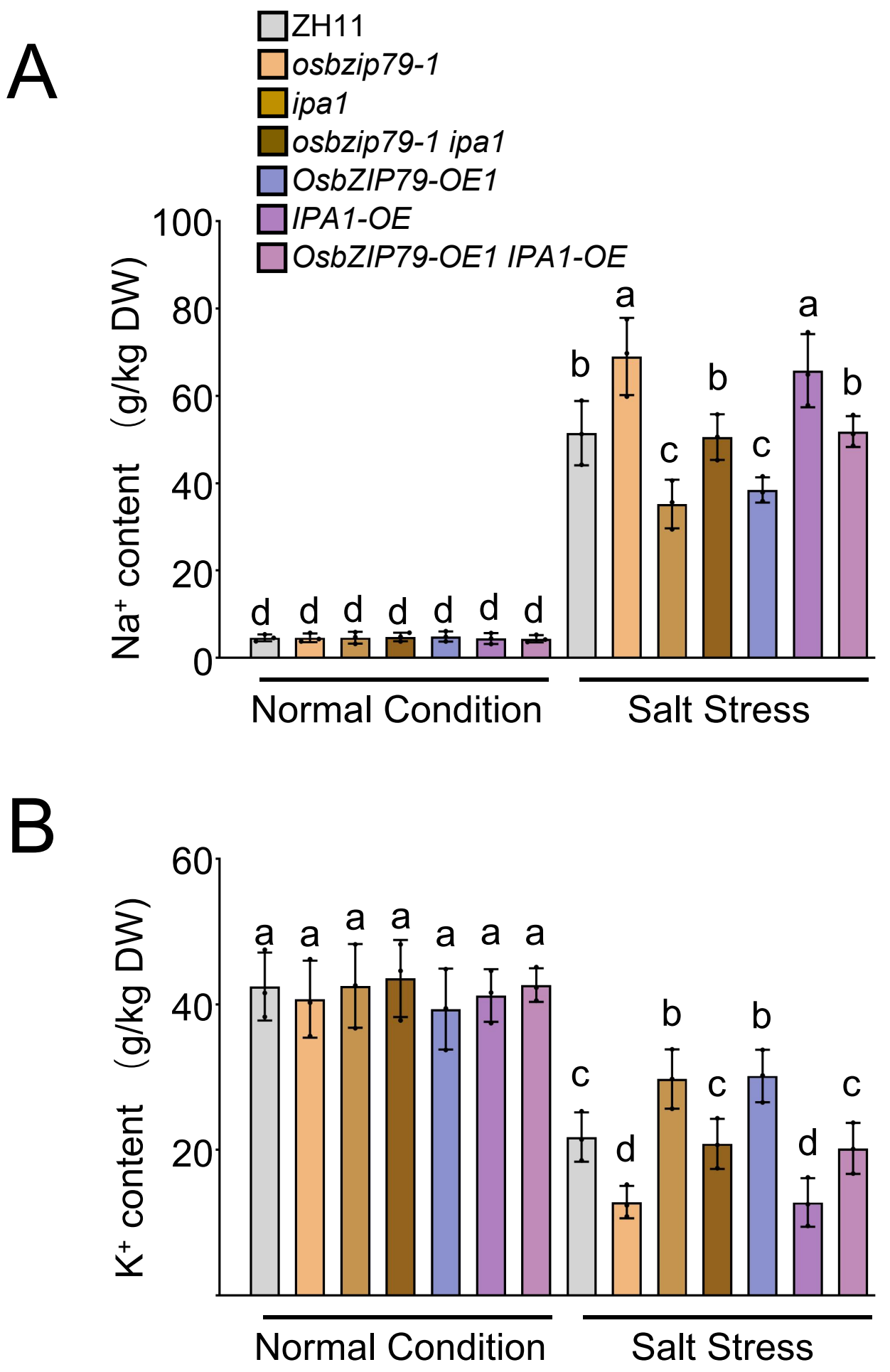
A
ZH11
osbzip79-1
ipa1
osbzip79-1 ipa1
OsbZIP79-OE1
IPA1-OE
OsbZIP79-OE1 IPA1-OE
Na⁺ content (g/kg DW)
100
80
60
40
20
0
d d d d d d d
b a c b c a b
Normal Condition
Salt Stress
B
K⁺ content (g/kg DW)
60
40
20
a a a a a a a
c d b c b d c
Normal Condition
Salt Stress

Figure S13

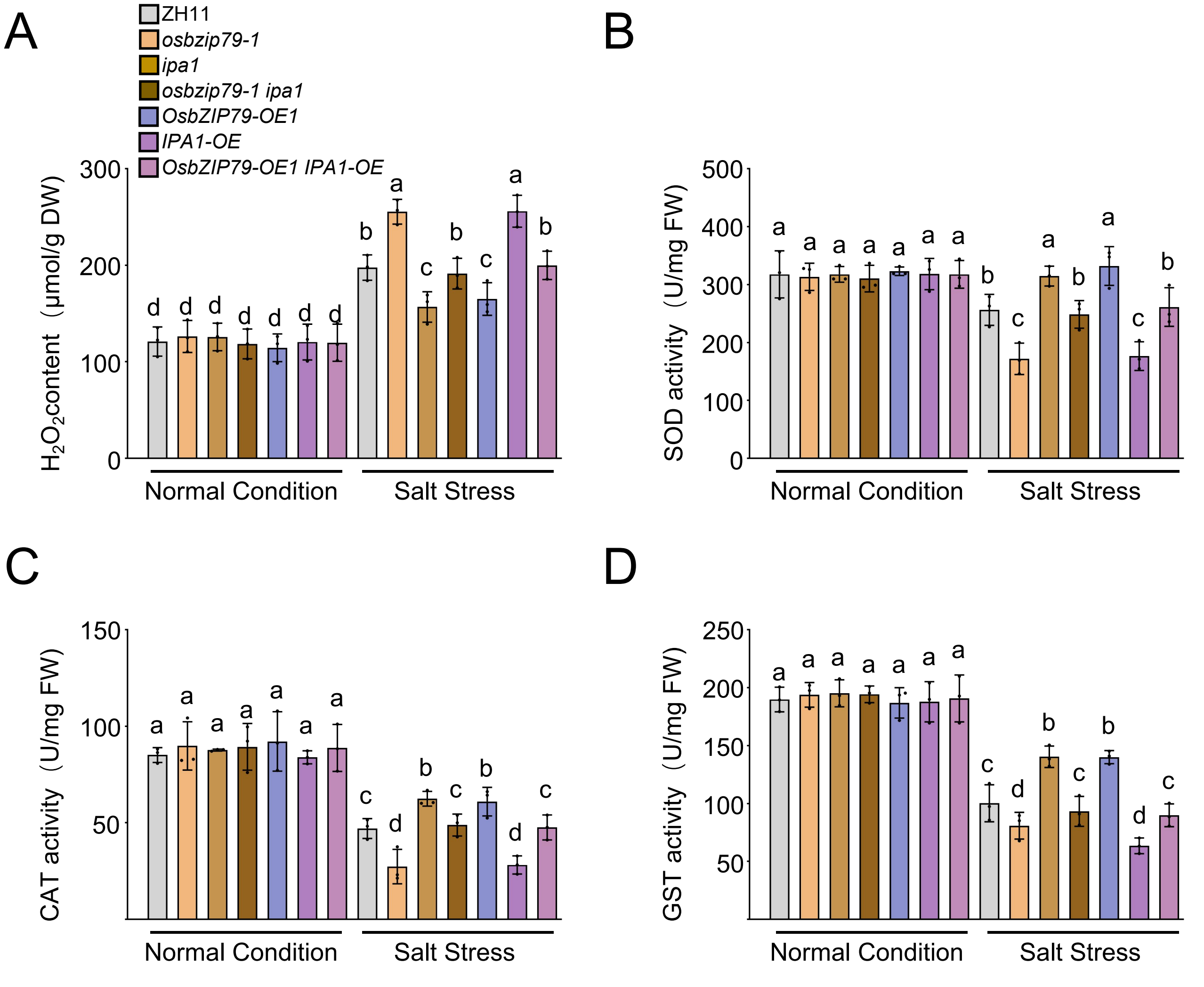
A
ZH11
osbzip79-1
ipa1
osbzip79-1 ipa1
OsbZIP79-OE1
IPA1-OE
OsbZIP79-OE1 IPA1-OE
H2O2content（μmol/g DW)
Normal Condition
Salt Stress
B
SOD activity（U/mg FW)
C
CAT activity（U/mg FW)
D
GST activity（U/mg FW)

Figure S14

A

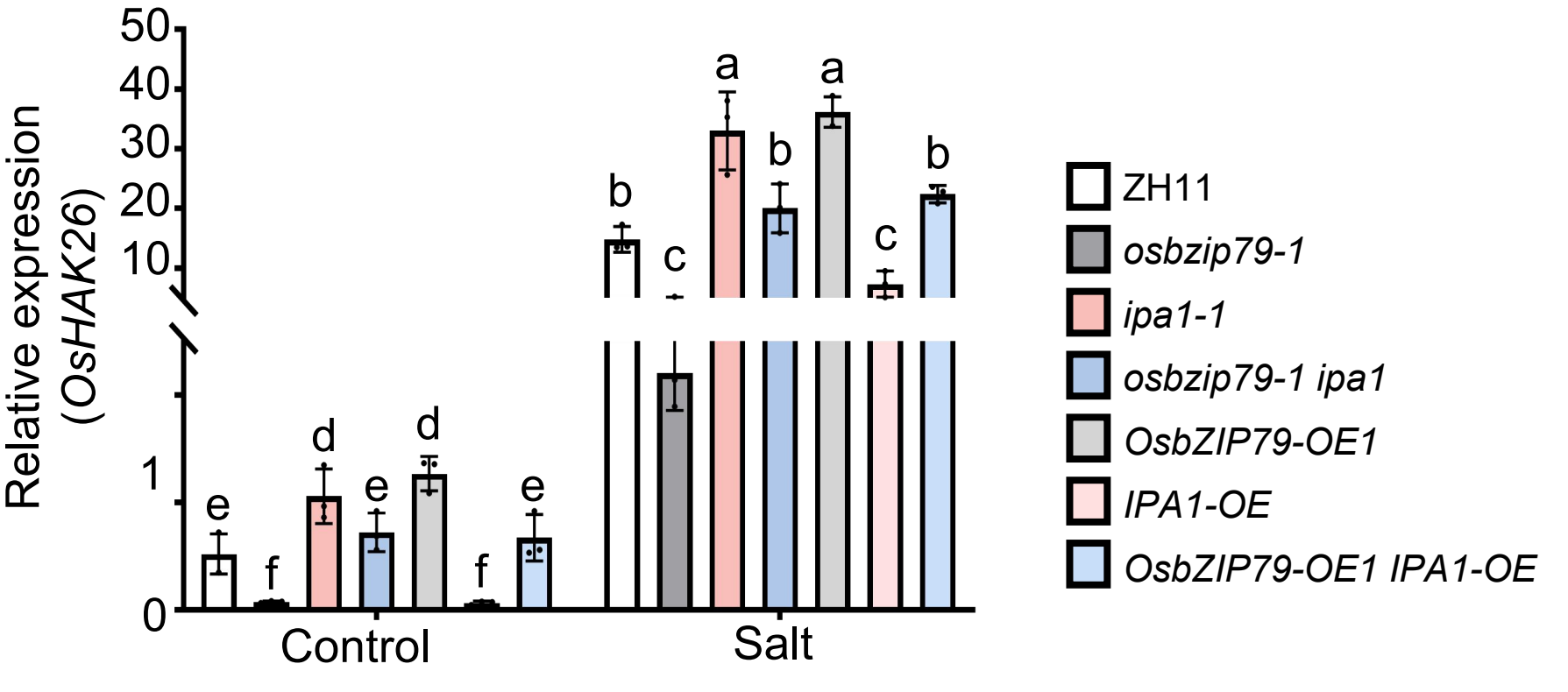

B

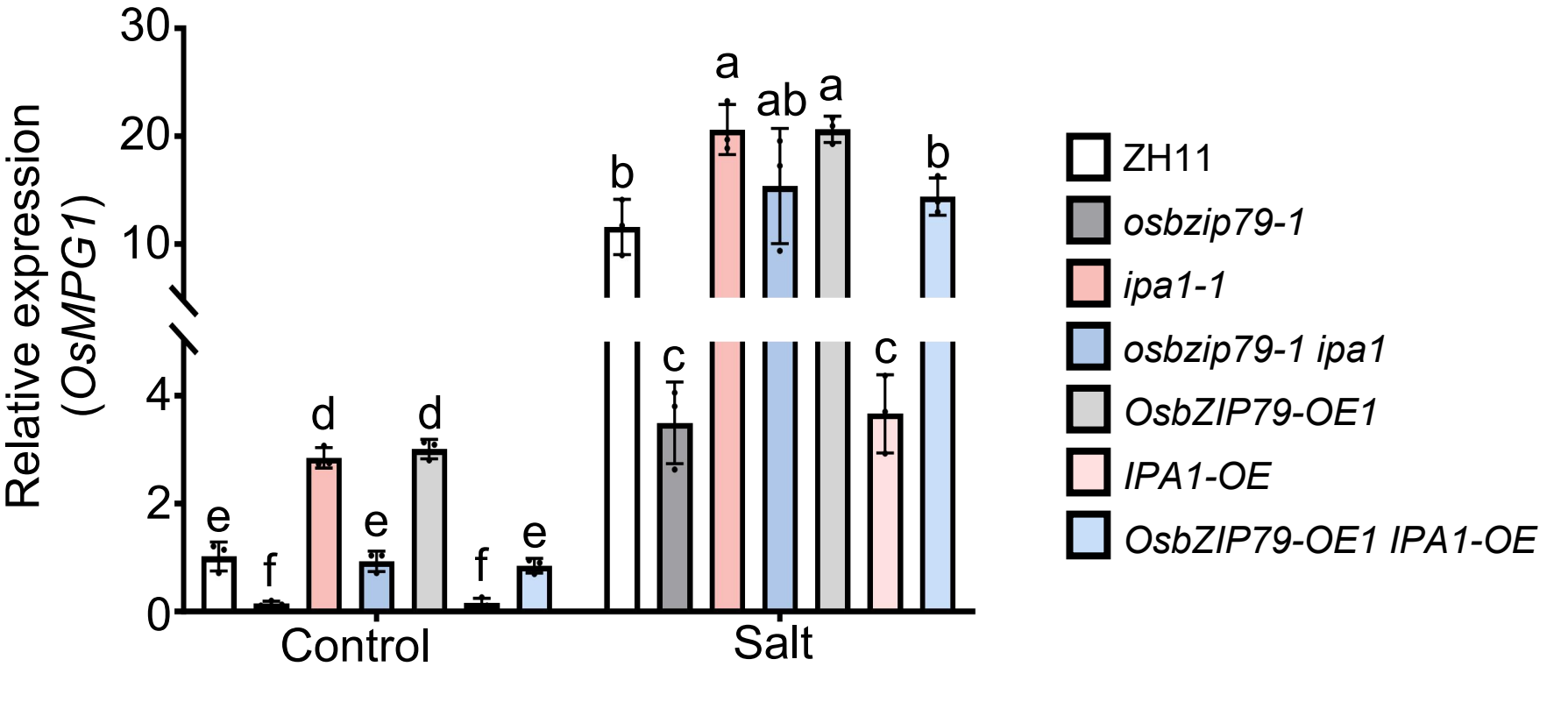

Figure S15

A

#	Name	Position	ObsHET	PredHET	HWpval	%Geno	FamTrio	MendErr	MAF	Alleles
#1	2464187	2464187	0.018	0.455	0	99.1	0	0	0.35	T:G
#2	2464633	2464633	0.02	0.456	0	99.4	0	0	0.352	A:T
#3	2464952	2464952	0.018	0.458	0	99.6	0	0	0.355	C:T
#4	2468483	2468483	0.019	0.456	0	99.4	0	0	0.352	C:T
#5	2468554	2468554	0.019	0.456	0	99.3	0	0	0.351	G:T
#6	2469123	2469123	0.016	0.354	0	97.9	0	0	0.23	C:T
#7	2469127	2469127	0.016	0.455	0	97.2	0	0	0.351	C:T
#8	2469186	2469186	0.017	0.48	0	92.6	0	0	0.4	T:A
#9	2469194	2469194	0.013	0.327	0	92.7	0	0	0.206	A:T
#10	2469214	2469214	0.014	0.458	0	90	0	0	0.354	T:A
#11	2469220	2469220	0.013	0.458	0	91.1	0	0	0.355	C:T
#12	2469295	2469295	0.012	0.341	0	95.8	0	0	0.218	G:C
#13	2469835	2469835	0.02	0.455	0	99.2	0	0	0.351	G:T
#14	2469947	2469947	0.019	0.456	0	99.3	0	0	0.352	G:A
#15	2470047	2470047	0.019	0.457	0	99.2	0	0	0.353	G:C

B

TagSNPs	Alleles	r^2
2469220	2464187	0.97
2469220	2464633	0.966
2469220	2464952	0.96
2469220	2468483	0.97
2469220	2468554	0.971
2469220	2469127	0.973
2469220	2469186	0.838
2469220	2469214	0.995
2469220	2469220	1
2469220	2469835	0.972
2469220	2469947	0.967
2469220	2470047	0.964
2469295	2469123	0.956
2469295	2469194	0.955
2469295	2469295	1

C

	ALL	Indica	Aus	Japonica	Intermediate
Hap1	1785	140	57	1466	122
Hap2	1446	1365	61	3	17
Hap3	892	777	80	20	15

D

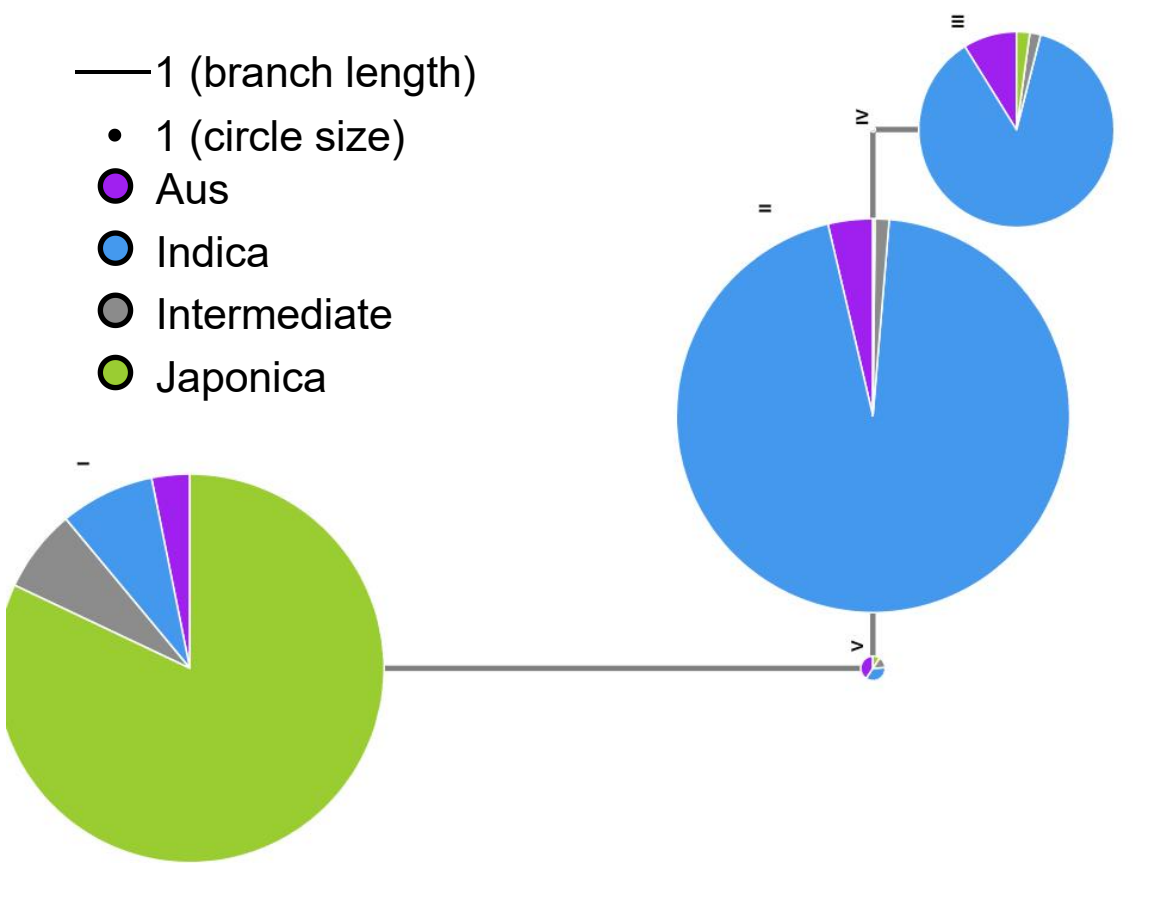

E

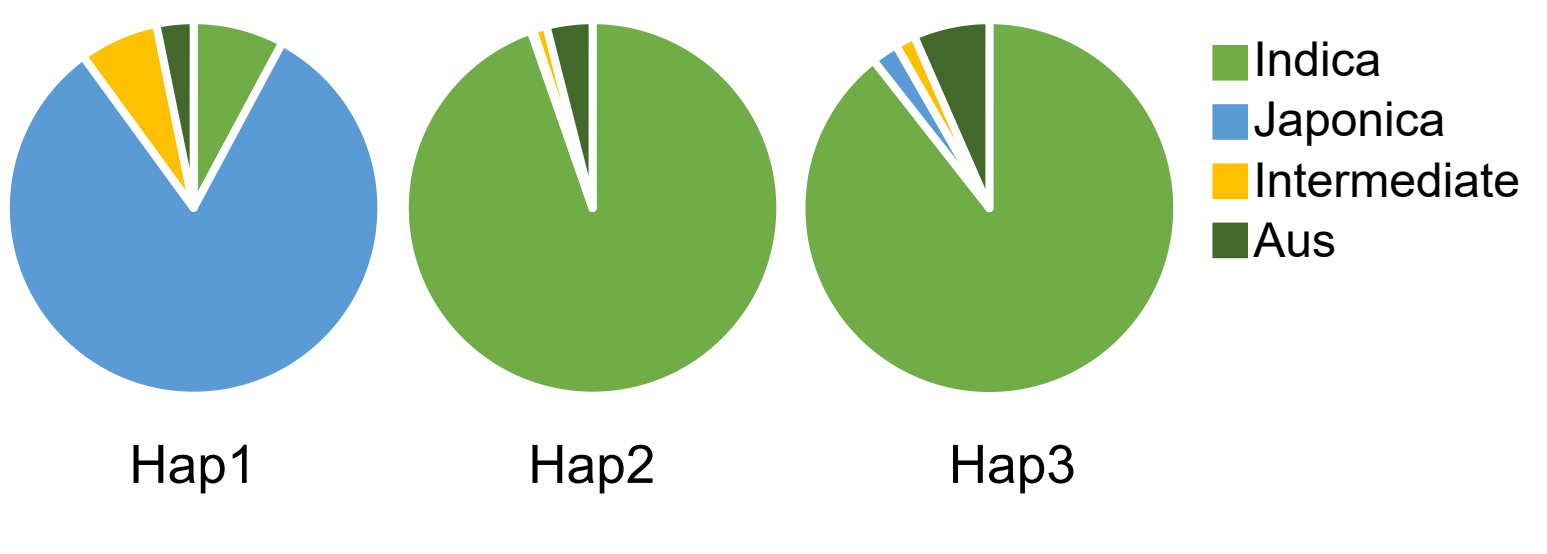

Figure S16

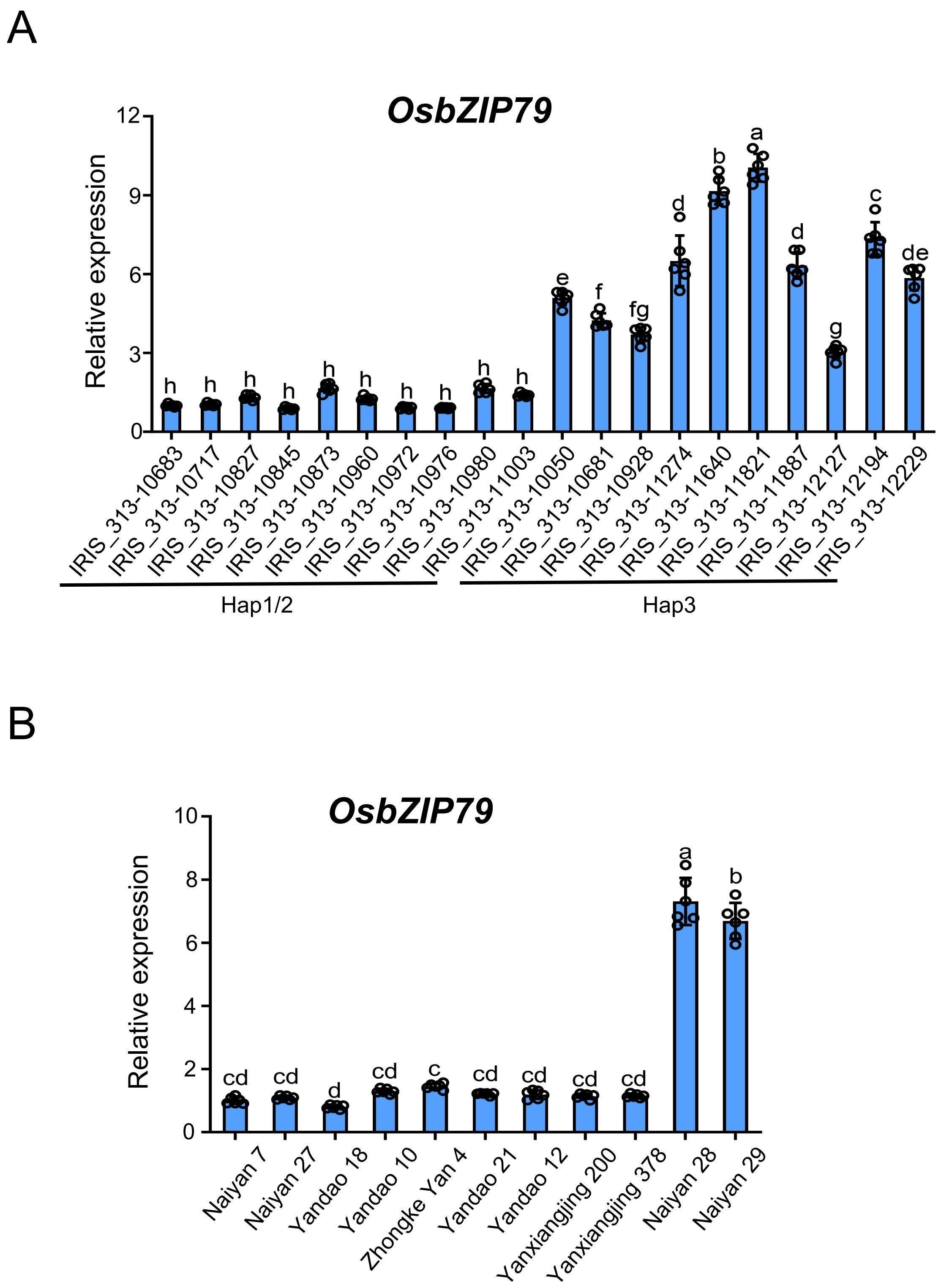
A
OsbZIP79
Relative expression
0
3
6
9
12
h
h
h
h
h
h
h
h
h
h
e
f
fg
d
b
a
d
g
c
de
IRIS_313-10683
IRIS_313-10717
IRIS_313-10827
IRIS_313-10845
IRIS_313-10873
IRIS_313-10960
IRIS_313-10972
IRIS_313-10976
IRIS_313-10980
IRIS_313-11003
IRIS_313-10050
IRIS_313-10681
IRIS_313-10928
IRIS_313-11274
IRIS_313-11640
IRIS_313-11821
IRIS_313-11887
IRIS_313-12127
IRIS_313-12194
IRIS_313-12229
Hap1/2
Hap3
B
OsbZIP79
Relative expression
0
2
4
6
8
10
cd
cd
d
cd
c
cd
cd
cd
cd
a
b
Naiyan 7
Naiyan 27
Yandao 18
Yandao 10
Zhongke Yan 4
Yandao 21
Yandao 12
Yanxiangjing 200
Yanxiangjing 378
Naiyan 28
Naiyan 29

Figure S17

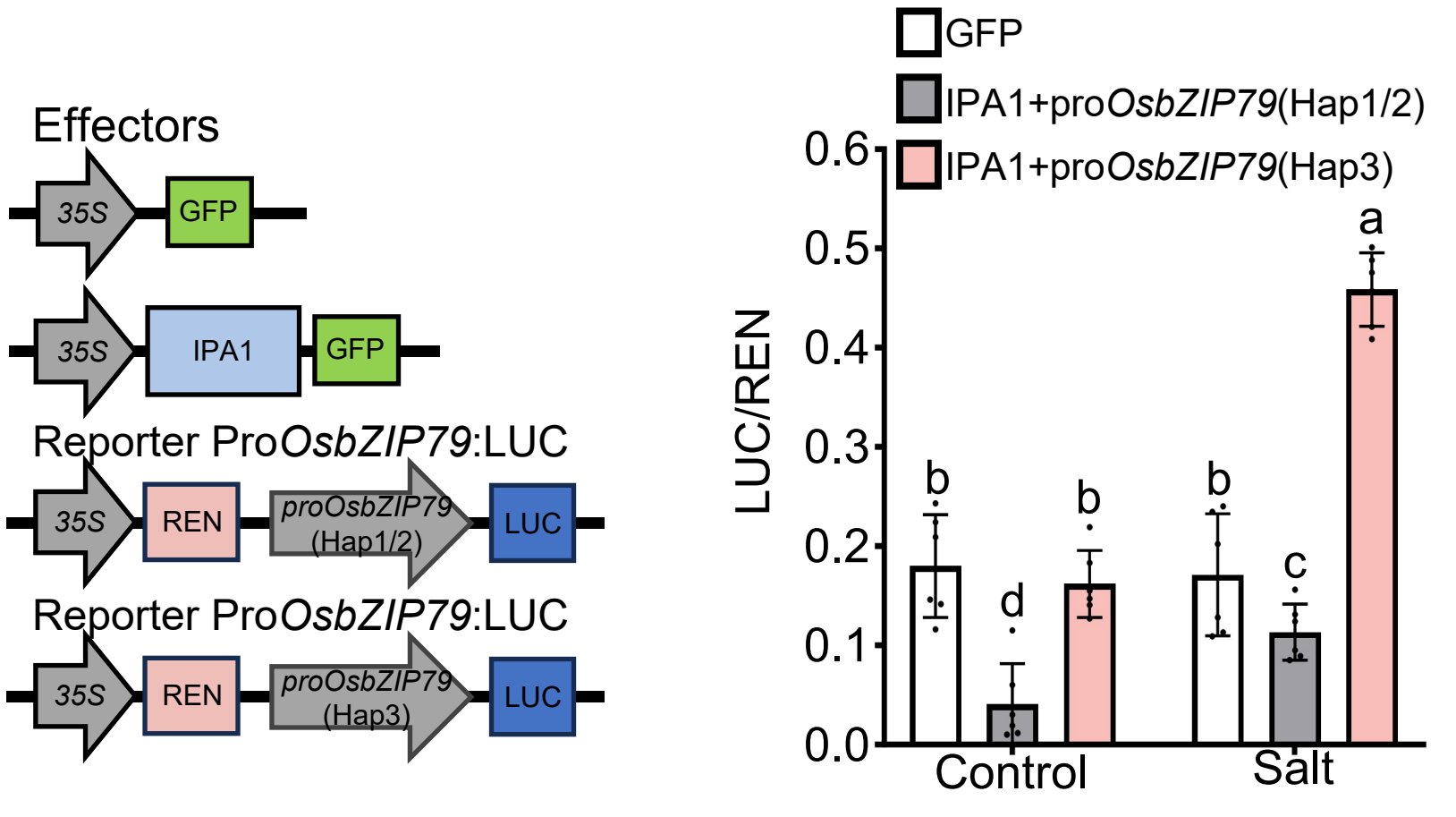

Effectors
35S
GFP
35S
IPA1
GFP
Reporter ProOsbZIP79:LUC
35S
REN
proOsbZIP79 (Hap1/2)
LUC
Reporter ProOsbZIP79:LUC
35S
REN
proOsbZIP79 (Hap3)
LUC
GFP
IPA1+proOsbZIP79(Hap1/2)
IPA1+proOsbZIP79(Hap3)
LUC/REN
0.0
0.1
0.2
0.3
0.4
0.5
0.6
b
d
b
b
c
a
Control
Salt

Figure S18

A

```
vg1102464952 (A) MGEASSSSGHPRQNPHVLGYGFHGAMPNSLPSANLFEQQGGANYFGELEEALMQQVATLRRTQQTATTTS 70
vg1102464952 (G) MGEASSSSGHPRQNPHVLGYGFHGAMPNSLPSANLFEQQGGANYFGELEEALMQQVATLRRTQQTATTTS 70

vg1102464952 (A) TLHHGDTTPFSTTATAAATARPPPTLDIFPSWPMRSLHTPKEGSNVTADTTDSESSSKNNSNQNASSDQH 140
vg1102464952 (G) TLHHGDTTPFSTTATAAATARPPPTLDIFPSWPMRSLHTPKEGSNVTADTTDSESSSKNNSNQNASSDQH 140

vg1102464952 (A) VLVGDMAGQFDQIPQQEQHKKMATNSPTHSSKTGKALDPKTMRRLAQNREAARKSRLRKKAYIQQLESSK 210
vg1102464952 (G) VLVGDMAGQFDQIPQQEQHKKMATNSPTHSSKTGKALDPKTMRRLAQNREAARKSRLRKKAYIQQLESSK 210

vg1102464952 (A) LKLAQMEQDIHRARSQGLLLGAPGGNTSSGAAMFDVDYARWLEEDSRRMAELHGGLHAHLPDSDLRAIVD 280
vg1102464952 (G) LKLAQMEQDIHRARSQGLLLGAPGGNTSSGAAMFDVDYARWLEEDSQRMAELHGGLHAHLPDSDLRAIVD 280

vg1102464952 (A) DTLTHYDHLFNLKGMAAKADVFHLITGMWATPAERCFLWMGGFRPSELLKTLTPQLDPLTEQQVVGICNL 350
vg1102464952 (G) DTLTHYDHLFNLKGMAAKADVFHLITGMWATPAERCFLWMGGFRPSELLKTLTPQLDPLTEQQVVGICNL 350

vg1102464952 (A) QQSSQQAEEALSQGLDQLHQSLAETVAGGSPLDDPNVGSFMGHMAIALGQLSNLEGFVIQADNLRQQTIH 420
vg1102464952 (G) QQSSQQAEEALSQGLDQLHQSLAETVAGGSPLDDPNVGSFMGHMAIALGQLSNLEGFVIQADNLRQQTIH 420

vg1102464952 (A) QMHRILTVRQAARCFLAIGEYHNRLRALSSLWASRPREILVADEGNCGELSIAAHPSESQYSAF 484
vg1102464952 (G) QMHRILTVRQAARCFLAIGEYHNRLRALSSLWASRPREILVADEGNCGELSIAAHPSESQYSAF 484
```

B

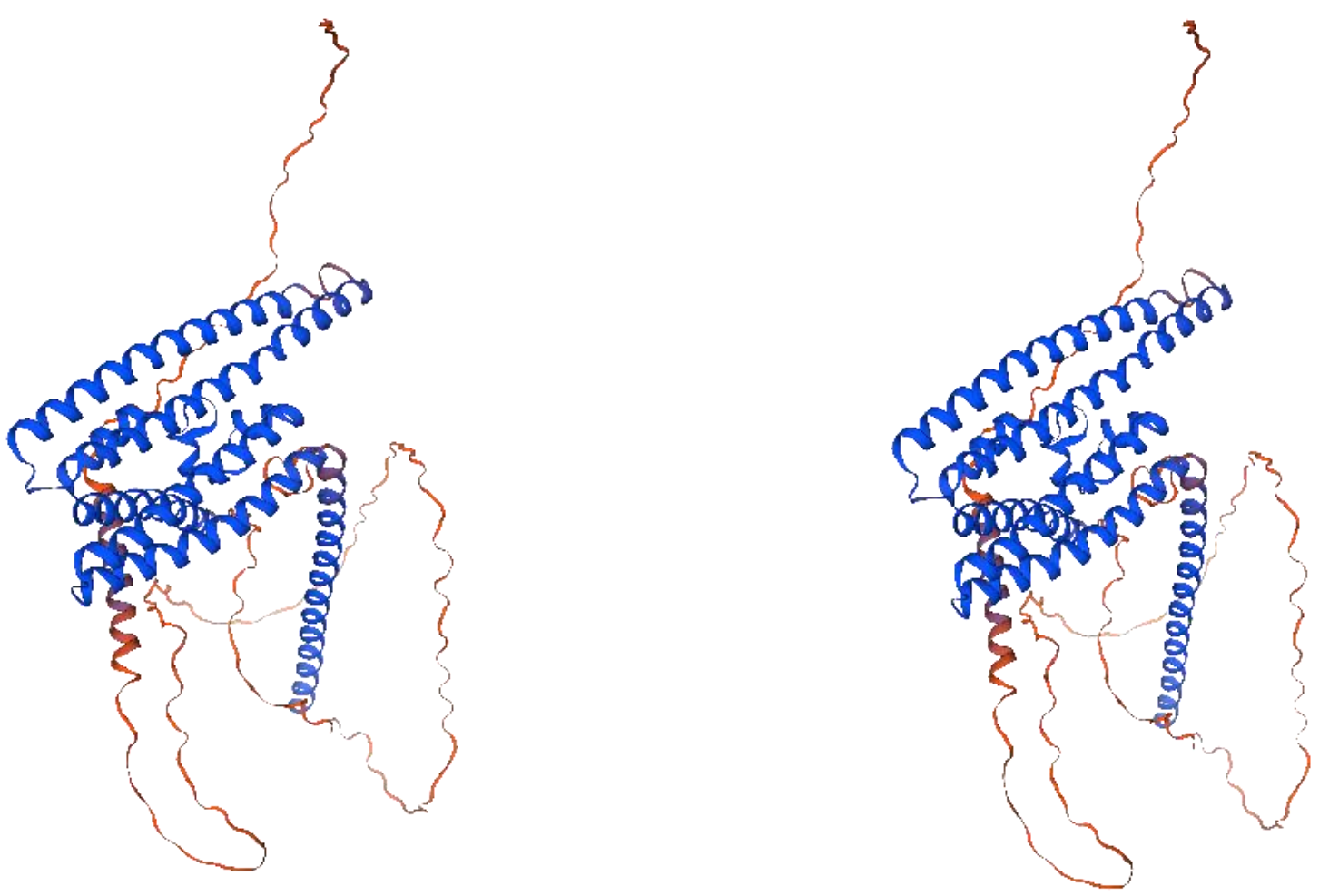

vg1102464952 (A) vg1102464952 (G)

Figure S19

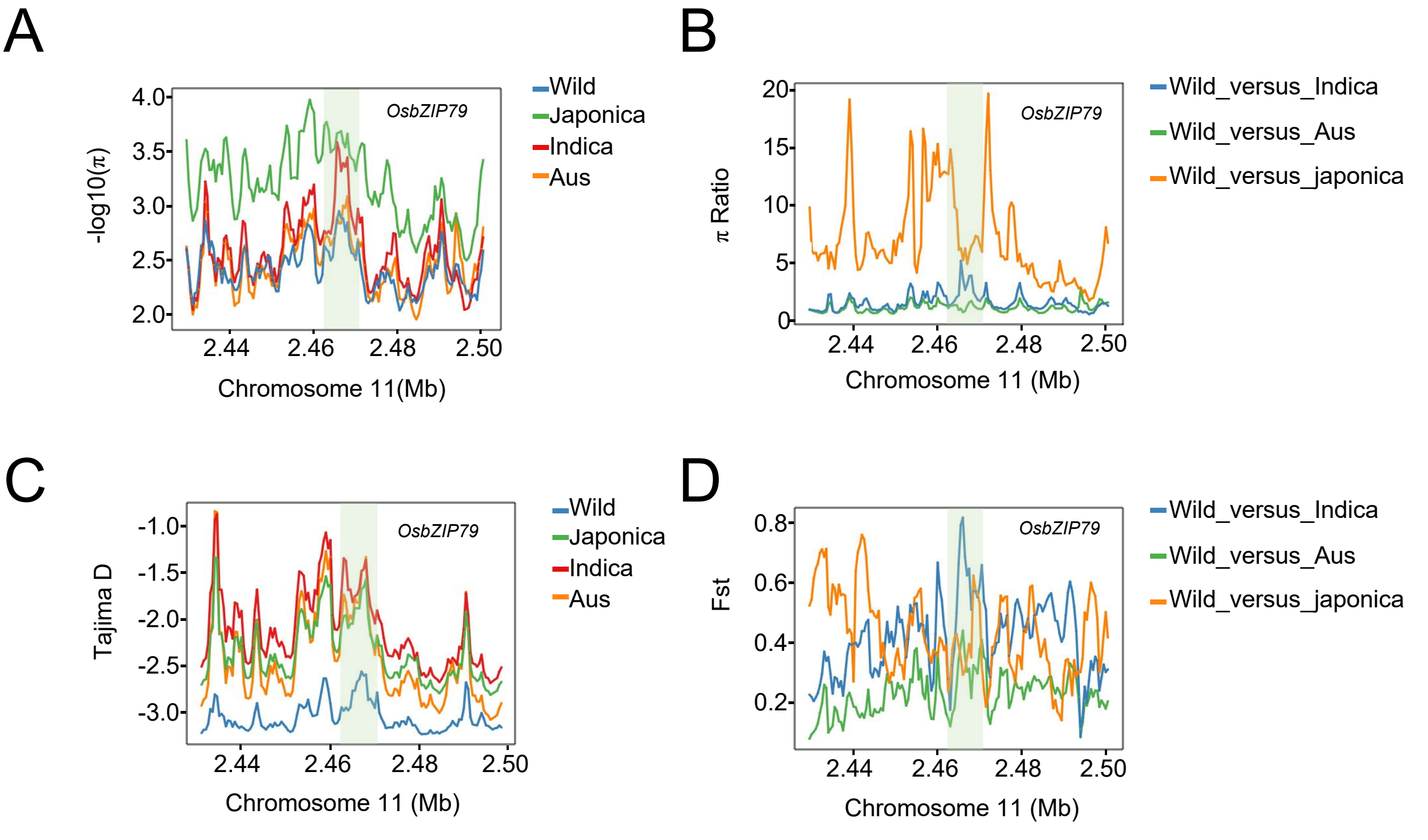

A
-log10(π)
4.0
3.5
3.0
2.5
2.0
OsbZIP79
2.44
2.46
2.48
2.50
Chromosome 11(Mb)
Wild
Japonica
Indica
Aus
B
π Ratio
20
15
10
5
0
OsbZIP79
2.44
2.46
2.48
2.50
Chromosome 11 (Mb)
Wild_versus_Indica
Wild_versus_Aus
Wild_versus_japonica
C
Tajima D
-1.0
-1.5
-2.0
-2.5
-3.0
OsbZIP79
2.44
2.46
2.48
2.50
Chromosome 11 (Mb)
Wild
Japonica
Indica
Aus
D
Fst
0.8
0.6
0.4
0.2
OsbZIP79
2.44
2.46
2.48
2.50
Chromosome 11 (Mb)
Wild_versus_Indica
Wild_versus_Aus
Wild_versus_japonica
